# Fluorescently Labeled Ceramides and 1-Deoxyceramides: Synthesis, Characterization, and Cellular Distribution Studies

**DOI:** 10.1021/acs.joc.2c02019

**Published:** 2022-11-28

**Authors:** Eduardo Izquierdo, Marta López-Corrales, Diego Abad-Montero, Anna Rovira, Gemma Fabriàs, Manel Bosch, José Luís Abad, Vicente Marchán

**Affiliations:** † Departament de Química Inorgànica i Orgànica, Secció de Química Orgànica, Universitat de Barcelona (UB), Martí i Franquès 1-11, 08028Barcelona, Spain; ‡ Research Unit on BioActive Molecules, Departament de Química Biològica, Institut de Química Avançada de Catalunya (IQAC-CSIC), Jordi Girona 18-26, 08034Barcelona, Spain; § Unitat de Microscòpia Òptica Avanc̨ada, Centres Científics i Tecnològics, Universitat de Barcelona (UB), Av. Diagonal, 643, 08028Barcelona, Spain; ∥ Institut de Biomedicina de la Universitat de Barcelona (IBUB), 08028Barcelona, Spain

## Abstract

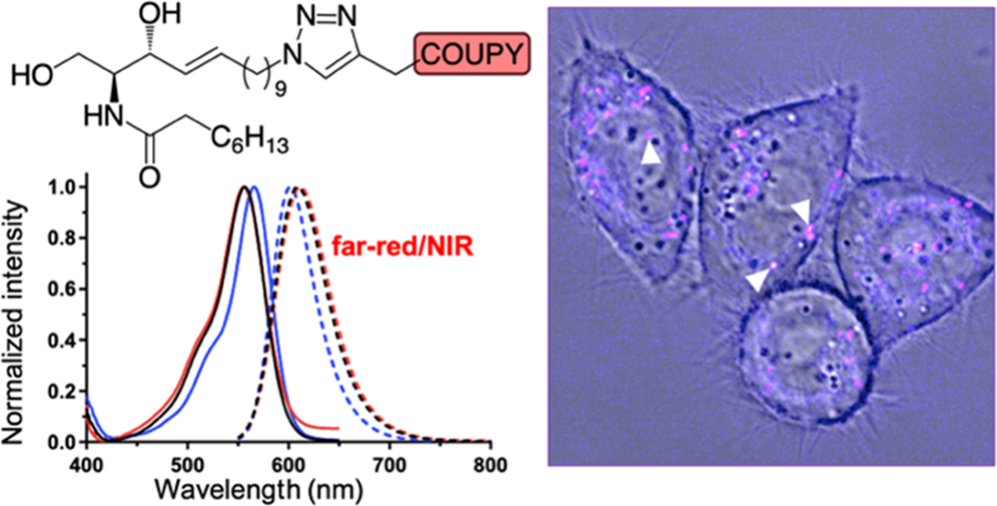

Ceramides (Cer) are bioactive sphingolipids that have been proposed as potential disease biomarkers since they are involved in several cellular stress responses, including apoptosis and senescence. 1-Deoxyceramides (1-deoxyCer), a particular subtype of noncanonical sphingolipids, have been linked to the pathogenesis of type II diabetes. To investigate the metabolism of these bioactive lipids, as well as to have a better understanding of the signaling processes where they participate, it is essential to expand the toolbox of fluorescent sphingolipid probes exhibiting complementary subcellular localization. Herein, we describe a series of new sphingolipid probes tagged with two different organic fluorophores, a far-red/NIR-emitting coumarin derivative (COUPY) and a green-emitting BODIPY. The assembly of the probes involved a combination of olefin cross metathesis and click chemistry reactions as key steps, and these fluorescent ceramide analogues exhibited excellent emission quantum yields, being the Stokes’ shifts of the COUPY derivatives much higher than those of the BODIPY counterparts. Confocal microscopy studies in HeLa cells confirmed an excellent cellular permeability for these sphingolipid probes and revealed that most of the vesicles stained by COUPY probes were either lysosomes or endosomes, whereas BODIPY probes accumulated either in Golgi apparatus or in nonlysosomal intracellular vesicles. The fact that the two sets of fluorescent Cer probes have such different staining patterns indicates that their subcellular distribution is not entirely defined by the sphingolipid moiety but rather influenced by the fluorophore.

## Introduction

Sphingolipids (SLs) are one of the main families of lipids in eukaryotic cells. SLs are essential structural components of cell membranes, but some of them also play capital roles in the regulation of key biological processes.^1^ In particular, ceramides (Cer) are a subgroup of bioactive SLs that have been found to induce apoptosis in response to various cell stress-inducing agents.^2,3^ Cer are also involved in the regulation of cell senescence, differentiation, and autophagy.^4^ Furthermore, recent reports suggest that Cer are linked to the progression of human diseases such as cancer,^5^ Alzheimer’s disease,^6^ or diabetes^7^ and, therefore, Cer have been proposed as potential disease biomarkers.^8^

**Scheme 1:**
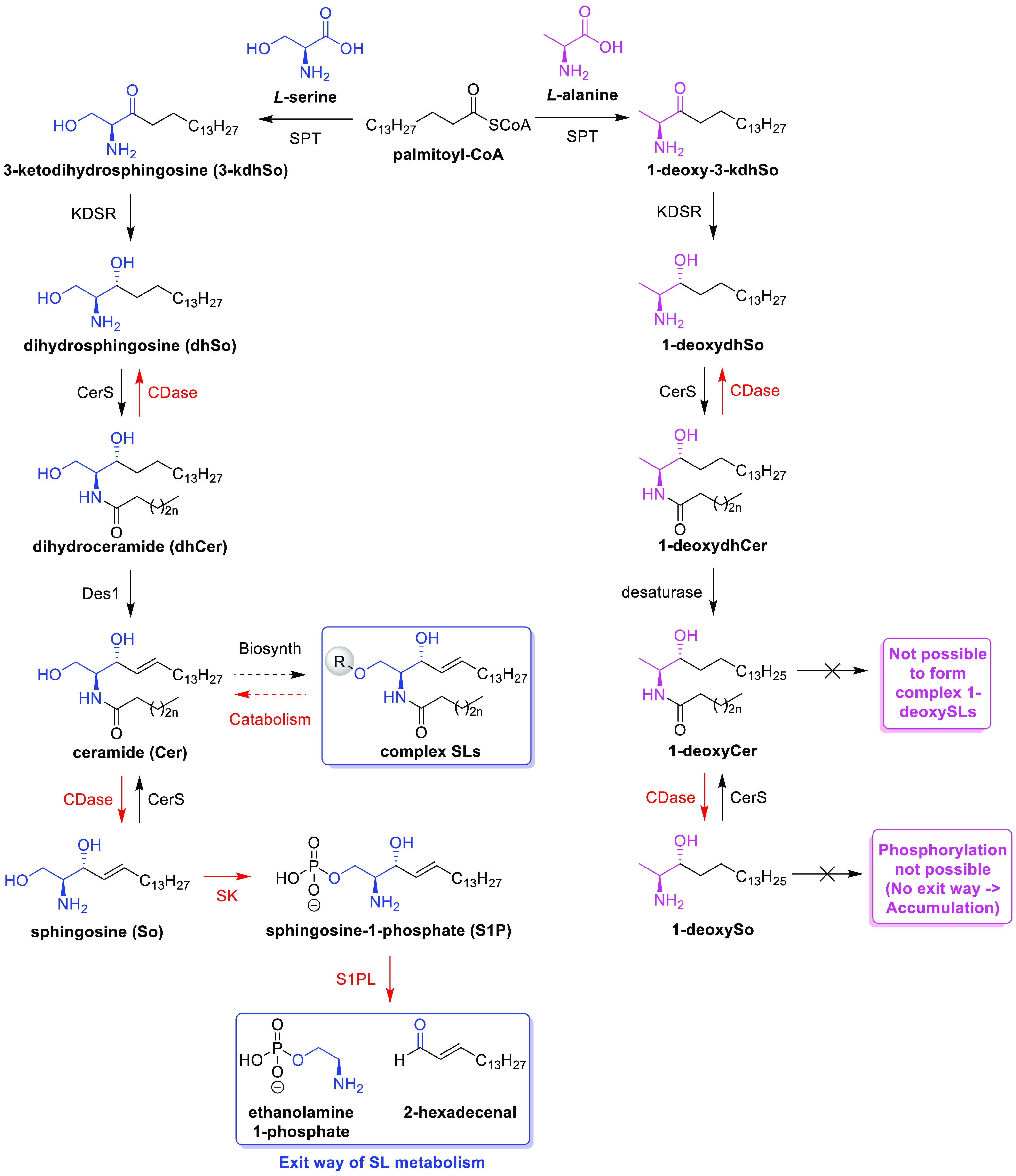
Differential Metabolism of Canonical Sphingolipids (Blue) and That of 1-Deoxysphingolipids (Pink) Abbreviations: SPT: serine palmitoyl-transferase, CerS: ceramide synthase, Des1: dihydroceramide desaturase, CDase: ceramidase, KDSR: 3-ketodihydrosphingosine reductase, SK: sphingosine kinase, S1PL: sphingosine-1-phosphate lyase. Adapted from ref (12).

The different families of SLs arise from the metabolic modification of a basic backbone known as the sphingoid base. Canonical SLs derive from the sphingoid base (2*S*,3*R*)-2-amino-1,3-octadecanediol, commonly known as sphinganine (dihydrosphingosine, dhSo). In cells, the sphingoid backbone is built during the “*de novo*” biosynthesis of SLs, which begins with the condensation of l-serine and palmitoyl-CoA, a reaction catalyzed by the serine-palmitoyltransferase (SPT) enzyme (Scheme 1). SPT can also use other substrates, such as L-alanine, leading to the formation of noncanonical SL species called 1-deoxysphingolipids (1-deoxySLs). Specific point mutations in SPT inducing a higher preference of the enzyme for alanine, which result in the accumulation of neurotoxic 1-deoxySL species, are the cause of the rare Hereditary Sensory and Autonomic Neuropathy type 1 (HSAN1).^9^ Since their first discovery,^10,11^ 1-deoxySLs have drawn much attention from lipid scientists; however, little is still known about their metabolism, function, subcellular localization, and dynamics in cell membranes. It is therefore of great interest to develop novel chemical tools to gain insight into the role of (1-deoxy)SLs in human diseases, which will allow us to find new opportunities for therapeutic intervention and diagnosis.

Fluorescently labeled SLs have found multiple applications in biology, including the visualization of cellular events involving SLs,^13^ the study of biophysical properties and dynamics of SLs in biological membranes, and the development of assays to monitor the activity of enzymes responsible for their metabolism.^14^ In this context, we have recently shown the potential of fluorescently labeled 1-deoxyCer and 1-deoxydhSo probes to study the activity of acid ceramidase (AC) and ceramide synthase (CerS) enzymes, respectively.^15,16^ 1-DeoxySLs do not undergo the same metabolic reactions as canonical SLs; the lack of the C1-OH group prevents the 4,5-desaturation and avoids the formation of more complex SLs.^15^ The higher metabolic stability of 1-deoxySLs, compared to that of canonical SLs, has been shown to confer robustness to particular biological assays but could also be exploited for imaging applications that require longer observation times.

Figuring out how ceramides are distributed and arranged in different organelles and how SLs arranging is integrated with protein sorting and trafficking is a challenge and interesting target. For that reason, choosing a right fluorophore is of utmost importance when designing fluorescently labeled SL probes. Ideal fluorophores should present high photostability in physiological media, high quantum yield and brightness, and large Stokes’ shifts, and their absorption and emission maxima should be located in the visible region of the electromagnetic spectrum.^13^ Furthermore, the biological behavior of the fluorescently labeled probe should resemble that of its nonlabeled counterpart; hence, small-size fluorescent tags are usually preferred. Most common fluorescent moieties used to label SL probes are based on aromatic groups such as pyrene, dansyl, NBD, coumarin, and BODIPY.^17^ However, there are few examples of fluorescently labeled SLs incorporating far-red-emitting fluorophores,^18−22^ even though such fluorescent probes are very useful for microscopy studies since emission at longer wavelengths offers several advantages, including minimal autofluorescence interference, low light scattering, and minimal cell phototoxicity compared to UV and blue light.^23^

Recently, we have reported the development of a new family of far-red/NIR-emitting fluorescent dyes, known as COUPYs, which are based on a nonconventional coumarin scaffold. COUPY fluorophores display several interesting photophysical properties such as high photostability in aqueous media, moderate to high quantum yields, and large Stokes’ shifts.^23−25^ Due to their relatively small size, synthetic feasibility, and chemical robustness, they have been postulated as excellent candidates for labeling biomolecules without altering their biological functions. In this context, COUPY derivatives have been successfully conjugated by means of click chemistry on a solid support to Octreotide, an FDA-approved cyclooctapeptide with high affinity and selectivity for the somatostatin subtype 2 receptor (SSTR2), which is overexpressed on the membrane of several types of cancer cells.^26^ The attachment of the fluorophore moiety did not alter the biological properties of Octreotide and, thus, the resulting conjugates could be used for visualizing living HeLa cells overexpressing the SSTR2 receptor. In addition, some COUPY dyes can be used as efficient photosensitizers for photodynamic therapy, either alone or when conjugated with cyclometalated Ir(III) complexes.^27−29^

**Scheme 2:**
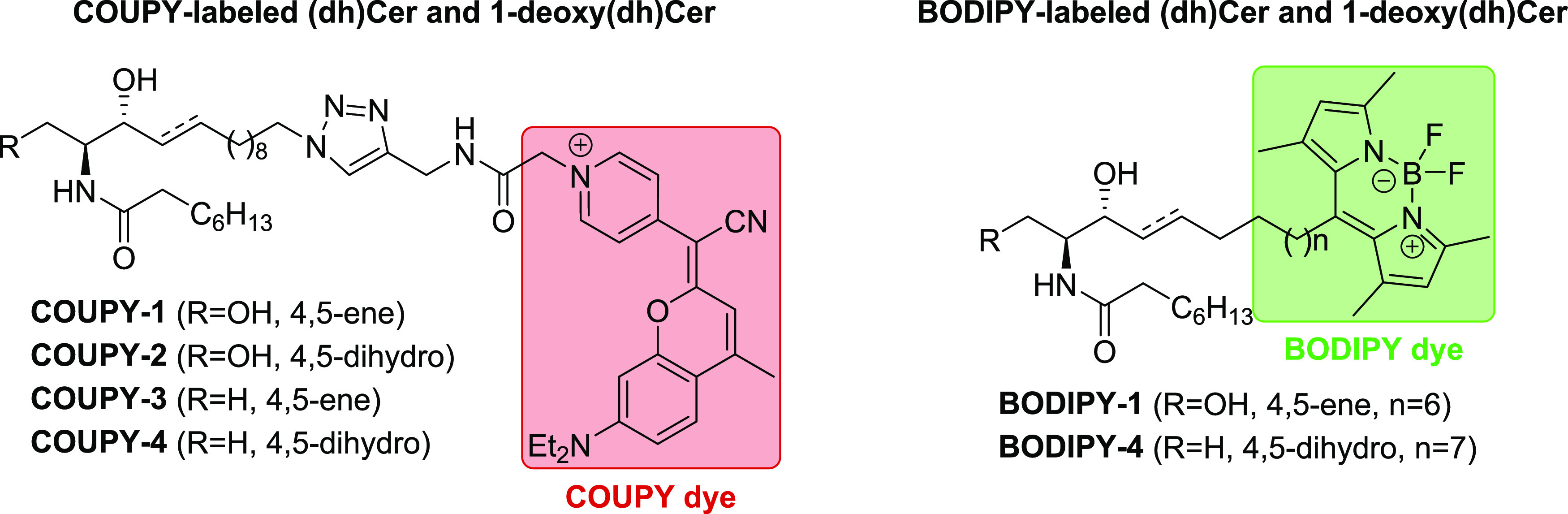
General Structure of the Fluorescently Labeled (Dihydro)ceramide and 1-Deoxy(dihydro)ceramide Probes Used in This Study

Herein we describe the synthesis, photophysical characterization, and subcellular distribution of a new series of fluorescent (dihydro)ceramides ((dh)Cer) and 1-deoxy(dihydro)ceramides (1-deoxy(dh)Cer) in which a far-red/NIR-emitting COUPY derivative has been incorporated into the sphingoid backbone by means of Cu(I)-catalyzed azide-alkyne cycloaddition (CuAAC) reaction (**COUPY-1** to **-4**, Scheme 2). Furthermore, the staining pattern of these novel COUPY-labeled SLs in live cells has been compared to that of the well-known green-emitting fluorescent lipid BODIPY-Cer^30^ (**BODIPY-1**, Scheme 2) and to that of its 1-deoxydh analogue (**BODIPY-4**, Scheme 2). We envision that the use of two structurally different fluorophores operating in different regions of the visible spectrum will ultimately allow us to perform dual-color fluorescence microscopy experiments to study the effect of the fluorophore on the subcellular distribution of the SL probes.

## Results and Discussion

### Synthesis of the Fluorescent Probes

**Scheme 3:**
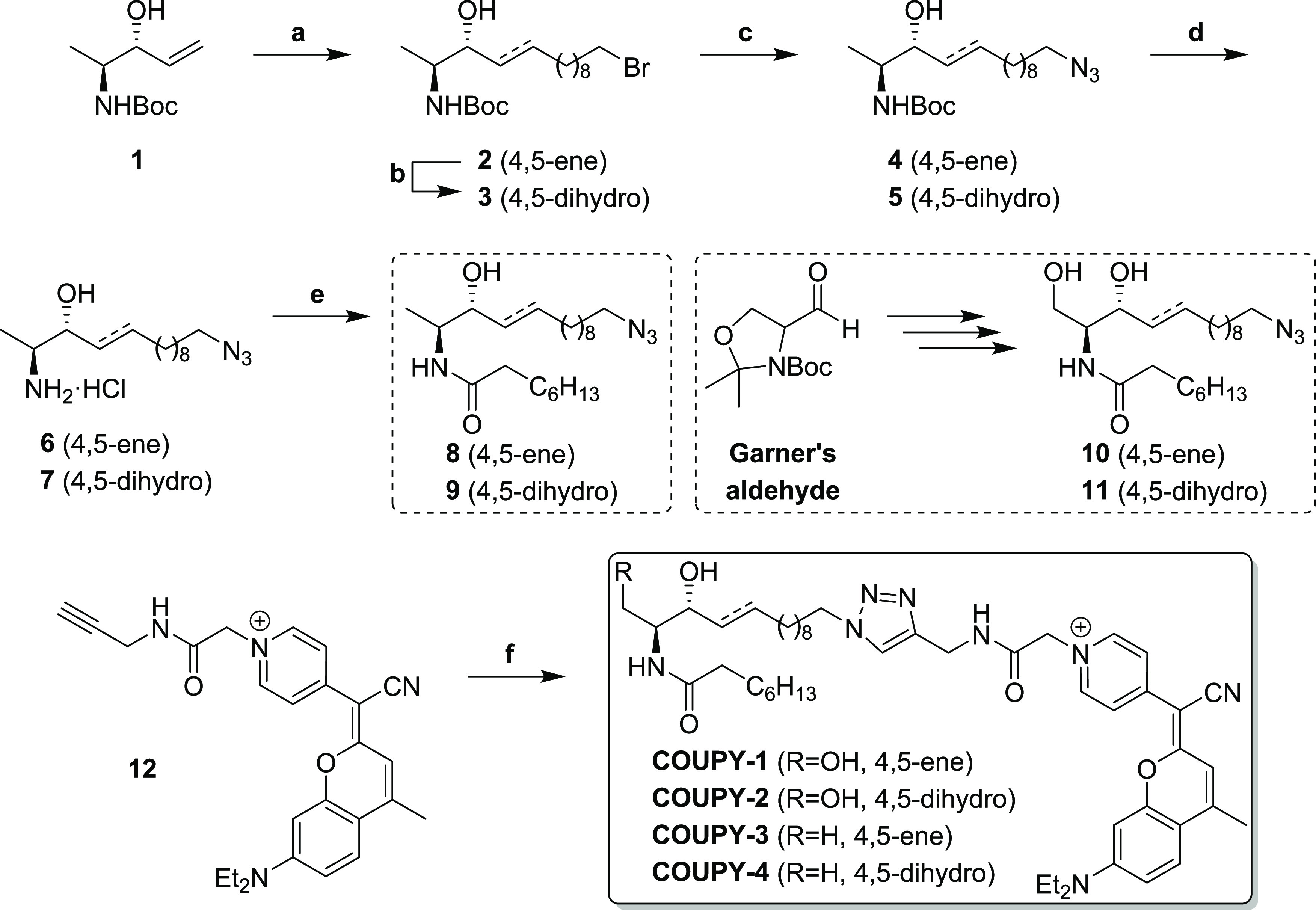
Synthesis of COUPY-Labeled Ceramide and 1-Deoxyceramide Probes Reagents and conditions: (a) 11-bromoundecene, 2nd gen. Grubbs catalyst, CH_2_Cl_2_, reflux, 5 h, 55%; (b) H_2_, Rh/Al_2_O_3_, MeOH, room temperature (rt), 24 h, 95%; (c) NaN_3_, dimethylformamide (DMF), 48 h, 60 °C, 85–95%; (d) AcCl, MeOH, 0 °C to rt, overnight, 88–90%; (e) octanoic acid, EDC·HCl, HOBt, Et_3_N, CH_2_Cl_2_, rt, 2 h, 70–72%; (f) azide precursor (**8**, **9**, **10** or **11**), CuSO_4_, ascorbic acid, ^*t*^BuOH/H_2_O (4:1, v/v), rt, overnight, 15–32%. The synthesis of compounds **1**, **10**, and **12** has been previously described in ref (34), ref (35), and ref (26), respectively.

COUPY-labeled (dh)Cer and 1-deoxy(dh)Cer probes **COUPY-1**–**4** were obtained by means of a copper(I)-catalyzed alkyne–azide cycloaddition (CuAAC) reaction between alkynyl-coumarin **12**(26) and the appropriate azide-tagged lipid ceramide (Scheme 3). The use of clickable fluorophores is a well-recognized strategy to develop fluorescent probes.^31−33^

The synthesis of azide precursors **8** and **9** was carried out as depicted in Scheme 3. Starting from allylic alcohol **1**,^34^ the olefin cross metathesis (OCM) reaction with 11-bromo-1-undecene using the second-generation Grubbs catalyst afforded the corresponding compound **2** as a highly *E*-enriched *E*/*Z* mixture, as evidenced by the C4(H)-C5(H) ^3^*J*_trans_ value of ∼15 Hz, which was in agreement with the literature.^34^ The *Z*-isomer and the homocoupling byproducts formed during the reaction were removed by means of SiO_2_ flash column chromatography. To obtain the saturated derivatives, the double bond across C4–C5 could be easily hydrogenated using a Rh/Al_2_O_3_ catalyst to afford the corresponding saturated intermediate **3**. Nucleophilic displacement of the terminal bromine atom in **2** and **3** with sodium azide afforded **4** and **5**, respectively. Formation of the azide intermediates was evidenced by the disappearance of the triplet signal at 3.40 ppm (−C**H**_**2**_Br) and the appearance of a new triplet signal at 3.24 ppm (−C**H**_**2**_N_3_) in the ^1^H NMR spectrum. Furthermore, the presence of the characteristic absorption band at 2092 cm^–1^ (−N=N^+^=N^–^ stretching) in the IR spectrum of **4** and **5** also confirmed the presence of the azide group (data not shown). Then, acid-mediated removal of the Boc amino protecting group, followed by carbodiimide-promoted amide coupling of the resulting free amine with octanoic acid, gave compounds **8** and **9**, respectively. Azide-tagged precursors **10** and **11** were obtained using Garner’s aldehyde as a starting material following a similar strategy, as described by Garrido et al.^35,36^ (Scheme 3).

To optimize the CuAAC reaction between azide-SL **8** and COUPY-alkyne **12** several conditions were assessed (Table 1). Initial attempts using CH_3_CN-H_2_O mixtures (entries 1 and 2) failed to produce the desired **COUPY-3** adduct, probably due to the lack of solubility of the lipid. However, when the reaction was carried out in a 4:1 (v/v) mixture of ^*t*^BuOH and H_2_O, **COUPY-3** was obtained in moderate yields after silica gel column chromatography (entry 3). Unfortunately, further attempts to improve the reaction yield by increasing the temperature (entry 4) or modifying the amount of catalyst (data not shown) were unsuccessful and resulted in the degradation of the coumarin precursor since several coumarin side-products were identified in the crude by liquid chromatography–mass spectrometry (LC–MS) analysis. The optimized reaction conditions were then used to synthesize the remaining COUPY-labeled probes **COUPY-1**, **COUPY-2**, and **COUPY-4** from the corresponding azido-SL precursors **10**, **11**, and **9**, respectively.

**Table 1:** Optimization of the CuAAC Reaction between Azide-SL **8** and COUPY-Alkyne **12**^a^^,^^b^

entry	solvent	CuSO_4_	sodium ascorbate	temp.	yield
1	CH_3_CN/H_2_O (1:1)	10 equiv	20 equiv	RT	no desired product^a^
2	CH_3_CN/H_2_O (2:1)	25 equiv	25 equiv	50 °C	only traces^a^
3	*^t^*BuOH/H_2_O (4:1)	25 equiv	25 equiv	RT	30%^b^
4	*^t^*BuOH/H_2_O (4:1)	25 equiv	25 equiv	50 °C	5% (degradation)^b^

a LC–MS observations.

b Isolated yield.

**Scheme 4:**
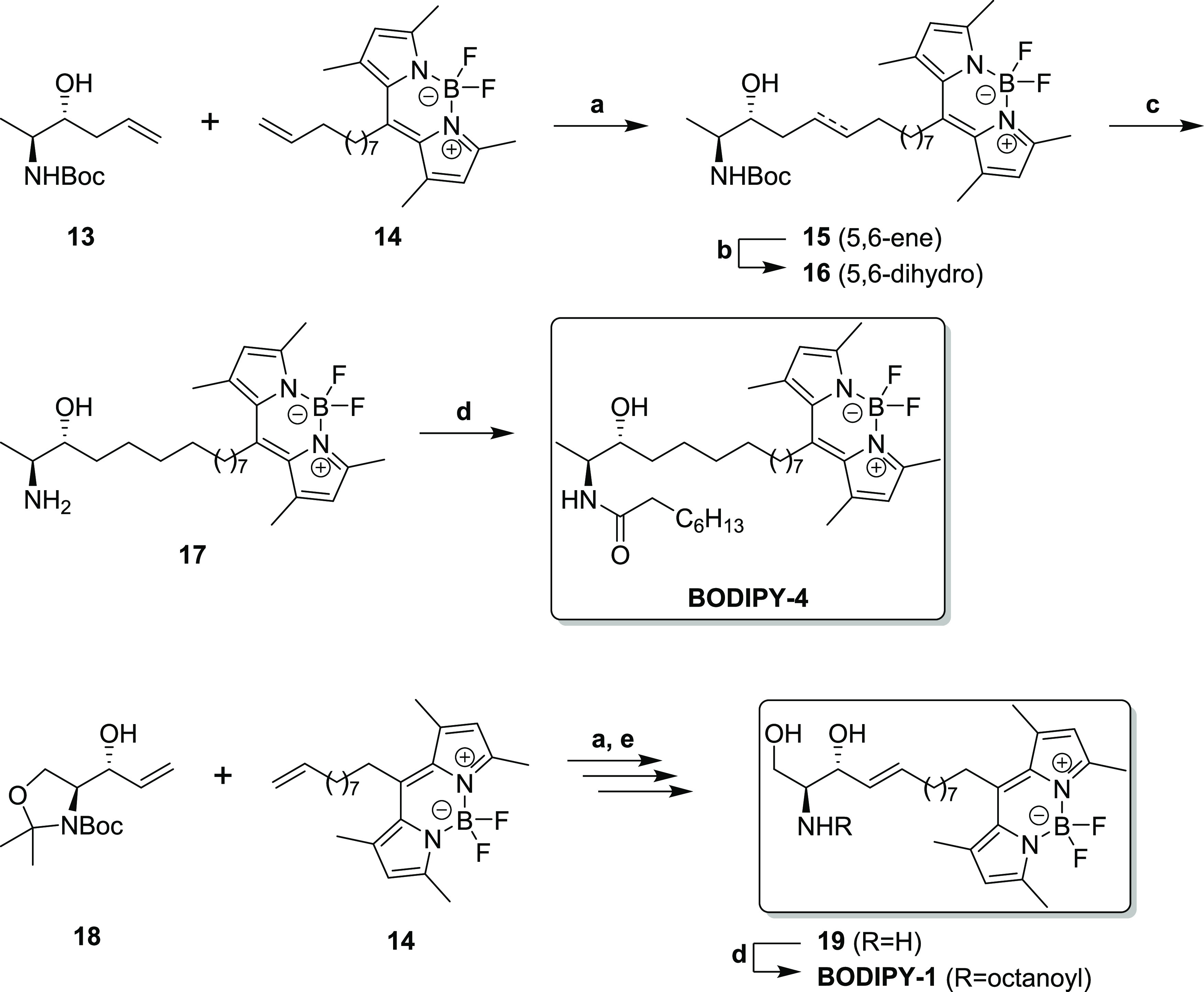
Synthesis of BODIPY-Labeled Ceramide and 1-Deoxyceramide Probes Reagents and conditions: (a) 2nd Gen. Grubbs catalyst, CH_2_Cl_2_, reflux, 5 h, 40%; (b) H_2_, Rh/Al_2_O_3_, MeOH, rt, 48 h, 97%; (c) BF_3_·OEt_2_, CH_2_Cl_2_, 0 °C to rt, 1 h, 83%; (d) octanoic acid, EDC·HCl, HOBt, Et_3_N, CH_2_Cl_2_, rt, 2 h, 63–64%; (e) (1) *p*-TsOH, MeOH, rt, 48 h, (2) BF_3_·OEt_2_, CH_2_Cl_2_, 0 °C to rt, 1 h, 83%. The synthesis of **13** has been described in ref (34), that of **14** and **19** in ref (37), and that of **18** in ref (34) and ref (38).

BODIPY-labeled (1-deoxy)(dh)Cer probes **BODIPY-1** and **BODIPY-4** were prepared following the methodology reported by Peters et al.^37^ As shown in Scheme 4, the OCM reaction between homoallyl alcohol **13**(34) and the alkenyl-BODIPY precursor **14**(37) lead to the corresponding intermediate **15**. Upon catalytic hydrogenation of the double bond using Rh/Al_2_O_3_, the Boc amino protecting group in **16** could be removed under mild conditions by treatment with BF_3_·Et_2_O complex to obtain compound **17**. Finally, carbodiimide-promoted amide coupling of the free amine of **17** with octanoic acid yielded the 1-deoxydhCer probe **BODIPY-4**. Analogously, the OCM reaction between allyl alcohol **18**(38) and the same alkenyl-BODIPY precursor **14**,^37^ followed by two acid-mediated steps; the removal of the isopropilidene group using catalytic amounts of TsOH in MeOH and the concomitant Boc group deprotection using the BF_3_·OEt_2_ according to the above conditions. Subsequent amide coupling of the resulting free amine with octanoic acid afforded the Cer probe **BODIPY-1** (Scheme 4).

### Photophysical Characterization of COUPY and BODIPY Fluorescent Probes

**Figure 1:**
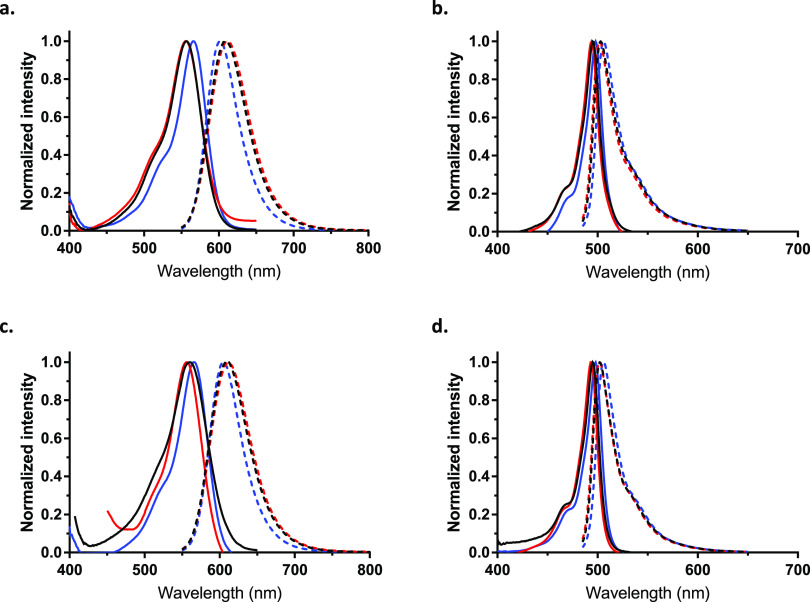
Comparison of the normalized absorption (solid lines) and fluorescence (dotted lines) spectra of compounds **COUPY-1** (a), **BODIPY-1** (b), **COUPY-4** (c), and **BODIPY-4** (d) in CH_3_OH (black lines), CH_3_CN (red lines), and CH_2_Cl_2_ (blue lines).

The spectroscopic and photophysical properties of the newly synthesized fluorescently labeled SLs were investigated to assess the effect of conjugating a (1-deoxy)(dh)Cer to the two structurally different fluorescent dyes, COUPY and BODIPY. The UV–vis absorption and emission spectra of the probes were recorded in three solvents of different polarities (see Figures 1 and S1). In general terms, as shown in Table 2, the photophysical properties of the different fluorescently labeled SL probes exclusively depended on the nature of the fluorophore (i.e., COUPY or BODIPY) and were not affected by the modification of the sphingoid backbone (i.e., 1-hydroxy/deoxy, 4,5-dihydro/4-ene).

**Table 2:**
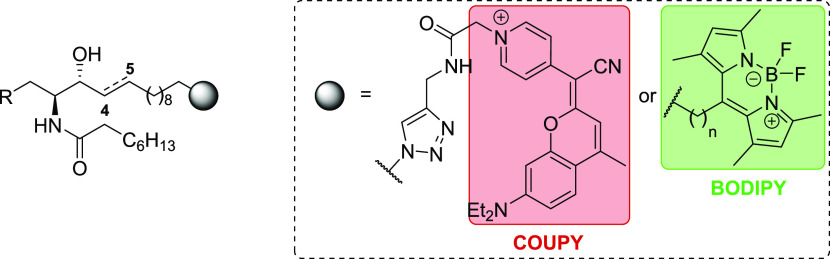
Photophysical Data of the Different COUPY- and BODIPY-Labeled SL Probes in Various Solvents

fluorophore	compound	R; C4–C5	solvent	λ_abs_ (nm)^a^	λ_em_ (nm)^b^	Φ_F_^c^	Stokes’ shift (nm)
**COUPY**	**COUPY-1**	R = OH; 4-ene	CH_3_OH	557	609	0.60	52
			CH_3_CN	556	612	0.39	56
			CH_2_Cl_2_	566	601	0.70	35
	**COUPY-2**	R = OH; 4,5-dihydro	CH_3_OH	557	608	0.42	51
			CH_3_CN	556	612	0.46	56
			CH_2_Cl_2_	566	604	0.70	38
	**COUPY-3**	R = H; 4-ene	CH_3_OH	556	609	0.41	53
			CH_3_CN	556	612	0.45	56
			CH_2_Cl_2_	567	600	0.71	33
	**COUPY-4**	R = H; 4,5-dihydro	CH_3_OH	560	608	0.58	48
			CH_3_CN	556	612	0.38	56
			CH_2_Cl_2_	566	604	0.66	38
**BODIPY**	**BODIPY-1**	R = OH; 4-ene	CH_3_OH	495	503	0.65	8
			CH_3_CN	494	502	0.58	8
			CH_2_Cl_2_	498	506	0.55	8
	**BODIPY-4**	R = H; 4,5-dihydro	CH_3_OH	495	502	0.47	7
			CH_3_CN	493	502	0.55	9
			CH_2_Cl_2_	498	506	0.49	8

a Wavelength of the absorption maximum.

b Wavelength of the emission maximum upon excitation at 475 nm for BODIPY-labeled probes and 540 nm for COUPY-labeled probes.

c Fluorescence quantum yields (Φ_F_) were measured by a comparative method using cresyl violet in EtOH (Φ_F_ = 0.54)^39^ as a reference for **COUPY-1**, **COUPY-2**, **COUPY-3**, and, **COUPY-4**. Fluorescein dissolved in 0.1 M aq NaOH (Φ_F_ = 0.92)^39^ was used as a reference in the case of **BODIPY-1** and **BODIPY-4**.

All four COUPY-labeled (1-deoxy)(dh)Cer probes **COUPY-1**–**4** displayed an intense absorption band in the visible region of the electromagnetic spectrum, with absorption maxima centered around 558 nm in CH_3_OH and CH_3_CN, and around 568 nm in CH_2_Cl_2_. This slight negative solvatochromism, that is, the blueshift in the absorption maximum wavelength when increasing the polarity of the solvent, had already been reported for similar COUPY dyes.^23,24^ As expected, the absorption spectra of BODIPY-labeled (1-deoxy)(dh)Cer probes **BODIPY-1** and **BODIPY-4** displayed a narrow and intense absorption band, with the absorption maxima around 496 nm, and almost negligible sensitivity to solvent polarity.

As anticipated, COUPY-labeled derivatives showed emission in the far-red to NIR region, with their emission maxima located at ∼602 nm in CH_2_Cl_2_, at 608 nm in CH_3_OH, and at 612 in CH_3_CN. The slight redshift of the emission maxima in more polar solvents was in contrast to the negative solvatochromism observed for the absorption maxima. Again, these observations are in agreement with the behavior of similar nonconjugated COUPY coumarins previously described by Gandioso et al.^23^ Conversely, the emission spectra of the BODIPY-labeled derivatives were characterized by a narrow emission band in the green region, with the emission maxima located at ∼504, that was not sensitive to solvent polarity. As a result, the Stokes’ shifts of the COUPY-labeled probes (∼36 nm in CH_2_Cl_2_ and 48–56 in CH_3_OH and CH_3_CN) were much higher than those of the BODIPY-labeled probes (7–9 nm). The use of fluorescent dyes with large Stokes’ shifts is especially important in FRET-type experiments to avoid undesired excitation and emission cross-talks.^16^

As detailed in Table 2, COUPY-labeled probes exhibited excellent fluorescent quantum yields (Φ_F_) both in nonpolar solvents (∼0.7 in CH_2_Cl_2_) and in polar solvents (∼0.4 in CH_3_CN and ∼0.5 in CH_3_OH), comparable to those of the parental *N*-methylpyridinium COUPY dye (Φ_F_ = 0.70 in CH_2_Cl_2_, Φ_F_ = 0.18 in CH_3_CN, Φ_F_ = 0.45 in EtOH).^25^ The two BODIPY-labeled derivatives also exhibited excellent Φ_F_ values, albeit slightly lower than their COUPY-labeled counterparts, irrespective of solvent polarity (0.47–0.65).

### Confocal Microscopy Studies in Live Cells

After showing that the photophysical properties of the two fluorescent dyes (COUPY and BODIPY) are not significantly influenced by the nature of the SL to which they are attached, we next investigated how and to what extent the different fluorophore tags and sphingoid polar heads determine the subcellular distribution of the fluorescent (dox)(dh)Cer probes.

**Figure 2:**
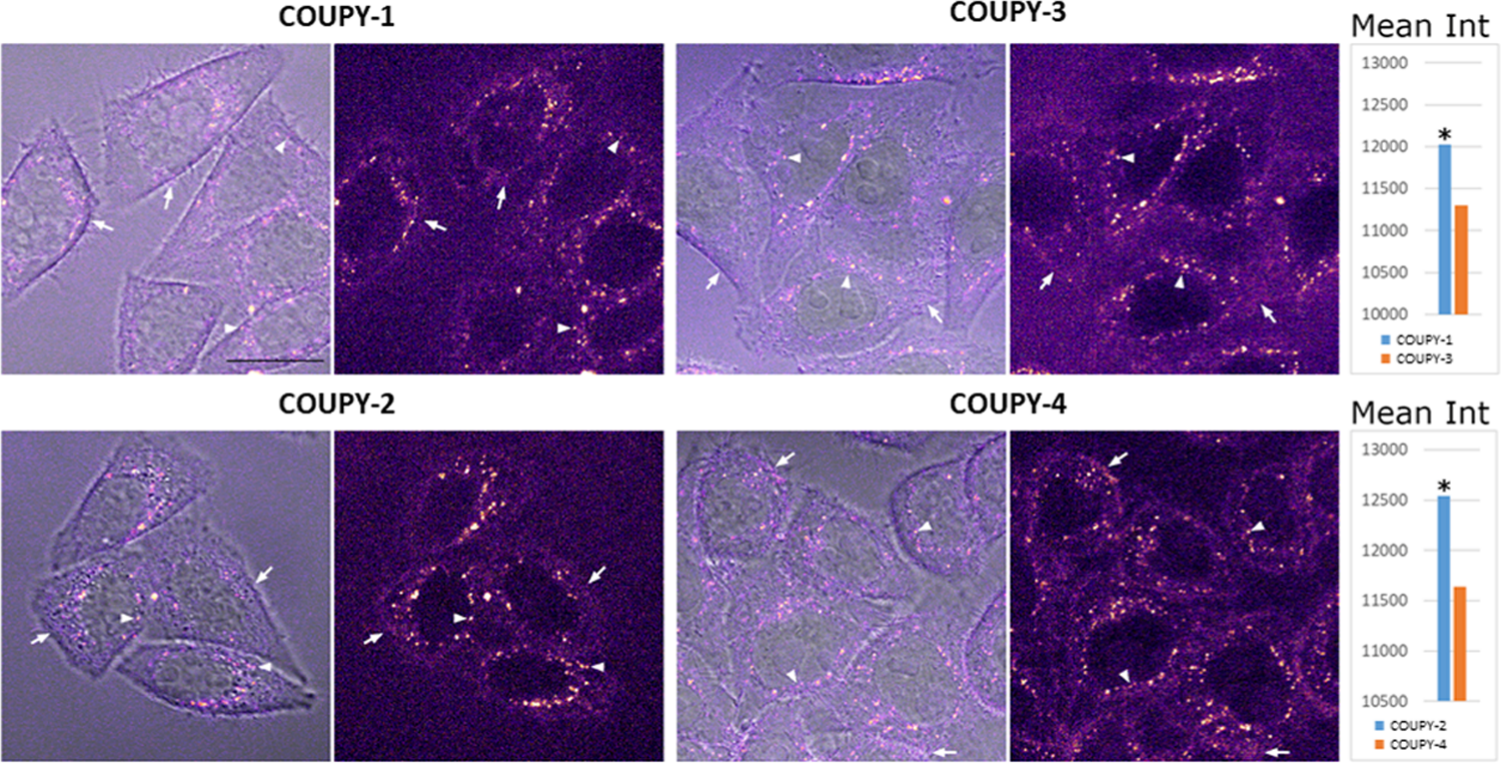
Comparison of the cellular uptake of the COUPY-labeled probes in HeLa cells after 30 min incubation at 1 μM. COUPY probes were excited using the 561 nm laser and emission was detected between 570 and 635 nm. For each compound: confocal plane showing the merge of brightfield and fluorescence image (left) and the fluorescence image alone (right, Fire LUT). Compound intensity is enhanced to emphasize the extracellular membrane staining. Arrows point out the extracellular membrane and arrowheads the intracellular vesicles. Scale bar: 20 μm. Graphs: mean intensity (arbitrary units) graphical representations of compound staining in the intracellular vesicles: Top: **COUPY-1** vs **COUPY-3**; bottom: **COUPY-2** vs **COUPY-4**. * T-student test, *p* < 0.01.

First, the cellular uptake of COUPY-labeled probes was studied in HeLa cells (1 μM, 30 min incubation) by confocal microscopy after irradiation with a yellow light laser (λ_ex_ = 561 nm). As shown in Figure 2, the four COUPY probes were correctly internalized and showed a very similar staining pattern. All of them mainly accumulated at intracellular vesicles and, to a lesser extent, at the extracellular membrane. Their staining pattern clearly differs from that of a hydrophobic nonconjugated *N*-hexyl pyridinium COUPY dye,^27,40^ which accumulates preferentially in mitochondria. Hence, according to these results, the subcellular distribution of the COUPY-labeled probes cannot be attributed to the effect of the fluorophore moiety. Moreover, it is worth noting that the COUPY-labeled SL probes described in this study did not produce any observable cell death during the experiment, differing again from the high cyto(photo)toxicity observed for the *N*-hexyl pyridinium COUPY dye.^27,40^

On the other hand, the four COUPY probes showed differences in the intensity of the intracellular vesicles staining pattern. Indeed, (dh)Cer probes **COUPY-1** and **COUPY-2** showed slightly higher mean intensity values compared to their 1-deoxy counterparts **COUPY-3** and **COUPY-4**. The weaker fluorescence intensity exhibited by the 1-deoxy COUPY probes could be attributed to a reduced cellular uptake.

**Figure 3:**
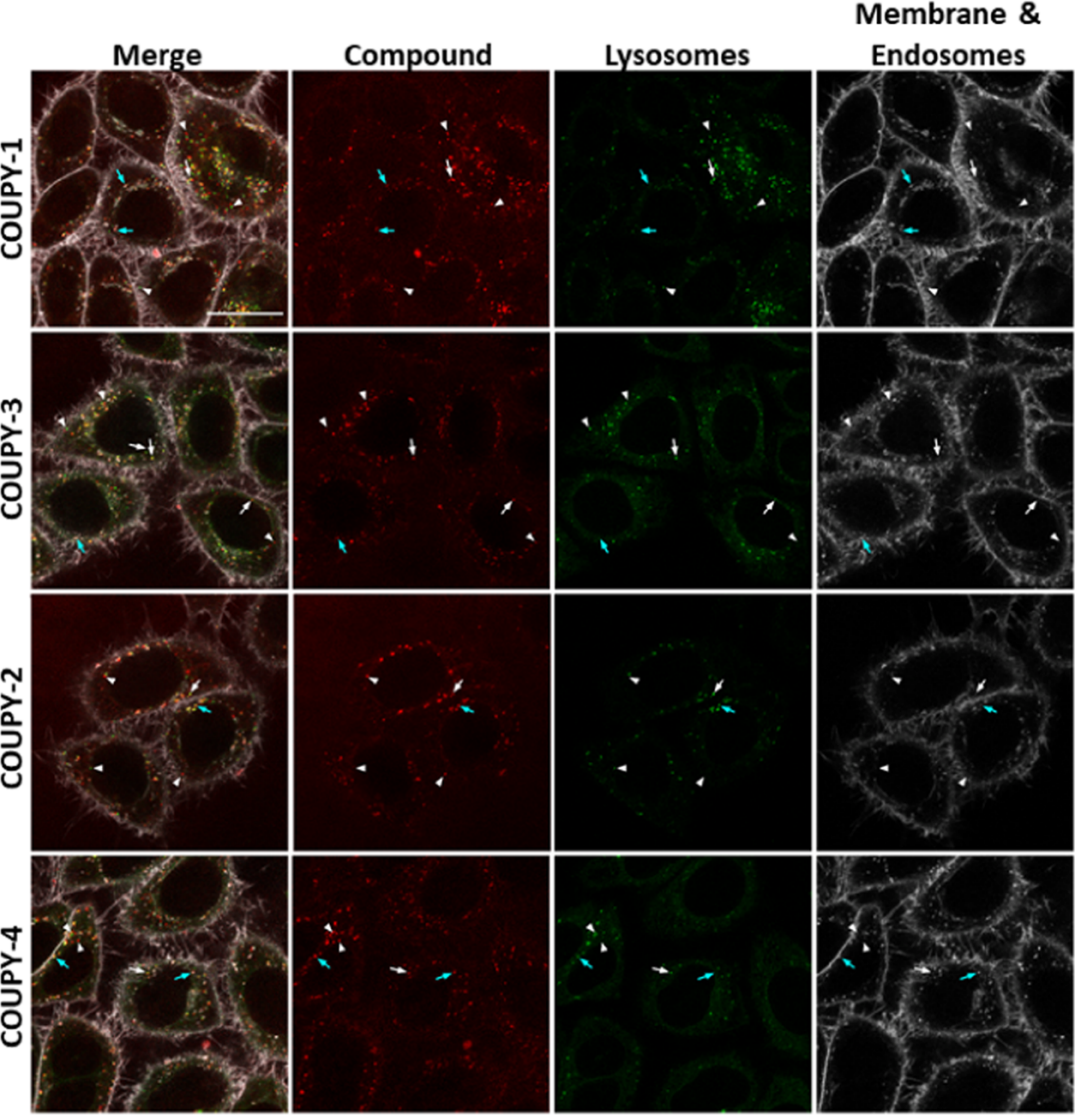
Colocalization studies of COUPY-labeled probes in HeLa cells. Confocal planes of HeLa cells cultured with the corresponding COUPY-labeled probe (red, 1 μM), LTG (green, 0.2 μM), and CellMask Deep Red (gray, 3 μg/mL) organelle trackers. COUPY probes were excited using the 561 nm laser, and emission was detected between 570 and 635 nm; LTG was excited using the 488 nm laser line, and emission was detected between 501 and 553 nm; CellMask Deep Red was excited using the 633 nm laser, and emission was detected between 638 and 735 nm. From left to right: confocal planes of merged, COUPY-labeled probe, LTG, and CellMask Deep Red images. From top to bottom: **COUPY-1**, **COUPY-3**, **COUPY-2**, and **COUPY-4**. Arrows point out positive colocalization of compound staining with LTG (white) or endosomes (cyan). Arrowheads point out no colocalizing intracellular vesicles. Scale bar: 20 μm.

To better understand the nature of the intracellular vesicles stained by COUPY-labeled probes, we performed co-localization experiments in HeLa cells using specific organelle markers: LysoTracker Green (LTG) for the lysosomes and CellMask Deep Red for the extracellular membrane and endosomes when internalized (Figure 3). Colocalization was measured using the Pearson’s correlation coefficient and Manders’ overlap (M1 and M2) coefficients.^41^ The Pearson’s coefficient measures the correlation between two images and their range of values from −1 to +1, being +1 the result obtained for a perfect correlation. On the other hand, Mander’s coefficients M1 and M2 calculate the intensities of one channel overlapping with the other. The values of these coefficients range from 0 to 1, and they are good indicators of colocalization even when the intensities between two channels clearly differ.^24^

In the colocalization studies with LTG, there was a low correlation between the LTG staining and that of the different COUPY-labeled probes, as evidenced by the values obtained for the Pearson’s coefficient (0.47–0.51, *n* > 15 cells) (Table S1, Supporting information). Moreover, Manders’ coefficients pointed out that less than 50% of the vesicles stained by the COUPY-labeled probes could be identified as lysosomes (M1: 23–40%), although almost half of LTG signal (45–65%) colocalized with the signal of the COUPY probes (M2). Interestingly, statistical analysis of the M1 coefficient values indicated that compound **COUPY-2** had a slightly higher affinity toward lysosomes than compound **COUPY-4**.

In the colocalization studies with CellMask Deep Red, we considered the fluorescence staining of CellMask corresponding to both the extracellular membrane and the endocytic vesicles. However, in the case of the COUPY-labeled probes, we only considered the signal corresponding to the staining of the intracellular vesicles since the signal at the extracellular membrane was too weak. This fact explains the low values obtained for Pearson’s coefficient (0.2–0.31, *n* > 15 cells), which indicates little or inexistent correlation between the staining of CellMask and that of the COUPY probes, and for the Manders’ M2 coefficient (0.03–0.06), which indicates that most of the signal of the CellMask channel does not overlap with the signal of the COUPY probes. On the other hand, the values obtained for Manders’ M1 coefficient (0.36–0.51) were slightly higher than those obtained for the colocalization with LTG and the statistical analysis of the values of all coefficients of **COUPY-1** and **COUPY-3** suggested that compound **COUPY-3** had a higher affinity toward endosomes than compound **COUPY-1**.

Altogether, these colocalization results indicate that almost all of the observed compound vesicles are either endosomes or lysosomes as the sum of the M1 coefficients with LTG and CellMask accounts for 88, 72, 90, and 59% of the staining of vesicles by probes **COUPY-1** to **COUPY-4**, respectively.

**Figure 4:**
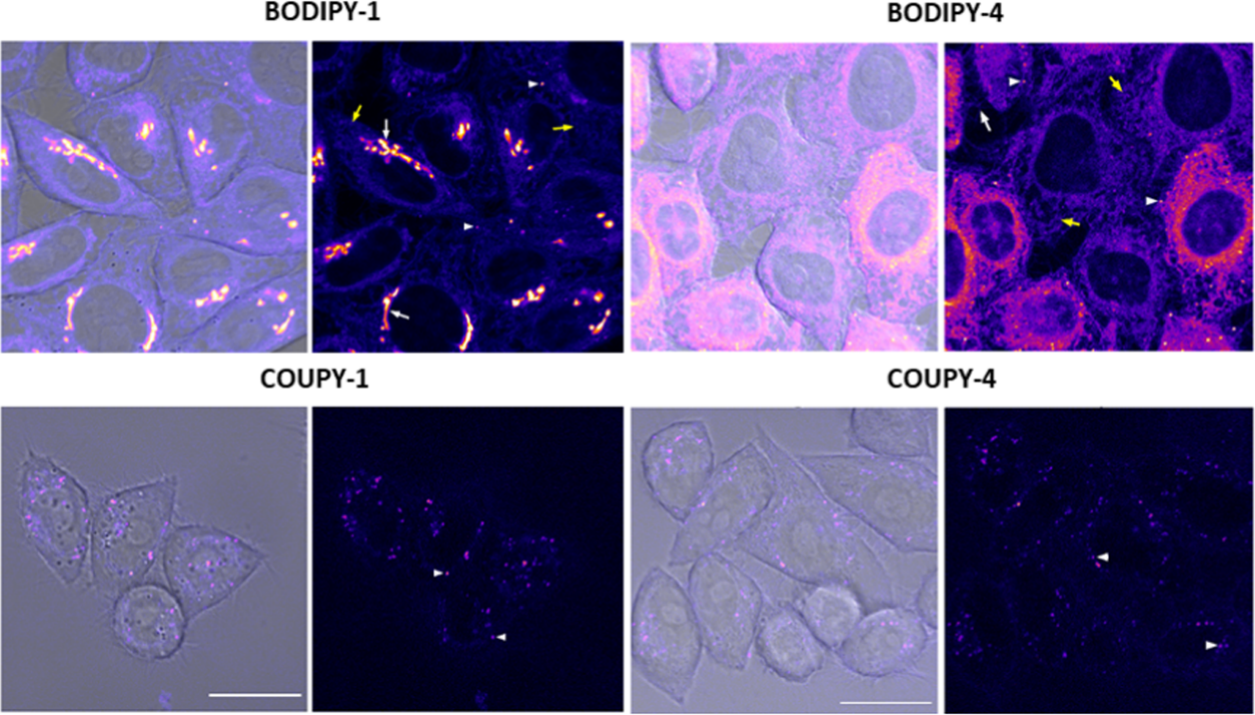
Comparison between the cellular uptake of the probes **BODIPY-1**, **BODIPY-4**, **COUPY-1**, and **COUPY-4** probes in HeLa cells after 30 min incubation at 1 μM. COUPY probes were excited using the 561 nm laser, and emission was detected between 570 and 635 nm; BODIPY probes were excited using the 488 nm laser line of the argon-ion laser, and emission was detected between 501 and 553 nm. For each compound: confocal plane showing the merge of bright field and fluorescence image (left) and the fluorescence image alone (right, Fire LUT). Compound intensity has not been enhanced. In **BODIPY-1**, fluorescence image, arrows point out the Golgi apparatus (white) and an intracellular net pattern (yellow). In **BODIPY-4**, fluorescence image, arrows point out the extracellular membrane (white) and an intracellular net pattern (yellow). In all cases, arrowheads point out the vesicles. Scale bar: 20 μm.

**Figure 5:**
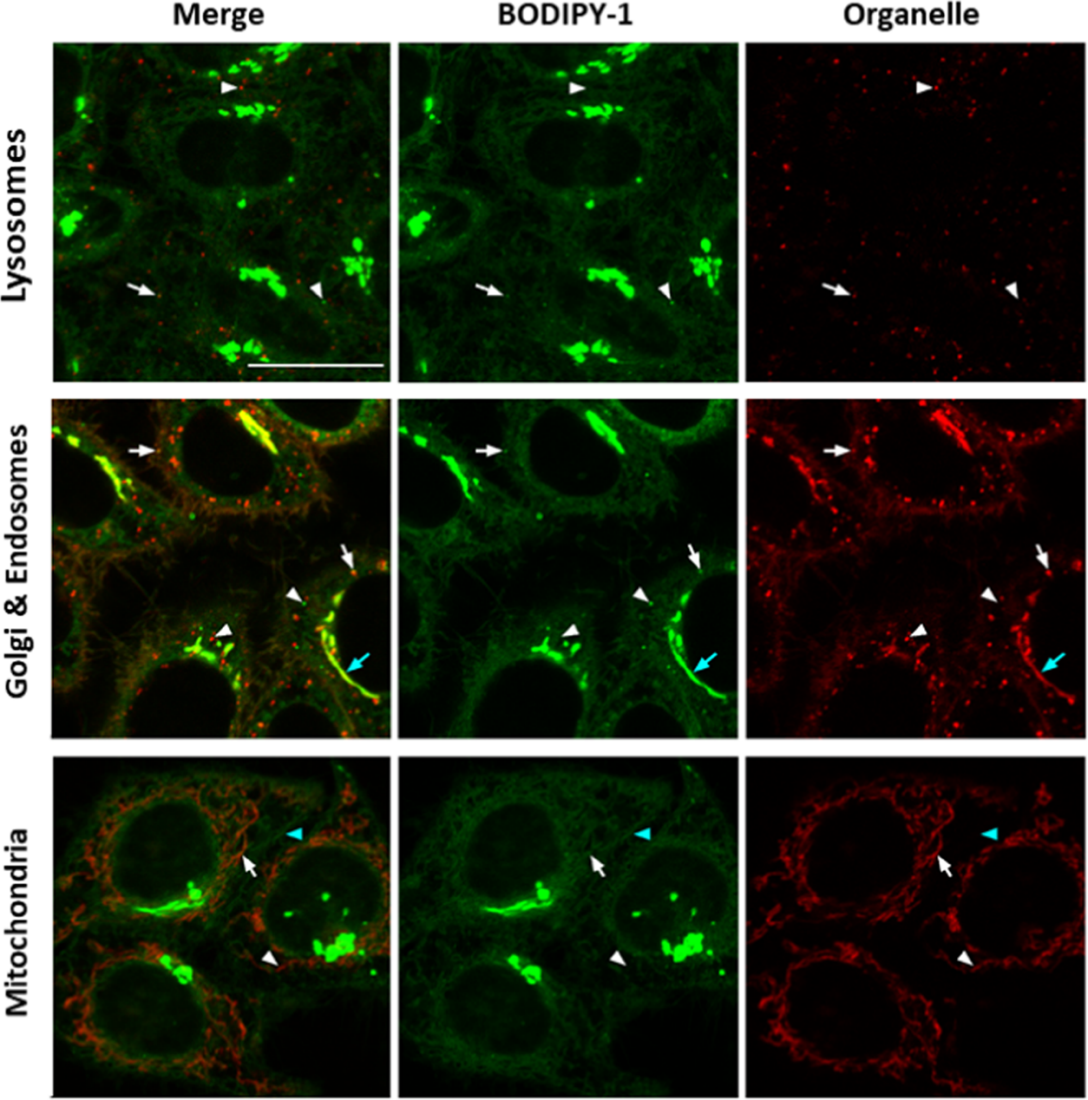
Colocalization studies of **BODIPY-1** in HeLa cells. Confocal planes of HeLa cells cultured with **BODIPY-1** (green, 1 μM) and LysoView650 (red, top, 1X), WGA555 (red, middle, 2 μg/mL), or MitoView650 (red, bottom, 100 nM) organelle trackers. **BODIPY-1** was excited using the 488 nm laser line of the argon-ion laser, and emission was detected between 501 and 553 nm; WGA555 was excited using the 561 laser, and emission was detected between 570 and 635 nm; LysoView650 and MitoView were excited using the 633 nm laser, and emission was detected between 638 and 735 nm. From left to right: merged images, **BODIPY-1** and organelle marker alone fluorescence images. White arrows and arrowheads point out positive or negative colocalization of **BODIPY-1** with the organelle marker, respectively. The cyan arrow in the central panel points out the Golgi apparatus. The cyan arrowhead (bottom) points out the cytoplasmatic signal of BODIPY-1 with an irregular net pattern extended beyond the mitochondria staining. Scale bar: 20 μm.

**Figure 6:**
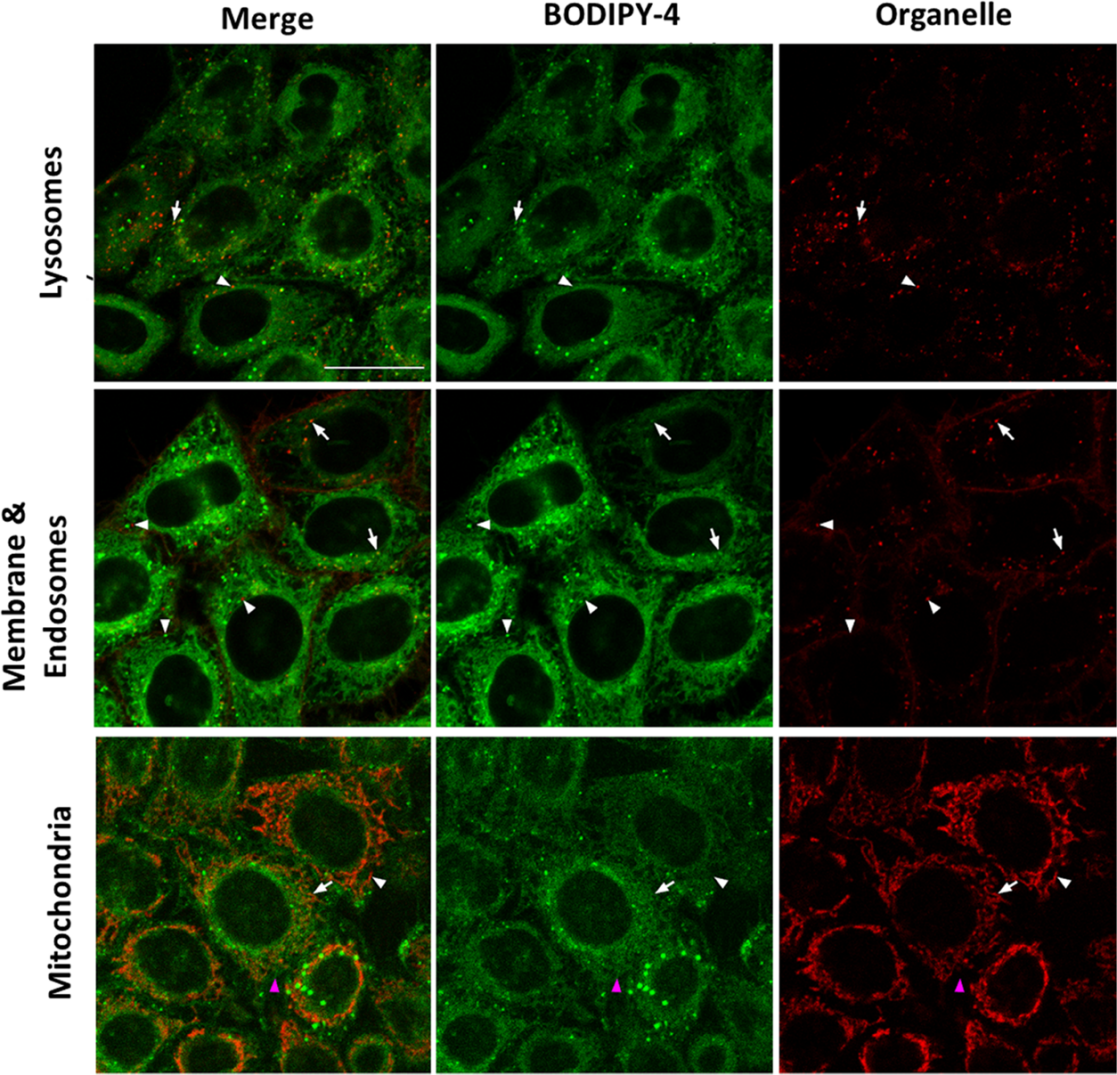
Colocalization studies of **BODIPY-4** in HeLa cells. Confocal planes of HeLa cells cultured with **BODIPY-4** (green, 1 μM) and LysoView650 (top, 1X), endosomes (center, 2 μg/mL), or MitoView650 (bottom, 100 nM) organelle trackers (all in red). **BODIPY-4** was excited using the 488 nm laser line of the argon-ion laser, and emission was detected between 501 and 553 nm; WGA555 was excited using the 561 laser, and emission was detected between 570 and 635 nm; LysoView650 and MitoView were excited using the 633 nm laser, and emission was detected between 638 and 735 nm. From left to right: merged images, **BODIPY-4** and organelle marker alone fluorescence images. White arrows and arrowheads point out positive or negative colocalization of **BODIPY-4** with the organelle marker, respectively. The magenta arrowhead (bottom) points out the cytoplasmatic signal of **BODIPY-4** with an irregular net pattern. Scale bar: 20 μm.

Next, we studied the cellular uptake of BODIPY-labeled probes in (**BODIPY-1** and **BODIPY-4**) HeLa cells (1 μM, 30 min incubation) by confocal microscopy after irradiation with the 488 nm laser line of the argon-ion laser and compared it to that of their COUPY-labeled counterparts (**COUPY-1** and **COUPY-4**, respectively). As shown in Figure 4, both BODIPY-labeled probes were correctly internalized. However, the two compounds displayed a different staining pattern, which also contrasted with that of their COUPY-labeled counterparts. While probes **COUPY-1** and **COUPY-4** stained almost exclusively intracellular vesicles, compound **BODIPY-1** was observed in a wider range of organelles (Figure 5), that is, it mainly accumulated in the Golgi apparatus and, to a lesser extent in some vesicles and in the extracellular membrane (not shown). On the other hand, compound **BODIPY-4** primarily stained intracellular vesicles, the mitochondria, and, less intensely, the extracellular membrane (Figure 6). In both cases, the staining pattern also seems to suggest endoplasmic reticulum (ER) localization.

To confirm the subcellular localizations of **BODIPY-1** and **BODIPY-4** we performed co-localization experiments in HeLa cells using specific organelle markers: LysoView650 for the lysosomes, WGA555 for the endosomes and Golgi when internalized and MitoView650 for the mitochondria (Figures 5 and 6). To ensure proper WGA555 internalization, cells were washed after incubation with the organelle marker and kept at 37 °C in nonsupplemented media for 45 min to 2 h.

In the case of **BODIPY-1**, the results showed little correlation (Table S2) between the staining of the compound and that of LysoView650 or WGA555 and therefore only a few of the intracellular vesicles stained by **BODIPY-1** could be identified as lysosomes or endosomes. On the other hand, longer incubations of WGA555 (45 min to 2 h) to ensure the staining of the Golgi apparatus showed a clear overlap with that of **BODIPY-1** and an increase in all of the colocalization coefficients. This is consistent with the localization in the Golgi apparatus of natural Cers and with the staining pattern of a previously described BODIPY-labeled Cer generated *in situ* by means of a SPAAC reaction between an ω-azidoCer and a BODIPY molecule containing a bicyclo[6.1.0]non-4-yne reactive group.^15^ In addition, most of the mitochondria stained with MitoView650 colocalized with **BODIPY-1** (Table S3), although the compound signal extended in the cytoplasm beyond the mitochondria in an irregular net pattern suggesting that **BODIPY-1** could also stain the ER.

In the case of **BODIPY-4**, only a small fraction of the intracellular vesicles stained by the compound corresponded to lysosomes or endosomes, as evidenced by the little correlation between the staining pattern of **BODIPY-4** and that of LysoView650 or WGA555 (Table S2). Moreover, some **BODIPY-4** vesicles were bigger in size compared to the lysosomes or endosomes indicating they were clearly a different type of organelles. This pattern is in disagreement with the subcellular distribution of a similar BODIPY-labeled 1-deoxydhCer derivative described by Casasampere et al,^15^ which primarily accumulated in the lysosomes.^15^ However, it is worth noting that in that case the 1-deoxydhCer analogue was labeled *in situ* through a SPAAC reaction with the BODIPY fluorophore after cellular internalization, whereas in our case, the cellular uptake of the BODIPY-ceramide conjugates was directly studied. Interestingly, in some cell lines, the authors also detected the presence of the probe in extra-lysosomal compartments that colocalized with Mitotracker (Mander’s coefficient M2 = 0.697). In this regard, the staining of mitochondria by BODIPY-labeled 1-deoxyCer has also been reported by Alecu et al.^42^ In accordance with these observations and similar to the results obtained for **BODIPY-1**, 78% of the signal detected in the mitochondria stained with MitoView650 colocalized with **BODIPY-4** (Table S3), although the extended staining signal of the compound in the cytoplasm beyond the mitochondria suggested that **BODIPY-4** could also stain the ER.

**Figure 7:**
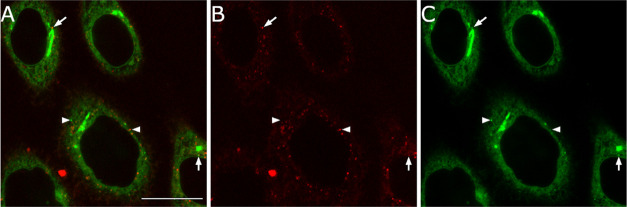
Colocalization of probes **COUPY-1** and **BODIPY-1**. Confocal plane of HeLa cells cultured with **COUPY-1** (1 μM, red) and **BODIPY-1** (1 μM, green) for 30 min at 37 °C. **COUPY-1** was excited using the 561 nm laser and emission was detected between 570 and 655 nm; **BODIPY-1** was excited using the 488 nm laser line of the argon-ion laser, and emission was detected between 501 and 553 nm. (A) Merged image; (B) fluorescence image of probe **COUPY-1**; and (C) fluorescence image of probe **BODIPY-1**. Arrows and arrowheads point out vesicles with or without colocalizing signals, respectively. Scale bar: 20 μm.

**Figure 8:**
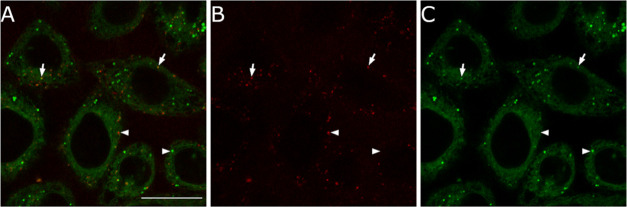
Colocalization of probes **COUPY-4** and **BODIPY-4**. Confocal plane of HeLa cells cultured with **COUPY-4** (1 μM, red) and **BODIPY-4** (1 μM, green) for 30 min at 37 °C. **COUPY-4** was excited using the 561 nm laser, and emission was detected between 570 and 655 nm; **BODIPY-4** was excited using the 488 nm laser line of the argon-ion laser, and emission was detected between 501 and 553 nm. (A) Merged image; (B) fluorescence image of probe **COUPY-4**; and (C) fluorescence image of probe **BODIPY-4**. Arrows and arrowheads point out vesicles with or without colocalizing signals, respectively. Scale bar: 20 μm.

In an effort to unveil whether the intracellular vesicles stained by COUPY-labeled probes were of the same nature as those stained by BODIPY-labeled probes, we decided to stain cells simultaneously with a COUPY probe and its BODIPY counterpart since the emission spectra of the two fluorophores do not overlap (see above). To this end, we incubated HeLa cells with both **COUPY-1** and **BODIPY-1** (1 μM, 30 min, 37 °C) and compared their staining arrangements by confocal microscopy. Interestingly, the two compounds showed no correlation with a Pearson coefficient smaller than 0.1 (*n* > 20 cells) and only few vesicles were observed in both channels (Figure 7). In a similar experiment, HeLa cells were incubated with both **COUPY-4** and **BODIPY-4** (1 μM, 30 min, 37 °C). Once more, the two compounds showed no correlation with a Pearson coefficient smaller than 0.15 (*n* > 30 cells) and only few vesicles were detected in both channels (Figure 8).

## Conclusions

In summary, we have developed and photophysically studied six novel fluorescently tagged SL probes: compounds **COUPY-1** (Cer), **COUPY-2** (dhCer), **COUPY-3** (1-deoxyCer), and **COUPY-4** (1-deoxydhCer) contain a far-red/NIR-emitting COUPY fluorophore, whereas compounds **BODIPY-1** (Cer) and **BODIPY-4** (1-deoxydhCer) contain a green-emitting BODIPY fluorophore. In the COUPY probes, the sphingoid backbone was obtained through an OCM reaction between an appropriate vinyl alcohol building block and a commercially available ω-bromoalkene. Then, the bromo derivative was converted into an azide, which was used to append the COUPY fluorophore through a CuAAC reaction with a readily available COUPY-alkyne precursor. In the BODIPY probes, the sphingoid backbone was also obtained through an OCM reaction between a similar alkenol building block and a BODIPY long-chain alkene precursor. As expected, the six probes exhibited excellent photophysical properties that were not substantially influenced by the modifications of the sphingoid backbone. Both COUPY and BODIPY probes displayed moderate to high fluorescence quantum yields in a range of organic solvents of varying polarity. Notably, COUPY probes emitted in the far-red/NIR region and had larger Stokes’ shifts than the BODIPY counterparts.

All of the COUPY and BODIPY probes showed a good cellular uptake in HeLa cells after 30 min incubations at 1 μM. Furthermore, the probes did not induce any observable cytotoxicity, which enabled the study of their subcellular distribution by confocal microscopy. In this sense, the four COUPY probes exhibited an almost identical staining pattern, that is they accumulated mainly in intracellular vesicles. After colocalization studies with organelle-specific trackers, we were able to determine that most of the vesicles stained by COUPY probes were either lysosomes or endosomes. Conversely, the two BODIPY probes showed very different staining patterns. On the one hand, compound **BODIPY-1** was primarily directed to the Golgi apparatus and, to a lesser extent to mitochondria and ER. On the other hand, compound **BODIPY-4** was detected in nonlysosomal intracellular vesicles, in mitochondria and, to a lesser extent in ER and in the extracellular membrane. Interestingly, the intracellular vesicles stained by **COUPY-1** and **COUPY-4** showed very little to inexistent colocalization with the staining pattern of **BODIPY-1** and **BODIPY-4**, respectively. The fact that the two sets of fluorescent SL probes have such different staining patterns suggests that the subcellular distribution of the probes is not entirely defined by the SL moiety, but it is also influenced by the fluorophore. The distribution of chemical probes within cells is strongly influenced by their acid–base, charge, and lipophilic/hydrophilic properties. For instance, COUPY dye has a positive charge, whereas BODIPY is globally neutral. Such differences in the structural properties of both dyes may account for the different behavior of the two sets of probes regarding cellular uptake, distribution, and diffusivity, as well as their mechanism of cell internalization.

Considering the excellent photophysical properties and cellular permeability of the fluorescent probes reported herein, we envision that they could find multiple applications in the field of sphingolipid biology. With this regard, we will next explore how these probes are metabolized by the enzymes of the SL pathway. The results of these studies will be reported in due course.

## Experimental Section

### Materials and Methods

Unless otherwise stated, common chemicals and solvents (HPLC-grade or reagent-grade quality) were purchased from commercial sources and used without further purification. A hot plate magnetic stirrer, together with an aluminum reaction block of the appropriate size, was used as the heating source in all reactions requiring heat. Aluminum plates coated with a 0.2 mm-thick layer of silica gel 60 F254 were used for thin-layer chromatography (TLC) analysis, whereas flash column chromatography purification was carried out using silica gel 60 (230–400 mesh). Proton (^1^H) and proton-decoupled carbon (^13^C{^1^H}) NMR spectra were recorded at 25 °C in a 400 MHz spectrometer using the deuterated solvent as an internal deuterium lock. The residual protic signal of chloroform, MeOH, or DMSO was used as a reference in ^1^H and ^13^C{^1^H} NMR spectra recorded in CDCl_3_, CD_3_OD, or DMSO-*d*_6_, respectively. Chemical shifts are reported in parts per million (ppm) in the δ scale, coupling constants in Hz, and multiplicity as follows: s (singlet), d (doublet), t (triplet), q (quartet), p (pentet/quintet), m (multiplet), dd (doublet of doublets), dq (doublet of quartets), br (broad signal), app (apparent). High-resolution electrospray ionization mass spectra (ESI-MS) were recorded on an instrument equipped with a single quadrupole detector coupled to a high-performance liquid chromatography (HPLC) system.

### Synthesis and Characterization of COUPY-Labeled Ceramides and 1-Deoxyceramides

#### (2*S*,3*R*,*E*)-*tert*-Butyl(14-bromo-3-hydroxytetradec-4-en-2-yl)carbamate (Compound **2**)

To a degassed solution of allylic alcohol **1**(34) (589 mg, 2.92 mmol) and 11-bromoundecene (2.72 g, 11.68 mmol) in CH_2_Cl_2_ (20 mL) was added portion-wise second-generation Grubbs catalyst (50 mg, 0.05 mmol). The resulting suspension was refluxed for 5 h. After cooling down to rt, the solvent was removed under reduced pressure obtaining a brown oil. The crude was purified by flash silica gel column chromatography (from 0 to 24%, EtOAc in hexanes) affording compound **2** as a colorless oil (652 mg, 55% yield). ^1^H NMR (400 MHz, CDCl_3_) δ (ppm) 5.70 (dt, *J* = 14.7, 7.1 Hz, 1H), 5.42 (dd, *J* = 15.5, 6.6 Hz, 1H), 4.66 (br s, 1H), 4.13–4.06 (m, 1H), 3.77 (br s, 1H), 3.40 (t, *J* = 6.9 Hz, 2H), 2.08–1.98 (m, 2H), 1.84 (dt, *J* = 14.4, 6.9 Hz, 2H), 1.44 (s, 9H), 1.39–1.23 (m, 12H), 1.07 (d, *J* = 6.9 Hz, 3H). ^13^C{^1^H} NMR (101 MHz, CDCl_3_) δ (ppm) 156.4, 134.1, 128.5, 79.8, 75.9, 51.2, 34.2, 32.9, 32.5, 29.5, 29.2, 28.9, 28.5, 28.3, 15.6. HRMS calcd for C_19_H_37_BrNO_3_^+^ [M + H]^+^: 406.1957, found 406.1949. [α]_D_^20^ = −2.3 (*c* = 1, CHCl_3_).

#### (2*S*,3*R*)-*tert*-Butyl(14-bromo-3-hydroxytetradecan-2-yl)carbamate (Compound **3**)

A solution of **2** (300 mg, 0.74 mmol) in degassed MeOH (25 mL) was hydrogenated at 1 atm and rt in the presence of Rh/Al_2_O_3_ (30 mg, 15% w/w) using a H_2_ balloon. Reaction evolution was monitored by ^1^H NMR. After stirring for 24 h, the catalyst was removed by filtration through a bed of Celite and the solid pad was rinsed with MeOH (3 × 10 mL). The combined filtrates were concentrated *in vacuo*, and the residue was subjected to flash chromatography on silica gel (from 0 to 24% EtOAc in hexanes) to afford **3** as a colorless oil (286 mg, 95% yield). ^1^H NMR (400 MHz, CDCl_3_) δ (ppm) 4.79 (br s, 1H), 3.72–3.66 (m, 1H), 3.66–3.58 (m, 1H), 3.40 (t, *J* = 6.9 Hz, 2H), 2.26 (br s, 1H), 1.84 (dt, *J* = 14.5, 6.9 Hz, 2H), 1.43 (s, 9H), 1.42–1.33 (m, 4H), 1.33–1.19 (m, 14H), 1.07 (d, *J* = 6.8 Hz, 3H). ^13^C{^1^H} NMR (101 MHz, CDCl_3_) δ (ppm) 155.9, 79.4, 74.4, 50.6, 34.1, 33.6, 32.9, 29.7, 29.61, 29.59, 29.57, 29.49, 28.8, 28.5, 28.2, 26.1, 14.3. HRMS calcd for C_19_H_39_BrNO_3_^+^ [M + H]^+^: 408.2108, found 408.2103. [α]_D_^20^ = −4.3 (*c* = 1, CHCl_3_).

#### (2*S*,3*R*,*E*)-*tert*-Butyl(14-azido-3-hydroxytetradec-4-en-2-yl)carbamate (Compound **4**)

Sodium azide (120 mg, 1.9 mmol) was added portion-wise to a solution of compound **2** (288 mg, 0.71 mmol) in anhydrous DMF (10 mL) and the resulting mixture was stirred at 60 °C under N_2_ atmosphere for 48 h. After cooling down to rt, the mixture was washed with water (50 mL) and extracted with Et_2_O (3 × 25 mL). The combined organic phases were dried over anhydrous MgSO_4_, filtered, and evaporated to dryness under reduced pressure. The crude was purified by flash silica gel column chromatography (from 0 to 15% EtOAc in hexanes) affording compound **4** as a colorless oil (230 mg, 87% yield).^1^H NMR (400 MHz, CDCl_3_) δ (ppm) 5.70 (dtd, *J* = 15.0, 6.8, 1.2 Hz, 1H), 5.42 (ddt, *J* = 15.4, 6.5, 1.4 Hz, 1H), 4.69 (br s, 1H), 4.10 (ddd, *J* = 6.6, 3.2, 1.1 Hz, 1H), 3.76 (br s, 1H), 3.24 (t, *J* = 7.0 Hz, 2H), 2.30 (br s, 1H), 2.03 (q, *J* = 7.3 Hz, 2H), 1.64–1.52 (m, 2H), 1.43 (s, 9H), 1.38–1.22 (m, 12H), 1.06 (d, *J* = 6.9 Hz, 3H). ^13^C{^1^H} NMR (101 MHz, CDCl_3_) δ (ppm) 156.4, 134.0, 128.5, 79.7, 75.8, 51.6, 51.2, 32.5, 29.52, 29.46, 29.2, 28.9, 28.5, 26.8, 15.6. HRMS calcd for C_19_H_37_N_4_O_3_^+^ [M + H]^+^: 369.2866, found 369.2865. [α]_D_^20^ = −4.7 (*c* = 1, CHCl_3_).

#### (2*S*,3*R*)-*tert*-Butyl(14-azido-3-hydroxytetradecan-2-yl)carbamate (Compound **5**)

Compound **5** was prepared following the same procedure as described for **4** starting from **3** (175 mg, 0.43 mmol) and sodium azide (84 mg, 1.30 mmol) in anhydrous DMF (10 mL). After purification by flash silica gel column chromatography (from 0 to 20% EtOAc in hexanes), compound **5** was obtained as a colorless oil (144 mg, 90% yield). ^1^H NMR (400 MHz, CDCl_3_) δ (ppm) 4.79 (d, *J* = 7.2 Hz, 1H), 3.73–3.65 (m, 1H), 3.67–3.57 (m, 1H), 3.25 (t, *J* = 7.0 Hz, 2H), 1.59 (app p, *J* = 6.9 Hz, 2H), 1.44 (s, 9H), 1.42–1.33 (m, 4H), 1.31–1.23 (m, 14H), 1.07 (d, *J* = 6.8 Hz, 3H).^13^C{^1^H} NMR (101 MHz, CDCl_3_) δ (ppm) 155.9, 79.3, 74.3, 51.5, 50.5, 33.5, 29.7, 29.56, 29.54, 29.52, 29.47, 29.1, 28.8, 28.4, 26.7, 26.1, 14.2. HRMS calcd for C_19_H_39_N_4_O_3_^+^ [M + H]^+^: 371.3022, found 371.3013. [α]_D_^20^ = −4.2 (*c* = 1, CHCl_3_).

#### (2*S*,3*R*,*E*)-2-Amino-14-azidotetradec-4-en-3-ol (Compound **6**)

Acetyl chloride (0.127 mL, 1.86 mmol) was added dropwise to a solution of compound **4** (0.230 g, 0.62 mmol) in MeOH (10 mL) at 0 °C. The reaction mixture was allowed to warm to rt and further stirred at the same temperature for 24 h. The solvent was removed under reduced pressure, and the resulting crude was purified by flash silica gel column chromatography (from 0 to 25% MeOH in CH_2_Cl_2_) to afford compound **6** as a white solid (148 mg, 88% yield). ^1^H NMR (400 MHz, CD_3_OD) δ (ppm) 5.84 (dtd, *J* = 15.2, 6.8, 1.2 Hz, 1H), 5.46 (ddt, *J* = 15.3, 6.6, 1.5 Hz, 1H), 4.22–4.16 (m, 1H), 3.29–3.24 (m, 3H), 2.11 (q, *J* = 7.1 Hz, 2H), 1.64–1.53 (m, 2H), 1.46–1.30 (m, 12H), 1.21 (d, *J* = 6.8 Hz, 3H). ^13^C{^1^H} NMR (101 MHz, CD_3_OD) δ (ppm) 136.4, 128.4, 73.0, 52.8, 52.4, 33.4, 30.6, 30.5, 30.2, 29.9, 27.8, 13.4. HRMS calcd for C_14_H_29_N_4_O^+^ [M + H]^+^: 269.2336, found 269.2328. [α]_D_^20^ = −3.3 (*c* = 1, CHCl_3_).

#### (2*S*,3*R*)-2-Amino-14-azidotetradecan-3-ol (Compound **7**)

Compound **7** was prepared following the same procedure as described for **6** starting from **5** (100 mg, 0.25 mmol) and acetyl chloride (52 μL, 0.73 mmol) in MeOH (5 mL). After purification by flash silica gel column chromatography (from 0 to 25% MeOH in CH_2_Cl_2_), compound **7** was obtained as a white solid (67 mg, 90% yield). ^1^H NMR (400 MHz, CD_3_OD) δ (ppm) 3.70 (td, *J* = 7.7, 7.2, 3.0 Hz, 1H), 3.32–3.24 (m, 3H), 1.58 (dt, *J* = 14.3, 6.8 Hz, 2H), 1.49–1.39 (m, 2H), 1.37–1.30 (m, 16H), 1.21 (d, *J* = 6.8 Hz, 3H).^13^C{^1^H} NMR (101 MHz, CD_3_OD) δ (ppm) 71.6, 52.6, 52.4, 34.0, 30.65, 30.64, 30.62, 30.60, 30.2, 29.9, 27.8, 26.9, 12.1. HRMS calcd for C_14_H_31_N_4_O^+^ [M + H]^+^: 271.2498, found 271.2488. [α]_D_^20^ = +0.5 (*c* = 1, CHCl_3_)

#### *N*-((2*S*,3*R*,*E*)-14-Azido-3-hydroxytetradec-4-en-2-yl)octanamide (Compound **8**)

To a mixture of HOBt (54 mg, 0.405 mmol) and octanoic acid (46 μL, 0.297 mmol) in anhydrous CH_2_Cl_2_ (5 mL) were sequentially added 62 mg (0.405 mmol) of EDC·HCl. Triethylamine (112 μL, 0.81 mmol) was added to a solution of compound **6** (75 mg, 0.27 mmol) in CH_2_Cl_2_ (5 mL). Then, the first solution was added dropwise to the second and the resulting mixture was stirred for 2 h at rt under argon atmosphere. The reaction mixture was diluted with CH_2_Cl_2_ (50 mL) and washed with water (3 × 10 mL). The organic phase was dried over anhydrous MgSO_4_, filtered, and evaporated to dryness under reduced pressure. The crude was purified by flash silica gel column chromatography (from 0 to 3% MeOH in CH_2_Cl_2_) to obtain compound **8** as an off-white waxy solid (148 mg, 72% yield). ^1^H NMR (400 MHz, CDCl_3_) δ (ppm) 5.76–5.66 (m, 1H), 5.56 (d, *J* = 7.7 Hz, 1H), 5.42 (ddt, *J* = 15.4, 6.2, 1.4 Hz, 1H), 4.15–4.06 (m, 2H), 3.26 (t, *J* = 6.9 Hz, 2H), 2.20–2.15 (m, 2H), 2.04 (q, *J* = 7.1 Hz, 2H), 1.66–1.54 (m, 4H), 1.36–1.23 (m, 20H), 1.10 (d, *J* = 6.8 Hz, 3H), 0.90–0.83 (m, 3H). ^13^C{^1^H} NMR (101 MHz, CDCl_3_) δ (ppm) 174.0, 134.0, 128.3, 75.6, 51.6, 50.2, 36.9, 32.5, 31.8, 29.51, 29.45, 29.31, 29.27, 29.24, 29.23, 29.1, 28.9, 26.8, 25.9, 22.7, 15.3, 14.2. HRMS calcd for C_22_H_43_N_4_O_2_^+^ [M + H]^+^: 395.3386, found: 395.3379. [α]_D_^20^ = −6.7 (*c* = 1, CHCl_3_).

#### *N*-((2*S*,3*R*)-14-Azido-3-hydroxytetradecan-2-yl)octanamide (Compound **9**)

Compound **9** was prepared following the same procedure as described for **8**, starting from compound **7** (50 mg, 0.162 mmol), octanoic acid (28 μL, 0.18 mmol), EDC·HCl (47 mg, 0.24 mmol), and HOBt (33 mg, 0.24 mmol) in anhydrous CH_2_Cl_2_ (4 mL) containing NEt_3_ (68 μL, 0.49 mmol). After purification by flash silica gel column chromatography (from 0 to 4% MeOH in CH_2_Cl_2_), compound **9** was obtained as an off-white wax (45 mg, 70% yield). ^1^H NMR (400 MHz, CDCl_3_) δ (ppm) 5.85 (d, *J* = 7.9 Hz, 1H), 4.07–3.91 (m, 1H), 3.61 (br s, 1H), 3.25 (t, *J* = 7.0 Hz, 2H), 2.60 (br s, 1H), 2.22–2.08 (m, 2H), 1.67–1.53 (m, 4H), 1.49–1.20 (m, 26H), 1.08 (d, *J* = 6.9 Hz, 3H), 0.93–0.83 (m, 3H). ^13^C{^1^H} NMR (101 MHz, CDCl_3_) δ (ppm) 173.4, 74.4, 51.6, 49.6, 37.0, 33.7, 31.8, 29.7, 29.65, 29.63, 29.60, 29.56, 29.3, 29.2, 29.1, 28.9, 26.8, 26.1, 25.9, 22.7, 14.8. HRMS calcd for C_22_H_45_N_4_O_2_^+^ [M + H]^+^: 397.3543, found 397.3542. [α]_D_^20^ = −13.6 (*c* = 1, CHCl_3_).

#### *N*-((2*S*,3*R*)-14-Azido-1,3-dihydroxytetradecan-2-yl)octanamide (Compound **11**)

Compound **11** was prepared following the same procedure as described for **8**, starting from the corresponding aminodiol^35^ (45 mg, 0.157 mmol), octanoic acid (27 μL, 0.17 mmol), EDC·HCl (45 mg, 0.23 mmol), and HOBt (32 mg, 0.23 mmol) in anhydrous CH_2_Cl_2_ (4 mL) containing NEt_3_ (55 μL, 0.47 mmol). After purification by flash silica gel column chromatography (from 0 to 4% MeOH in CH_2_Cl_2_), compound **11** was obtained as an off-white wax (54 mg, 83% yield). ^1^H NMR (400 MHz, CDCl_3_) δ (ppm) 6.44 (d, *J* = 7.8 Hz, 1H), 3.98 (d, *J* = 11.3 Hz, 1H), 3.86–3.77 (m, 1H), 3.77–3.70 (m, 2H), 3.25 (t, *J* = 7.0 Hz, 2H), 3.18–3.10 (m, 1H), 3.00 (d, *J* = 6.4 Hz, 1H), 2.22 (t, *J* = 7.6 Hz, 2H), 1.69–1.43 (m, 6H), 1.38–1.20 (m, 24H), 0.87 (t, *J* = 6.7 Hz, 3H). ^13^C{^1^H} NMR (101 MHz, CDCl_3_) δ (ppm) 173.9, 74.1, 62.5, 53.9, 51.6, 37.0, 34.6, 31.8, 29.70, 29.67, 29.65, 29.62, 29.58, 29.4, 29.3, 29.1, 29.0, 26.8, 26.1, 25.9, 22.7, 14.2. HRMS calcd for C_22_H_45_N_4_O_3_^+^ [M + H]^+^: 413.3492, found 413.3483. [α]_D_^20^ = −4.3 (*c* = 1, CHCl_3_).

#### 1-DeoxyCer-COUPY Conjugate **COUPY-3**

A solution of sodium ascorbate (1.25 g, 6.33 mmol) in 50 mL of *tert*-butanol/water (4:1, v/v) was combined with a suspension of CuSO_4_ (1.03 g, 6.33 mmol) in 50 mL of *tert*-butanol/water (4:1, v/v). The resulting dark orange suspension was immediately added to a round-bottom flask containing azide **8** (97 mg, 0.245 mmol). The previous mixture was then transferred to another round-bottom flask containing alkyne **12**(26) (160 mg, 0.316 mmol), and the resulting suspension was stirred overnight at room temperature under argon atmosphere. The solvent was removed by rotary evaporation, and the residue was taken up in CH_2_Cl_2_ (100 mL) and water (100 mL). The organic layer was washed with brine, 10% aq NaHCO_3_ (50 mL), and 10% aq NH_4_Cl (50 mL), dried over anhydrous MgSO_4_, filtered, and concentrated *in vacuo*. The crude was purified by flash silica gel column chromatography (from 0 to 21% MeOH in CH_2_Cl_2_) to furnish compound **COUPY-3** as a dark purple solid (23 mg, 10% yield). ^1^H NMR (400 MHz, CDCl_3_) δ (ppm) 9.18 (s, 1H), 8.63 (d, *J* = 6.7 Hz, 2H), 8.06 (d, *J* = 6.3 Hz, 2H), 7.95 (s, 1H), 7.52 (d, *J* = 9.1 Hz, 1H), 6.88 (s, 1H), 6.83–6.75 (m, 2H), 6.07 (d, *J* = 7.7 Hz, 1H), 5.68 (dt, *J* = 14.4, 6.9 Hz, 1H), 5.60 (s, 2H), 5.41 (dd, *J* = 15.4, 6.4 Hz, 1H), 4.59 (d, *J* = 5.4 Hz, 2H), 4.29 (t, *J* = 7.3 Hz, 2H), 4.12 (d, *J* = 6.5 Hz, 1H), 4.05 (q, *J* = 8.4, 8.0 Hz, 1H), 3.55 (q, *J* = 7.2 Hz, 4H), 2.49 (s, 3H), 2.18 (t, *J* = 7.7 Hz, 2H), 1.99 (q, *J* = 7.2 Hz, 2H), 1.86 (t, *J* = 7.0 Hz, 2H), 1.65–1.50 (m, 2H), 1.34–1.16 (m, 26H), 1.07 (d, *J* = 6.8 Hz, 3H), 0.84 (t, *J* = 6.7 Hz, 3H). ^13^C{^1^H} NMR (101 MHz, CDCl_3_) δ (ppm) 174.0, 167.4, 164.9, 155.5, 152.3, 150.3, 143.3, 133.7, 128.6, 126.6, 123.1, 120.5, 118.1, 112.2, 111.6, 97.0, 79.7, 75.6, 60.3, 50.6, 50.3, 45.4, 36.9, 35.7, 32.4, 31.8, 30.2, 29.8, 29.3, 29.19, 29.15, 29.12, 29.0, 28.8, 26.4, 25.9, 22.7, 19.1, 15.2, 14.2, 12.7. HRMS calcd for C_48_H_69_N_8_O_4_^+^ [M]^+^: 821.5436, found 821.5437.

#### 1-DeoxydhCer-COUPY Conjugate **COUPY-4**

Compound **COUPY-4** was prepared following the same procedure as described for **COUPY-3** starting from azide **9** (30 mg, 75 μmol), alkyne **12** (50 mg, 98 μmol), sodium ascorbate (375 mg, 1.89 mmol), and CuSO_4_ (302 mg, 1.89 mmol) in 4:1 (v/v) ^t^BuOH/H_2_O (30 mL). After purification by flash silica gel column chromatography (from 0 to 21% MeOH in CH_2_Cl_2_), compound **COUPY-4** was obtained as a dark purple solid (22 mg, 32% yield). ^1^H NMR (400 MHz, CD_3_OD) δ (ppm) 8.49–8.36 (m, 2H), 8.13–8.03 (m, 2H), 7.65 (d, *J* = 9.1 Hz, 1H), 7.00–6.92 (m, 1H), 6.91 (s, 1H), 6.81 (s, 1H), 5.27 (s, 2H), 4.57 (s, 2H), 4.40 (t, *J* = 7.0 Hz, 2H), 3.82 (app p, *J* = 6.7 Hz, 1H), 3.58 (q, *J* = 7.1 Hz, 4H), 3.48–3.39 (m, 1H), 2.47 (s, 3H), 2.17 (t, *J* = 7.6 Hz, 2H), 1.95–1.83 (m, 2H), 1.64–1.55 (m, 2H), 1.50–1.20 (m, 32H), 1.09 (d, *J* = 6.8 Hz, 3H), 0.89 (t, *J* = 6.5 Hz, 3H). ^13^C{^1^H} NMR (101 MHz, CD_3_OD) δ (ppm) 175.6, 168.8, 166.9, 156.8, 154.7, 154.1, 151.6, 144.9, 128.1, 121.2, 119.0, 113.6, 112.1, 111.9, 97.5, 80.1, 75.0, 60.9, 51.5, 50.6, 46.0, 37.2, 36.0, 34.8, 32.9, 31.3, 30.8, 30.73, 30.70, 30.66, 30.58, 30.3, 30.2, 30.1, 27.5, 27.1, 27.0, 23.7, 19.1, 15.4, 14.4, 12.9. HRMS calcd for C_48_H_71_N_8_O_4_ [M + H]^+^: 823.5593, found 823.5610.

#### Cer-COUPY Conjugate **COUPY-1**

Compound **COUPY-1** was prepared following the same procedure as described for **COUPY-3** starting from azide **10**(35) (15 mg, 36.5 μmol), alkyne **12** (24 mg, 47.5 μmol), sodium ascorbate (181 mg, 913.3 μmol), and CuSO_4_ (146 mg, 913.3 μmol) in 4:1 (v/v) *tert*-butanol/water (15 mL). After purification by flash silica gel column chromatography (from 0 to 21% MeOH in CH_2_Cl_2_), compound **COUPY-1** was obtained as a dark purple solid (6 mg, 15% yield). ^1^H NMR (400 MHz, CD_3_OD) δ (ppm) 8.41 (d, *J* = 6.9 Hz, 2H), 8.00 (d, *J* = 6.7 Hz, 3H), 7.60 (d, *J* = 9.1 Hz, 1H), 6.92 (dd, *J* = 9.2, 2.5 Hz, 1H), 6.86 (d, *J* = 2.5 Hz, 1H), 6.71 (s, 1H), 5.68 (dt, *J* = 15.4, 6.7 Hz, 1H), 5.45 (ddt, *J* = 15.3, 7.4, 1.5 Hz, 1H), 5.27 (s, 2H), 4.57 (s, 2H), 4.39 (t, *J* = 7.1 Hz, 2H), 4.06 (t, *J* = 7.2 Hz, 1H), 3.90–3.80 (m, 1H), 3.73–3.62 (m, 2H), 3.57 (q, *J* = 7.1 Hz, 4H), 2.41 (s, 3H), 2.23–2.15 (m, 2H), 2.01 (q, *J* = 6.7 Hz, 2H), 1.89 (app p, *J* = 7.0 Hz, 2H), 1.58 (app p, *J* = 7.1 Hz, 2H), 1.36–1.20 (m, 26H), 0.91–0.86 (m, 3H). ^13^C{^1^H} NMR (101 MHz, CD_3_OD) δ (ppm) 176.3, 168.6, 166.9, 156.7, 154.6, 154.1, 151.4, 144.9, 134.5, 131.3, 128.1, 124.4, 121.1, 118.9, 113.6, 112.0, 111.8, 97.4, 80.1, 73.6, 62.3, 60.9, 56.7, 51.4, 46.0, 37.3, 35.9, 33.4, 32.9, 31.3, 30.5, 30.37, 30.31, 30.25, 30.15, 30.11, 30.0, 27.5, 27.1, 23.7, 19.1, 14.5, 13.0. HRMS calcd for C_48_H_69_N_8_O_5_^+^ [M]^+^: 837.5385, found 837.5392.

#### dhCer-COUPY Conjugate **COUPY-2**

Compound **COUPY-2** was prepared following the same procedure as described for **COUPY-3** starting from azide **11** (15 mg, 36.4 μmol), alkyne **12** (24 mg, 47.3 μmol), sodium ascorbate (180 mg, 908.9 μmol), and CuSO_4_ (145 mg, 908.9 μmol) in 4:1 (v/v) *tert*-butanol/water (15 mL). After purification by flash silica gel column chromatography (from 0 to 21% MeOH in CH_2_Cl_2_), compound **COUPY-2** was obtained as a dark purple solid (7 mg, 21% yield). ^1^H NMR (400 MHz, CD_3_OD) δ (ppm) 8.41 (d, *J* = 7.3 Hz, 2H), 8.14 (d, *J* = 7.4 Hz, 2H), 7.94 (s, 1H), 7.70 (d, *J* = 9.2 Hz, 1H), 6.98 (dd, *J* = 9.1, 2.5 Hz, 1H), 6.95 (d, *J* = 2.5 Hz, 1H), 6.90 (d, *J* = 1.0 Hz, 1H), 5.23 (s, 2H), 4.55 (s, 2H), 4.39 (t, *J* = 7.1 Hz, 2H), 3.87–3.76 (m, 1H), 3.71–3.67 (m, 2H), 3.60 (q, *J* = 7.1 Hz, 5H), 2.52 (d, *J* = 0.9 Hz, 3H), 2.22 (t, *J* = 7.5 Hz, 2H), 1.89 (app p, *J* = 6.8 Hz, 2H), 1.67–1.55 (m, 2H), 1.39–1.21 (m, 32H), 0.93–0.85 (m, 3H). ^13^C{^1^H} NMR (101 MHz, CD_3_OD) δ (ppm) ^13^C NMR (101 MHz, MeOD) δ 176.3, 169.0, 166.9, 156.9, 154.8, 154.1, 151.8, 144.8, 128.1, 124.3, 121.2, 119.1, 113.5, 112.2, 112.0, 97.5, 80.0, 72.4, 62.4, 59.9, 56.7, 51.4, 45.9, 37.3, 35.9, 35.0, 32.9, 31.3, 30.8, 30.7, 30.63, 30.56, 30.3, 30.2, 30.1, 27.5, 27.1, 26.7, 23.7, 19.0, 14.5, 12.8. HRMS calcd for C_48_H_71_N_8_O_5_^+^ [M]^+^: 839.5542, found 839.5536.

### Synthesis and Characterization of BODIPY-Labeled Ceramides and 1-Deoxyceramides

#### (2*S*,3*R*,*E*)-*tert*-Butyl(14-(4,4-difluoro-1,3,5,7-tetramethyl-4-bora-3a,4a-diaza-*s*-indacene-8-yl)-3-hydroxytetradec-5-en-2-yl)carbamate (Compound **15**)

Compound **15** was prepared following the same procedure as described for **3**, starting from homoallylic alcohol **13**(34) (430 mg, 2.00 mmol), BODIPY-alkene **14**(37) (579 mg, 1.50 mmol), and second-generation Grubbs’ catalyst (42 mg, 0.05 mmol) in CH_2_Cl_2_ (20 mL). After purification by flash silica gel column chromatography (from 0 to 30% EtOAc in hexanes), compound **15** was obtained as an orange wax (344 mg, 40% yield). ^1^H NMR (400 MHz, CDCl_3_) δ (ppm) 6.03 (s, 2H), 5.52 (dd, *J* = 14.2, 7.5 Hz, 1H), 5.46–5.33 (m, 1H), 4.81 (br s, 1H), 3.74–3.54 (m, 2H), 2.99–2.83 (m, 2H), 2.50 (s, 6H), 2.39 (s, 6H), 2.23–1.90 (m, 5H), 1.67–1.54 (m, 2H), 1.51–1.45 (m, 2H), 1.43 (s, 9H), 1.38–1.21 (m, 8H), 1.09 (d, *J* = 6.7 Hz, 3H). ^13^C{^1^H} NMR (101 MHz, CDCl_3_) δ (ppm) 155.8, 153.8, 146.8, 140.4, 134.6, 131.5, 125.8, 121.7, 79.5, 73.6, 50.2, 37.3, 32.7, 32.0, 30.5, 29.52, 29.47, 29.2, 28.58, 28.52, 16.5, 14.6, 14.5. HRMS calcd for C_32_H_50_BF_2_N_3_NaO_3_^+^ [M + Na]^+^: 596.3811, found 596.3806.

#### (2*S*,3*R*)-*tert*-Butyl(14-(4,4-difluoro-1,3,5,7-tetramethyl-4-bora-3a,4a-diaza-*s*-indacene-8-yl)-3-hydroxytetradecan-2-yl)carbamate (Compound **16**)

A solution of **15** (300 mg, 0.52 mmol) in degassed EtOAc (15 mL) was hydrogenated at 1 atm and rt in the presence of Rh/Al_2_O_3_ (15 mg, 5% w/w). After stirring for 48 h, the catalyst was removed by filtration through a bed of Celite, and the solid pad was rinsed with EtOAc (3 × 10 mL). The combined filtrates were concentrated in vacuo, and the residue was subjected to flash chromatography on silica gel (stepwise gradient from 0 to 30% EtOAc in hexanes) to afford **16** as an orange wax (292 mg, 97% yield). ^1^H NMR (400 MHz, CDCl_3_) δ (ppm) 6.04 (s, 2H), 4.76 (br s, 1H), 3.75–3.54 (m, 2H), 2.97–2.88 (m, 2H), 2.51 (s, 6H), 2.41 (s, 6H), 2.16 (br s, 1H), 1.66–1.56 (m, 2H), 1.54–1.46 (m, 2H), 1.44 (s, 9H), 1.35–1.21 (m, 16H), 1.07 (d, *J* = 6.8 Hz, 3H). ^13^C{^1^H} NMR (101 MHz, CDCl_3_) δ (ppm) 156.0, 153.7, 146.8, 140.4, 131.5, 121.6, 79.5, 74.5, 50.6, 33.6, 32.0, 30.5, 29.7, 29.64, 29.62, 29.57, 29.50, 28.6, 28.5, 26.1, 16.4, 14.5, 14.4. HRMS calcd for C_32_H_52_BF_2_N_3_NaO_3_^+^ [M + Na]^+^: 598.3967, found 598.3965.

#### (2*S*,3*R*)-2-Amino-14-(4,4-difluoro-1,3,5,7-tetramethyl-4-bora-3a,4a-diaza-*s*-indacene-8-yl)tetradecan-3-ol (Compound **17**)

To a solution of compound **16** (170 mg, 0.29 mmol) in anhydrous CH_2_Cl_2_ (10 mL) was added BF_3_·OEt_2_ (250 μL, 2.0 mmol) at 0 °C. The resulting mixture was allowed to warm to rt and stirred for 20 min protected from light. The reaction was quenched by adding sat. aqueous NaHCO_3_ (5 mL). After stirring for 5 min, the organic layer was separated and concentrated under reduced pressure to give the crude as a red-brown oil that was purified by flash column chromatography on silica gel (from 0 to 25% MeOH in CH_2_Cl_2_) to afford compound **17** as an orange wax (113 mg, 83% yield). ^1^H NMR (400 MHz, CDCl_3_) δ (ppm) 6.72 (br s, 2H), 6.03 (s, 2H), 3.90–3.85 (m, 1H), 3.47–3.40 (m, 1H), 2.94–2.86 (m, 2H), 2.49 (s, 6H), 2.38 (s, 6H), 1.93 (br s, 1H), 1.66–1.53 (m, 2H), 1.51–1.38 (m, 2H), 1.31–1.20 (m, 19H). ^13^C{^1^H} NMR (101 MHz, CDCl_3_) δ (ppm) 153.8, 146.7, 140.4, 131.5, 121.7, 70.7, 52.0, 33.0, 32.2, 32.0, 31.4, 30.5, 29.8, 29.72, 29.69, 29.5, 28.5, 26.0, 16.4, 14.5, 11.2. HRMS calcd for C_27_H_44_BFN_3_O [M – F]^+^: 456.3556, found 456.3556.

#### *N*-((2*S*,3*R*)-14-(4,4-Difluoro-1,3,5,7-tetramethyl-4-bora-3a,4a-diaza-*s*-indacene-8-yl)-3-hydroxytetradecan-2-yl)octanamide (**BODIPY-4**)

Compound **BODIPY-4** was prepared following the same procedure as described for **8**, starting from compound **17** (47 mg, 0.10 mmol), octanoic acid (20 μL, 0.125 mmol), EDC·HCl (29 mg, 0.15 mmol), and HOBt (20 mg, 0.15 mmol) in anhydrous CH_2_Cl_2_ (10 mL) containing NEt_3_ (40 μL, 0.30 mmol). After purification by flash silica gel column chromatography (from 0 to 55% EtOAc in hexanes), compound **BODIPY-4** was obtained as an orange wax (36 mg, 64% yield). ^1^H NMR (400 MHz, CDCl_3_) δ (ppm) 6.05 (s, 2H), 5.73 (d, *J* = 7.8 Hz, 1H), 4.07–3.95 (m, 1H), 3.66–3.57 (m, 1H), 2.93 (dd, *J* = 10.2, 6.7 Hz, 2H), 2.51 (s, 6H), 2.41 (s, 6H), 2.16 (t, 2H), 1.67–1.56 (m, 2H), 1.52–1.44 (m, 2H), 1.36–1.22 (m, 26H), 1.09 (d, *J* = 6.9 Hz, 3H), 0.87 (t, *J* = 6.8 Hz, 3H). ^13^C{^1^H} NMR (101 MHz, CDCl_3_) δ (ppm) 173.4, 153.9, 146.9, 140.4, 131.6, 121.7, 74.5, 49.6, 37.0, 33.7, 32.1, 31.8, 30.5, 29.74, 29.67, 29.62, 29.56, 29.4, 29.1, 28.6, 26.1, 25.9, 22.7, 16.5, 14.6, 14.3, 14.2. ^19^F NMR (376 MHz, CDCl_3_) δ −146.64 (m). HRMS calcd for C_35_H_58_BFN_3_O_2_^+^ [M – F]^+^: 582.4601, found 582.4604.

#### *N*-((2*S*,3*R*,*E*)-13-(4,4-Difluoro-1,3,5,7-tetramethyl-4-bora-3a,4a-diaza-*s*-indacene-8-yl)-1,3-dihydroxytridec-4-en-2-yl)octanamide (**BODIPY-1**)

Compound **BODIPY-1** was prepared following the same procedure as described for compound **8**, starting from compound **19**(37) (40 mg, 0.084 mmol), octanoic acid (15 μL, 0.092 mmol), EDC·HCl (24 mg, 0.126 mmol), and HOBt (17 mg, 0.126 mmol) in anhydrous CH_2_Cl_2_ (10 mL) containing NEt_3_ (35 μL, 0.25 mmol). After purification by flash silica gel column chromatography (from 0 to 65% EtOAc in hexanes), compound **BODIPY-1** was obtained as an orange wax (32 mg, 63% yield). ^1^H NMR (400 MHz, CDCl_3_) δ 6.24 (d, *J* = 7.0 Hz, 1H), 6.05 (s, 2H), 5.78 (dt, *J* = 14.2, 6.8 Hz, 1H), 5.54 (dd, *J* = 15.2, 6.3 Hz, 1H), 4.45–4.20 (m, 1H), 4.05–3.84 (m, 2H), 3.71 (d, *J* = 9.0 Hz, 1H), 3.00–2.87 (m, 2H), 2.70–2.57 (m, 2H), 2.51 (s, 6H), 2.42 (s, 6H), 2.23 (t, *J* = 7.6 Hz, 2H), 2.06 (q, *J* = 6.7 Hz, 2H), 1.69–1.61 (m, 4H), 1.51–1.43 (m, 2H), 1.40–1.21 (m, 18H), 0.88 (t, *J* = 6.3 Hz, 3H). ^13^C{^1^H} NMR (101 MHz, CDCl_3_) δ (ppm) 174.1, 153.8, 146.8, 140.4, 134.0, 131.5, 129.1, 121.7, 77.5, 77.2, 76.8, 74.6, 62.5, 54.6, 36.9, 32.3, 32.0, 31.8, 30.5, 29.5, 29.5, 29.35, 29.26, 29.21, 29.1, 28.6, 25.9, 25.9, 22.7, 16.5, 14.5, 14.2. ^19^F NMR (376 MHz, CDCl_3_) δ −146.63 (m). HRMS calcd for C_34_H_54_BF_2_N_3_NaO_3_^+^ [M + Na]^+^: 624.4124 found 624.4124.

### Photophysical Characterization of the Compounds

Absorption spectra were recorded on a Jasco V-730 UV–vis spectrophotometer at room temperature. Emission spectra were measured on a Photon Technology International (PTI) Quantamaster fluorimeter. Fluorescence quantum yields (Φ_F_) were measured by a comparative method using Cresyl violet in ethanol (Φ_F_ = 0.54)^39^ as a standard for probes **COUPY-1**, **COUPY-2**, **COUPY-3**, and **COUPY-4**. Fluorescein dissolved in aqueous sodium hydroxide (0.1 M; Φ_F_ = 0.92)^39^ was used as a standard in the case of probes **BODIPY-1** and **BODIPY-4**. Then, optically matched solutions of the probes and the appropriate standard were prepared and fluorescence spectra were recorded. The absorbance of the sample and the standard solutions was set below 0.1 at the excitation wavelength (475 nm for fluorescein and BODIPY-labeled probes and 540 nm for cresyl violet and COUPY-labeled probes) and Φ_F_ was calculated using eq 1
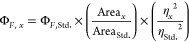
1where Area_*x*_ and Area_Std._ are the integrated fluorescence for the sample and the standard, respectively, and η_*x*_ and η_Std._ are the refractive indices of the sample and the standard solutions, respectively. The uncertainty in the experimental value of Φ_F_ has been estimated to be approximately 10%.

### Cell Culture and Treatments

HeLa cells were maintained in Dulbecco’s modified Eagle’s medium (DMEM) containing high glucose (4.5 g/L) and supplemented with 10% fetal bovine serum (FBS) and 50 U/mL penicillin–streptomycin. For cellular uptake experiments and posterior observation under a microscope, cells were seeded on glass-bottom dishes (P35G-1.5-14-C, Mattek). The cells were incubated for 30 min at 37 °C with COUPY-labeled or BODIPY-labeled probes (1 μM) in supplemented DMEM, 24 h after cell seeding. Then, the cells were washed three times with Dulbecco’s phosphate-buffered saline (DPBS, pH 7.0–7.3) to remove the excess fluorophores and kept in low-glucose DMEM without phenol red for fluorescence imaging.

For colocalization experiments of COUPY-labeled probes with LysoTracker Green and CellMask Deep Red, HeLa cells were treated with COUPY probes (1 μM) in supplemented DMEM. After 30 min incubation at 37 °C, the cells were washed three times with DPBS and CellMask Deep Red (3 μg/mL) was added in nonsupplemented DMEM, and cells were incubated for a further 10 min at 37 °C. Upon removal of the medium and washing three times with DPBS, the cells were incubated for 5 min more at 37 °C with LysoTracker Green (0.2 μM) in nonsupplemented DMEM. Finally, the cells were washed three times with DPBS and kept in low-glucose DMEM without phenol red for fluorescence imaging.

For colocalization experiments of BODIPY-labeled probes with LysoView650, WGA555, and MitoView650, HeLa cells were treated with BODIPY-labeled probes (1 μM) in supplemented DMEM. After 30 min incubation at 37 °C, the cells were washed three times with DPBS and MitoView650 (100 nM) or LysoView650 (1X) was added in nonsupplemented DMEM. After 10 more min of incubation at 37 °C, WGA555 (100 nM) was added and cells were further incubated for 10 min at 37 °C. Upon removal of the medium and washing three times with DPBS, the cells were kept in low-glucose DMEM without phenol red for fluorescence imaging. For those observations after longer incubation times (45 min to 2 h), cells were kept in the incubator at 37 °C.

### Fluorescence Imaging

All microscopy observations were performed using a Zeiss LSM 880 confocal microscope equipped with an argon-ion laser, a 561 nm laser, and a 633 nm laser. The microscope was also equipped with a Heating Insert P S (Pecon) and a 5% CO_2_ providing system. Cells were observed at 37 °C using a 63 × 1.4 oil immersion objective. COUPY-labeled probes were excited using the 561 nm laser and detected from 570 to 670 nm. BODIPY-labeled probes were excited using the 488 nm laser line of the argon-ion laser and detected from 501 to 550 nm. In colocalization studies of the compounds with organelle markers, LysoTracker Green was observed using the 488 nm laser line, CellMask Deep Red, LysoView650, and MitoView650 were observed using the 633 nm laser and WGA555 using the 561 nm laser. Image processing and analysis were performed using Fiji.^43^

### Image Analysis

Intensity measurements of the vesicles observed in the COUPY probe images were performed on the maximum intensity projections (MIP) of the image stacks. After projecting, these MIPs were filtered with a median filter of radius 2 and background-subtracted with a rolling ball of 10.

In the colocalization studies of the COUPY probes with the organelle markers, all stainings were processed identically. First, the stacks of images were filtered with a median and Gauss filters, both with a radius of 1. Then, the background was subtracted from the stacks with a rolling ball of 10. Otsu intensity threshold^44^ was then checked in all stacks to finally apply it to the JaCoP plugin^41^ to analyze the colocalization. The same processing pipeline was followed to analyze the colocalization of BODIPY compounds with the organelle markers although in this case the rolling ball of background subtraction was 50 for the compound images as they showed a more spread staining inside cells.

The colocalization of BODIPY with COUPY probes was performed by first filtering with a median and Gauss filters both with a radius of 1 and subtracting background with a rolling ball of 50. Next, COUPY images were segmented using the auto local threshold Phansalkar^45^ with a radius of 2 and BODIPY images using the auto local threshold^46^ with a radius of 3. Then, binary images obtained after segmentation were used as masks on the original compound images. Masked images were finally analyzed for colocalization using the JaCoP plugin.

## Supporting Information Available

UV–vis absorption and fluorescence emission spectra of fluorescent probes **COUPY-2** and **COUPY-3**, additional fluorescence imaging studies, and copies of ^1^H and ^13^C{^1^H} NMR spectra of the synthesized compounds (PDF)

## Dedication

Dedicated to the memory of Prof. Antonio Delgado Cirilo.
