# Supplementary material for: Fluorescently Labeled Ceramides and 1-Deoxyceramides: Synthesis, Characterization, and Cellular Distribution Studies
Source: J Org Chem. 2022 Nov 28;87(24):16351–67. doi: 10.1021/acs.joc.2c02019 (PMC9764360; doi:10.1021/acs.joc.2c02019)
Supplement: Supplementary file 1 — jo2c02019_si_001.pdf [file jo2c02019_si_001.pdf]

SUPPORTING INFORMATION

Fluorescently-labelled ceramides and 1-deoxyceramides: Synthesis, characterization and cellular distribution studies

Eduardo Izquierdo,[a] Marta López-Corrales,[a] Diego Abad-Montero,[a,b] Anna Rovira,[a] Gemma Fabriàs,[b] Manel Bosch,[c] José Luís Abad[b,*] and Vicente Marchán[a,d,*]

[a]Departament de Química Inorgànica i Orgànica, Secció de Química Orgànica, Universitat de Barcelona (UB). Martí i Franquès 1-11, 08028 Barcelona, Spain.

[b]Research Unit on BioActive Molecules, Departament de Química Biològica, Institut de Química Avançada de Catalunya (IQAC-CSIC). Jordi Girona 18-26, 08034 Barcelona, Spain.

[c]Unitat de Microscòpia Òptica Avançada, Centres Científics i Tecnològics, Universitat de Barcelona (UB). Av. Diagonal, 643, 08028 Barcelona, Spain.

[d]Institut de Biomedicina de la Universitat de Barcelona (IBUB), 08028 Barcelona, Spain.

Table of contents

1. Photophysical characterization of probes **COUPY-2** and **COUPY-3**.................... S3

2. Supplementary data of confocal microscopy studies S4

3. NMR spectra of the synthesized compounds S5

1. Photophysical characterization of probes COUPY-2 and COUPY-3

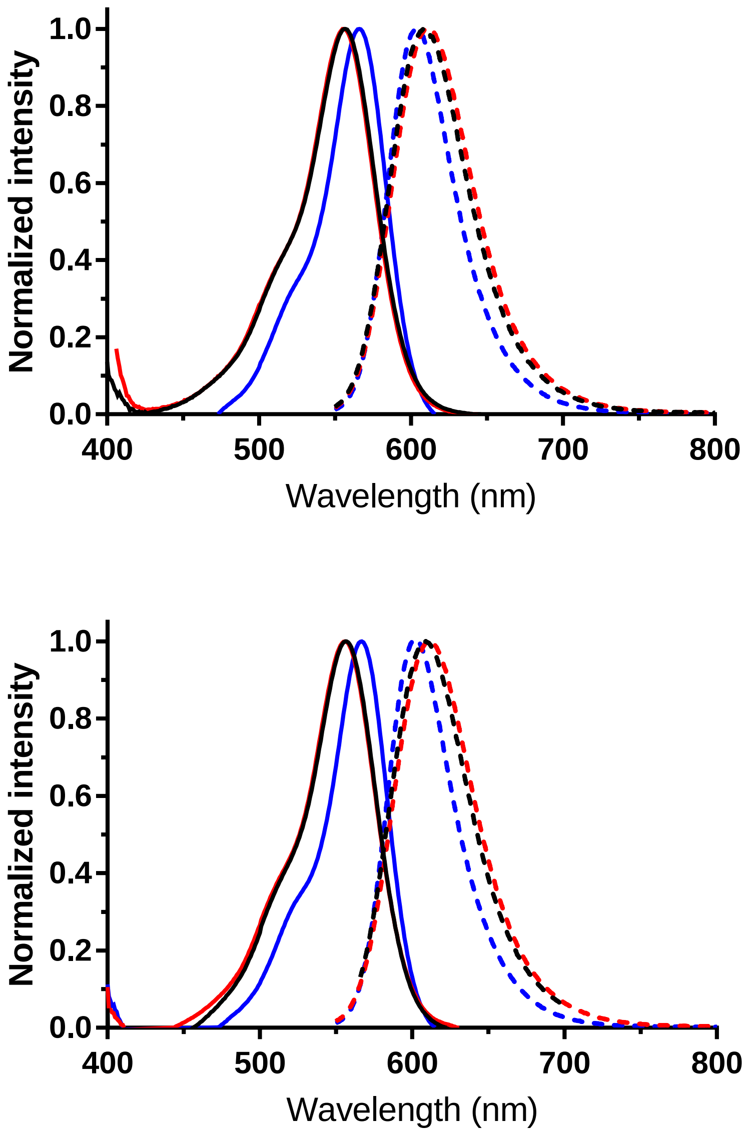

Figure S1. Comparison of the normalized absorption (solid lines) and fluorescence (dotted lines) spectra upon excitation at 540 nm of compounds **COUPY-2** (top) and **COUPY-3** (bottom) in MeOH (black lines), ACN (red lines) and DCM (blue lines).

2. Supplementary data of confocal microscopy studies

Table S1. Colocalization coefficients between compounds and organelle staining. M1 and M2 are Manders' coefficients: compound over organelle staining and organelle staining over compound, respectively. T-student differences are highlighted in yellow ($p<0.05$) and orange ($p<0.01$); n > 15 cells.

	Lysotracker green (LTG)			CellMask		
	Pearson	M1	M2	Pearson	M1	M2
**COUPY-1**	0.47	0.35	0.45	0.21	0.37	0.03
**COUPY-3**	0.51	0.4	0.51	0.31	0.5	0.06
**COUPY-2**	0.49	0.37	0.5	0.29	0.51	0.04
**COUPY-4**	0.47	0.23	0.65	0.28	0.36	0.06

Table S2. **Colocalization coefficients between BODIPY compounds and organelle staining**. Cells were observed just after compound and markers incubations (t0) or after washing and incubating with media for longer periods of time (t45' = 45 min, t120' = 120 min). M1 and M2 are Manders' coefficients: compound over organelle staining and organelle staining over compound, respectively. T-student differences between incubation times for each compound are highlighted in yellow ($p<0.05$) and orange ($p<0.01$); n > 15 cells.

	Lysoview650			WGA555		
	Pearson	M1	M2	Pearson	M1	M2
**BODIPY-1 t0**	0,18	0,04	0,20	0,14	0,32	0,44
**BODIPY-1 t45'**	0,13	0,04	0,16	0,46	0,47	0,72
**BODIPY-4 t0**	0,28	0,06	0,29	0,21	0,26	0,32
**BODIPY-4 t120'**	0,28	0,21	0,49	0,44	0,28	0,77

Table S3. Colocalization coefficients between BODIPY compounds and Mitochondria staining. M1 and M2 are Manders' coefficients: compound over mitochondria and mitochondria staining over compound, respectively; n > 15 cells.

	Mitoview650		
	Pearson	M1	M2
**BODIPY-1**	0,29	0,18	0,62
**BODIPY-4**	0,44	0,21	0,78

3. NMR spectra of the synthesized compounds

Compound 1

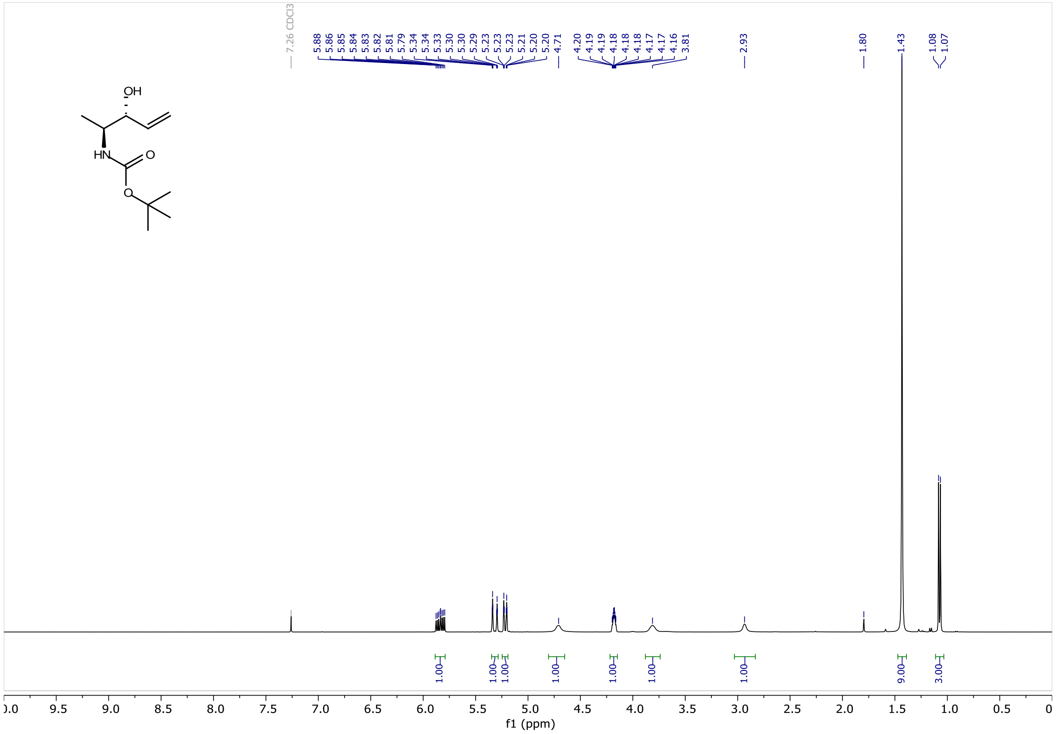

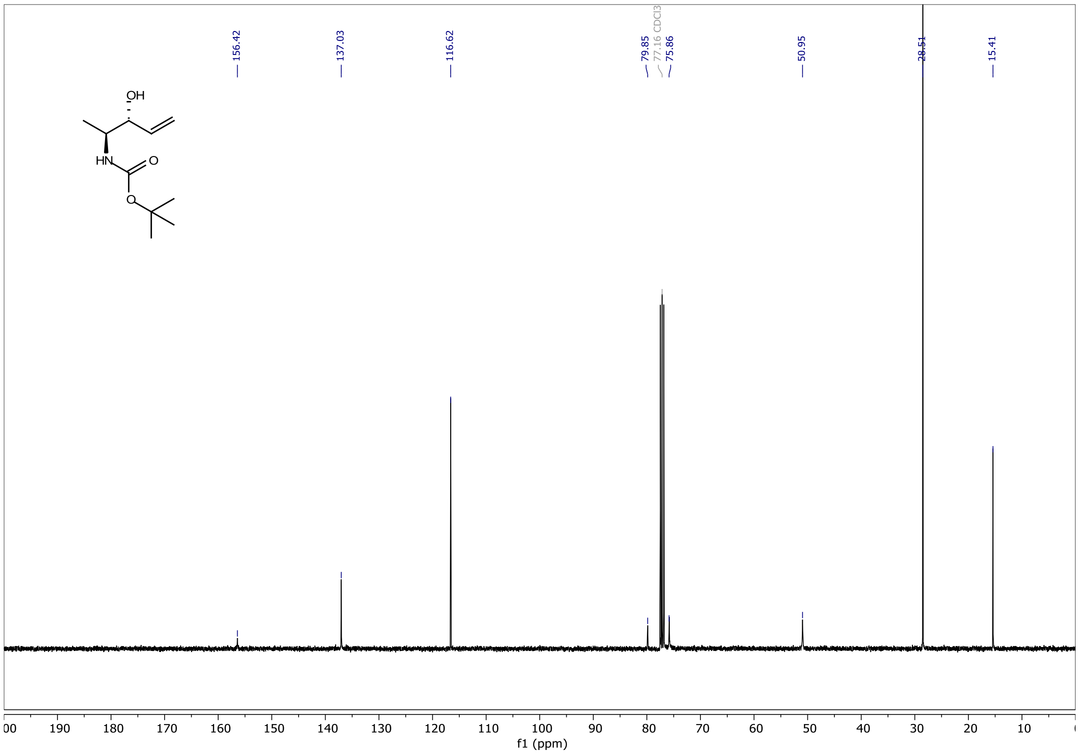

Figure S2. ^{1}H(400 MHz) and ^{13}C{^{1}H}(101 MHz) NMR spectra of compound **1** in $CDCl_3$.

Compound 2

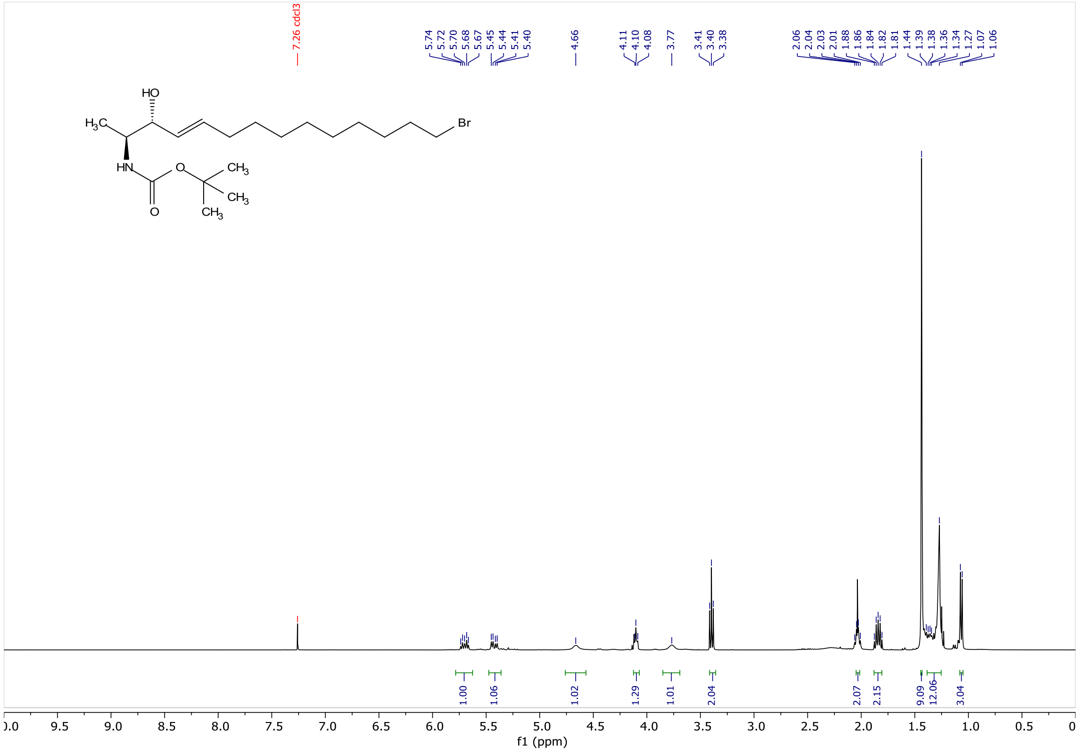

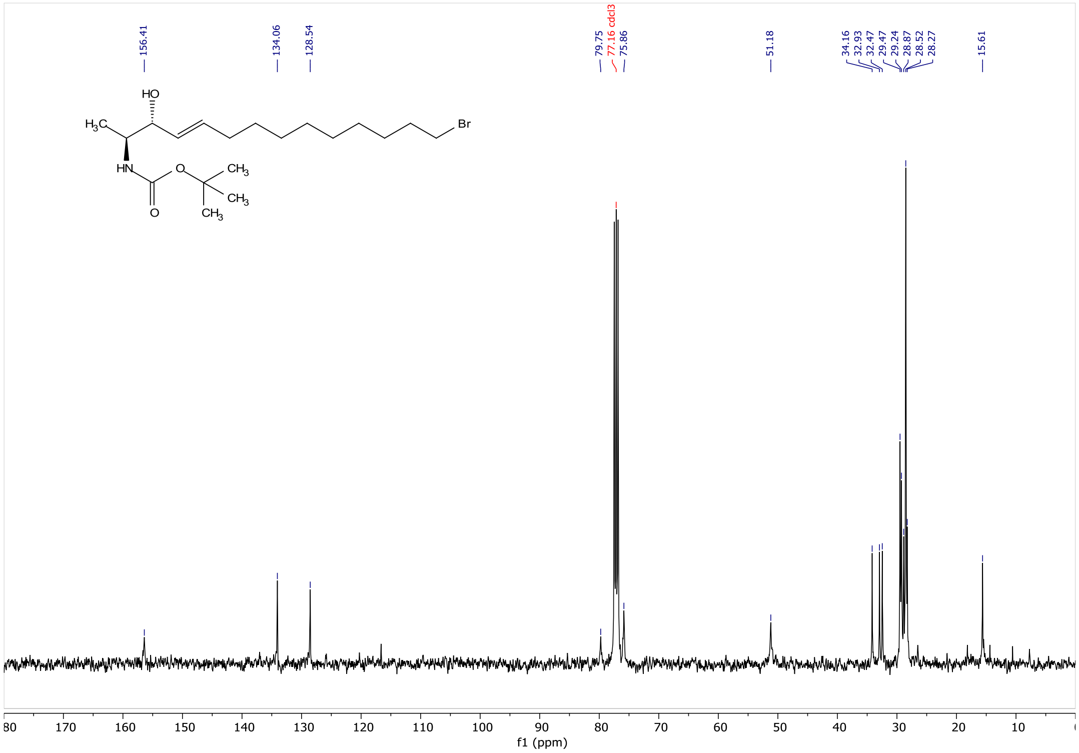

Figure S3. ^{1}H(400 MHz) and ^{13}C{^{1}H}(101 MHz) NMR spectra of compound **2** in $CDCl_3$.

Compound 3

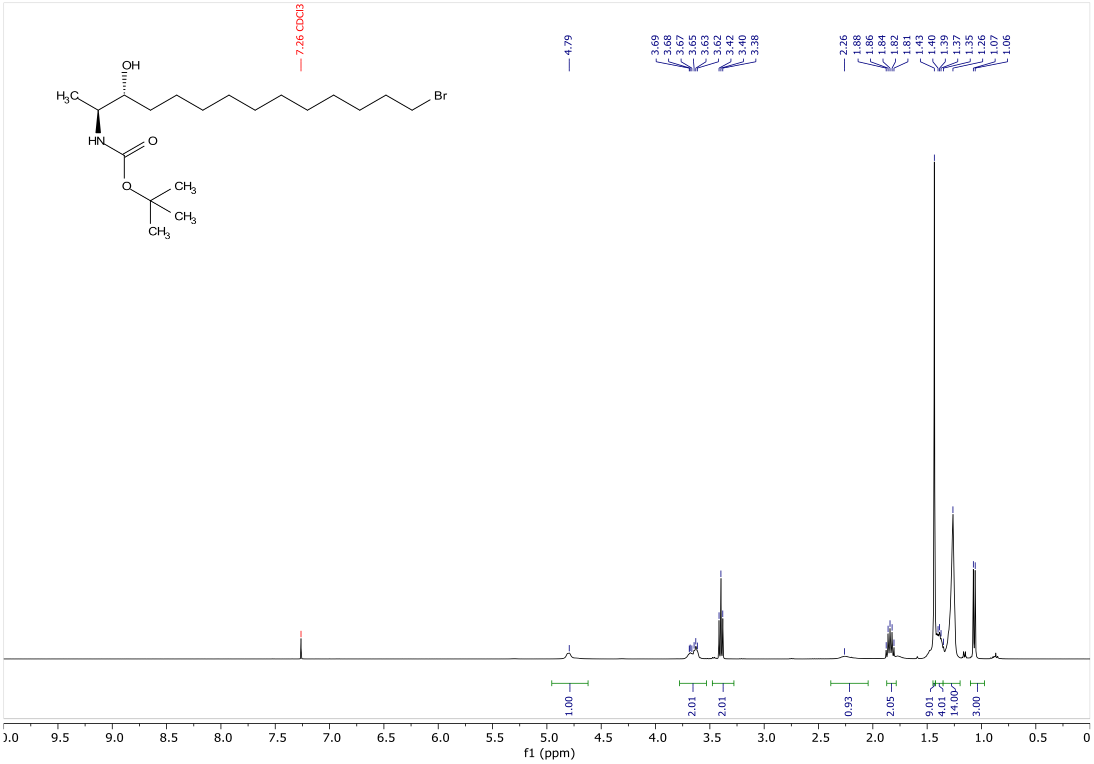

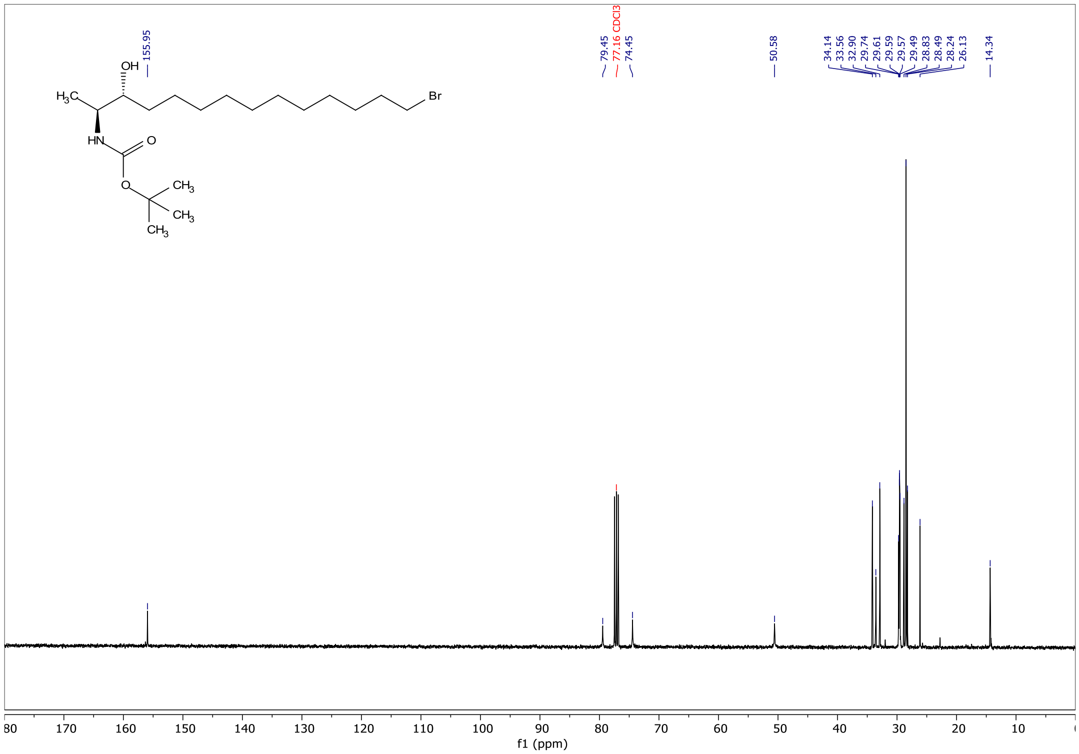

Figure S4. ^{1}H(400 MHz) and ^{13}C{^{1}H}(101 MHz) NMR spectra of compound **3** in $CDCl_3$.

Compound 4

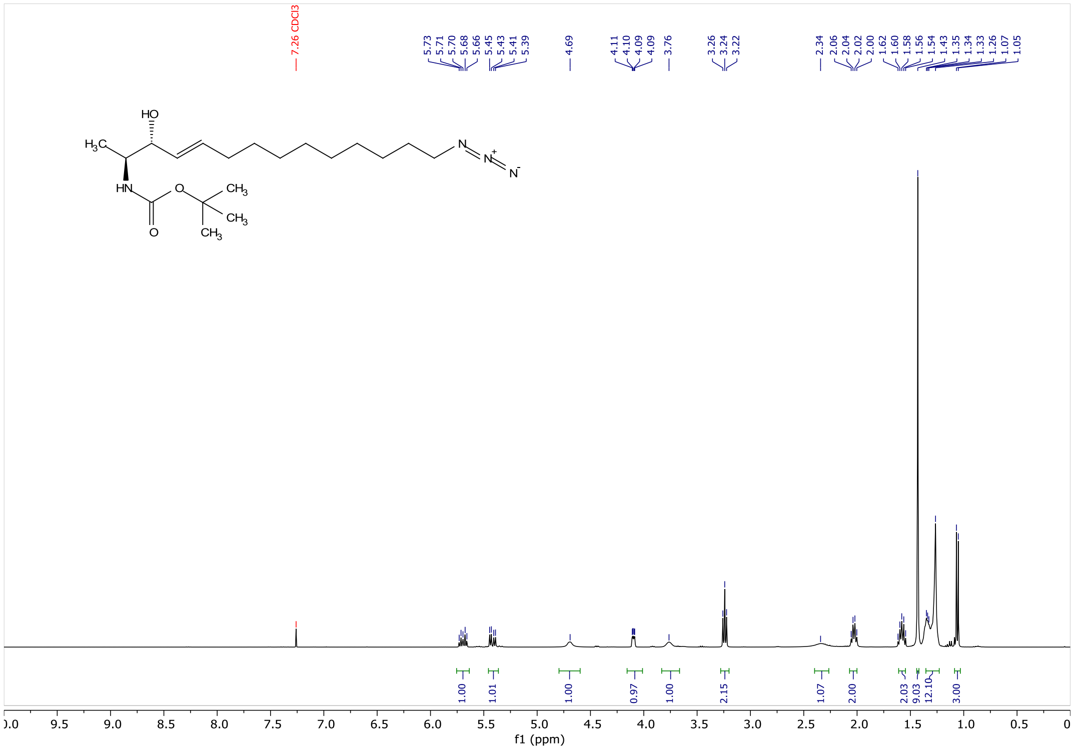

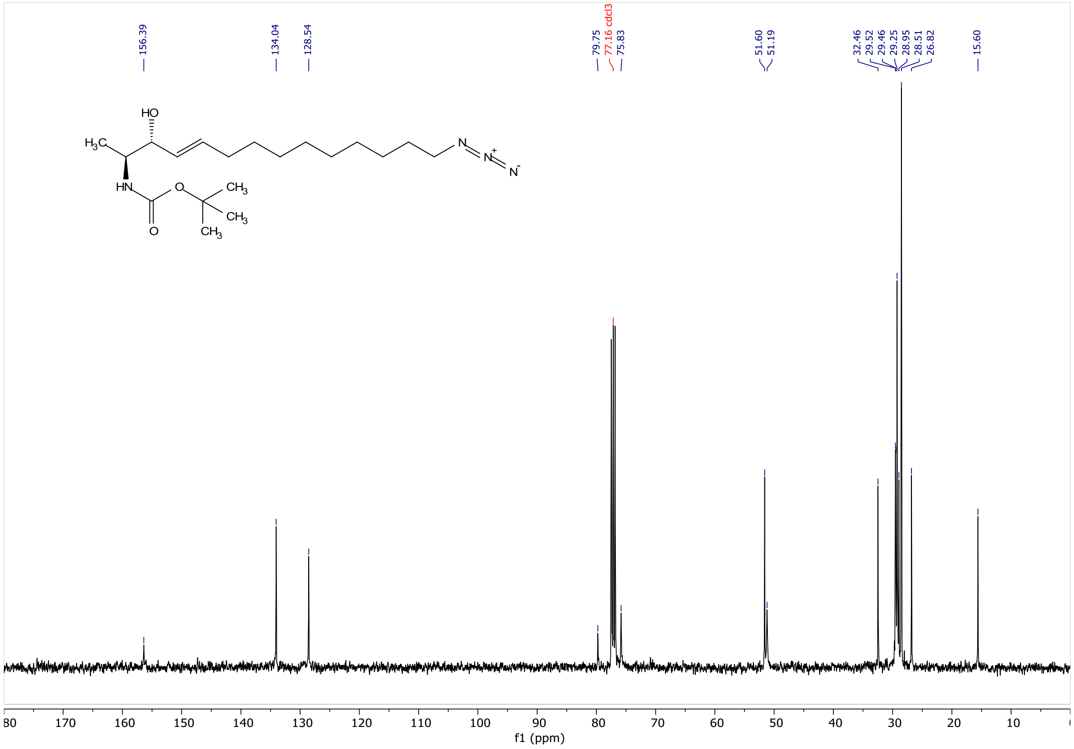

Figure S5. ^{1}H(400 MHz) and $^{13}C\{^{1}H\}$(101 MHz) NMR spectra of compound **4** in $CDCl_3$.

Compound 5

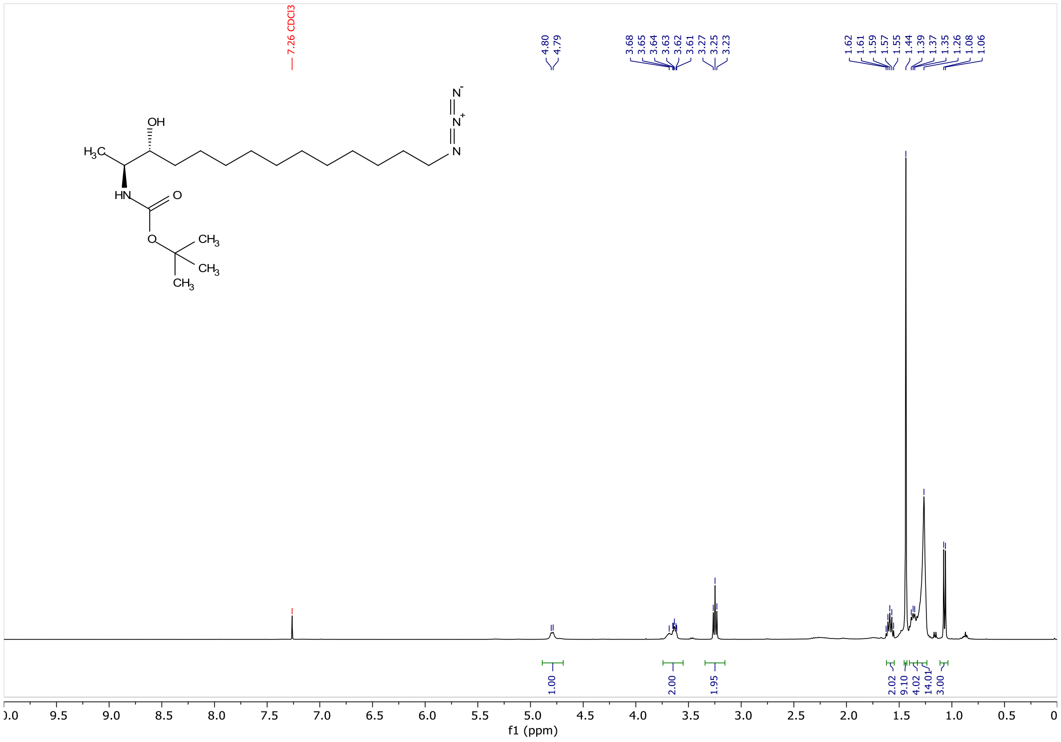

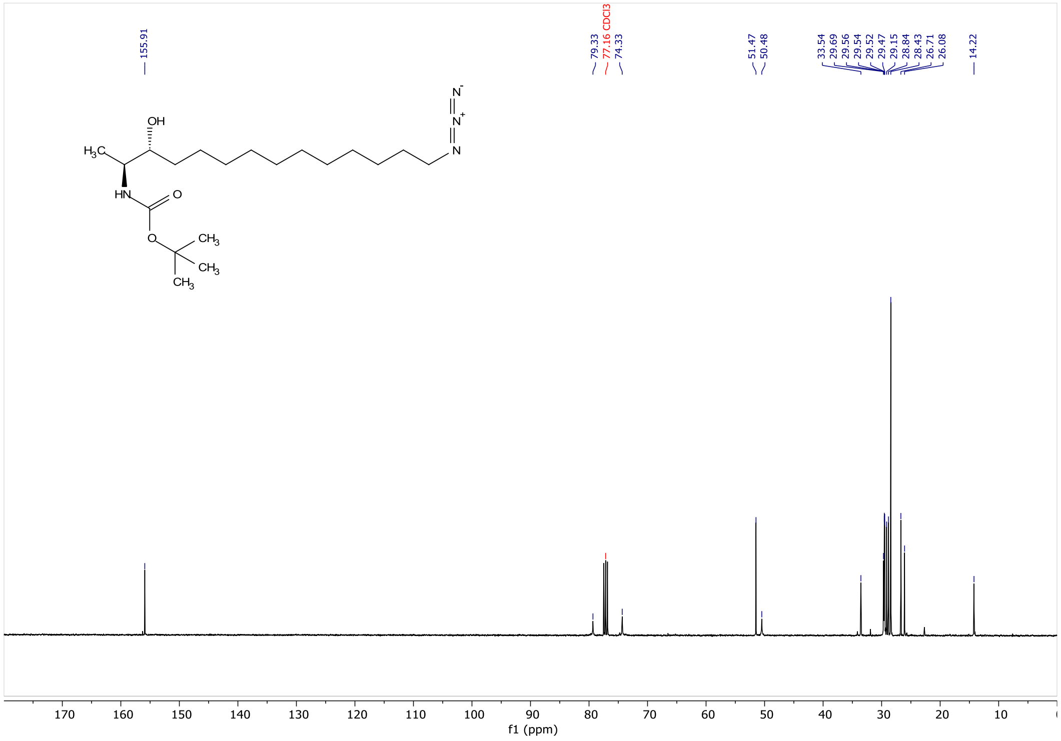

Figure S6. ^{1}H(400 MHz) and $^{13}C\{^{1}H\}$(101 MHz) NMR spectra of compound **5** in $CDCl_3$.

Compound 6

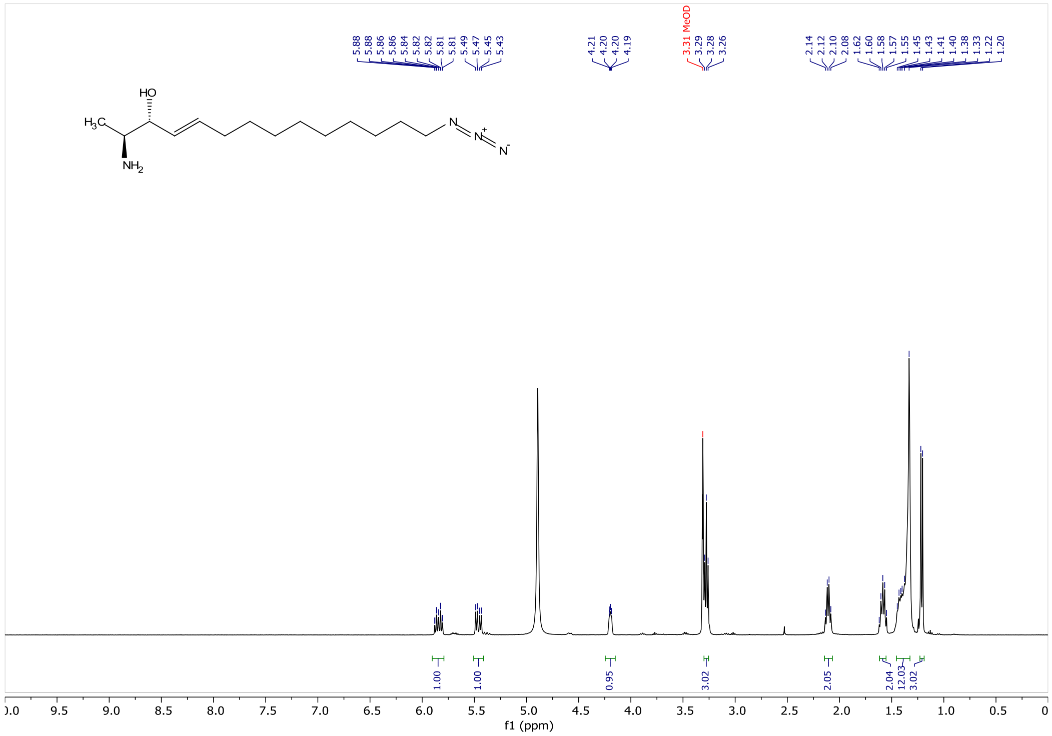

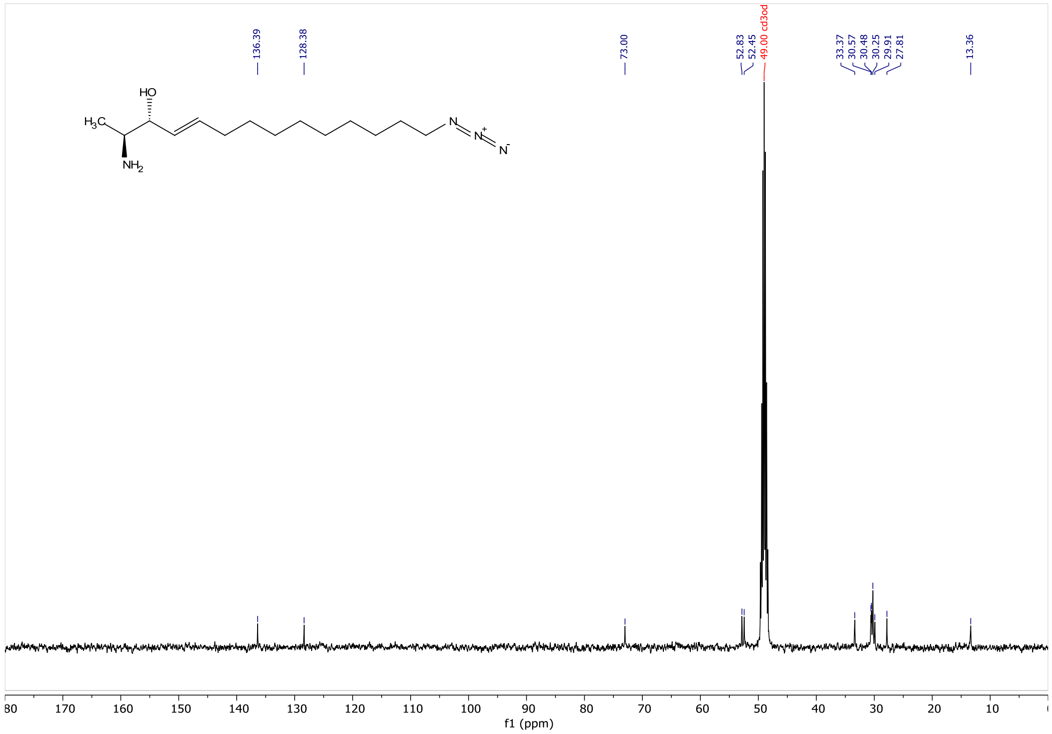

Figure S7. ^{1}H(400 MHz) and $^{13}C\{^{1}H\}$(101 MHz) NMR spectra of compound **6** in CD_3OD.

Compound 7

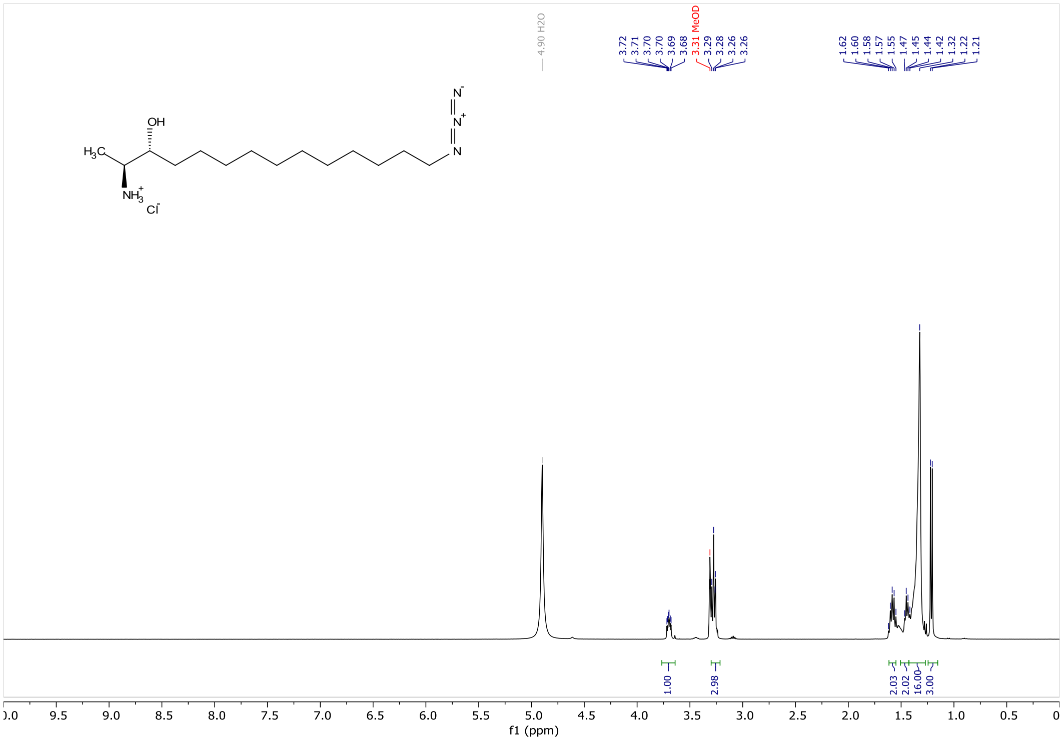

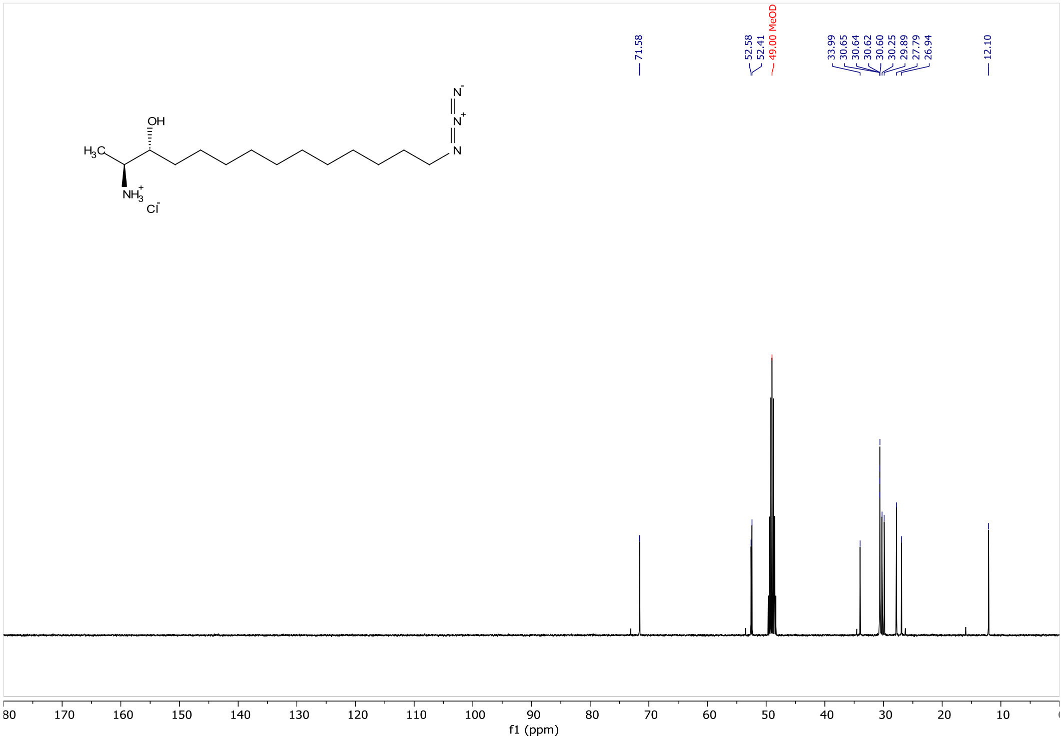

Figure S8. ^{1}H(400 MHz) and $^{13}C\{^{1}H\}$(101 MHz) NMR spectra of compound **7** in CD_3OD.

Compound 8

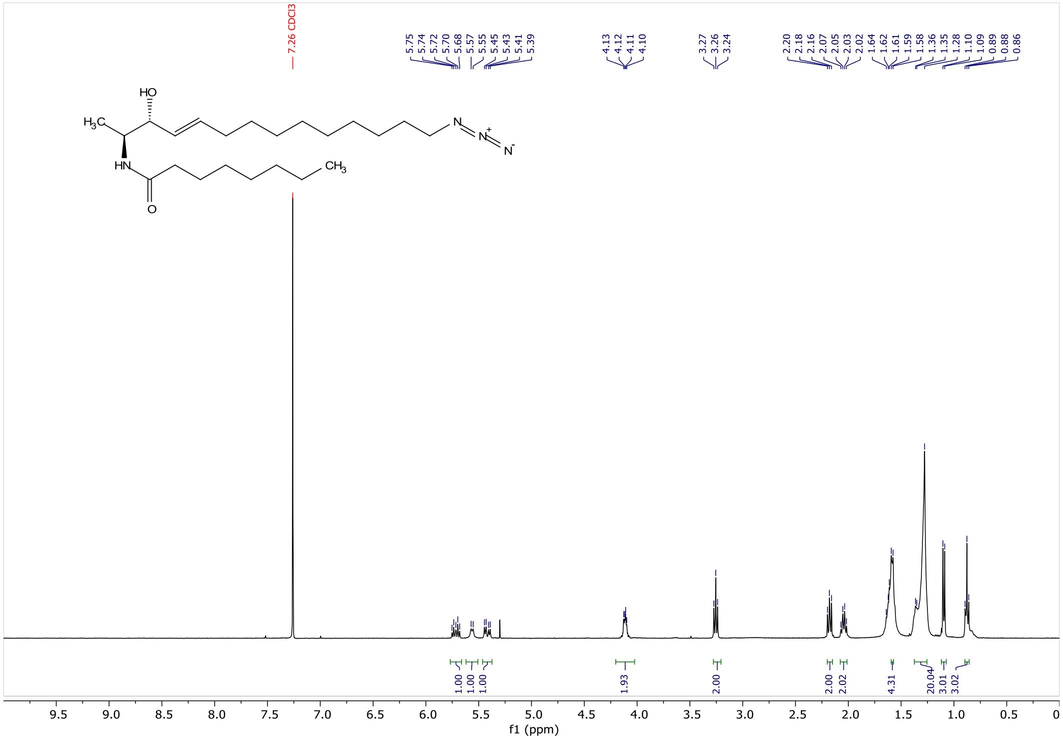

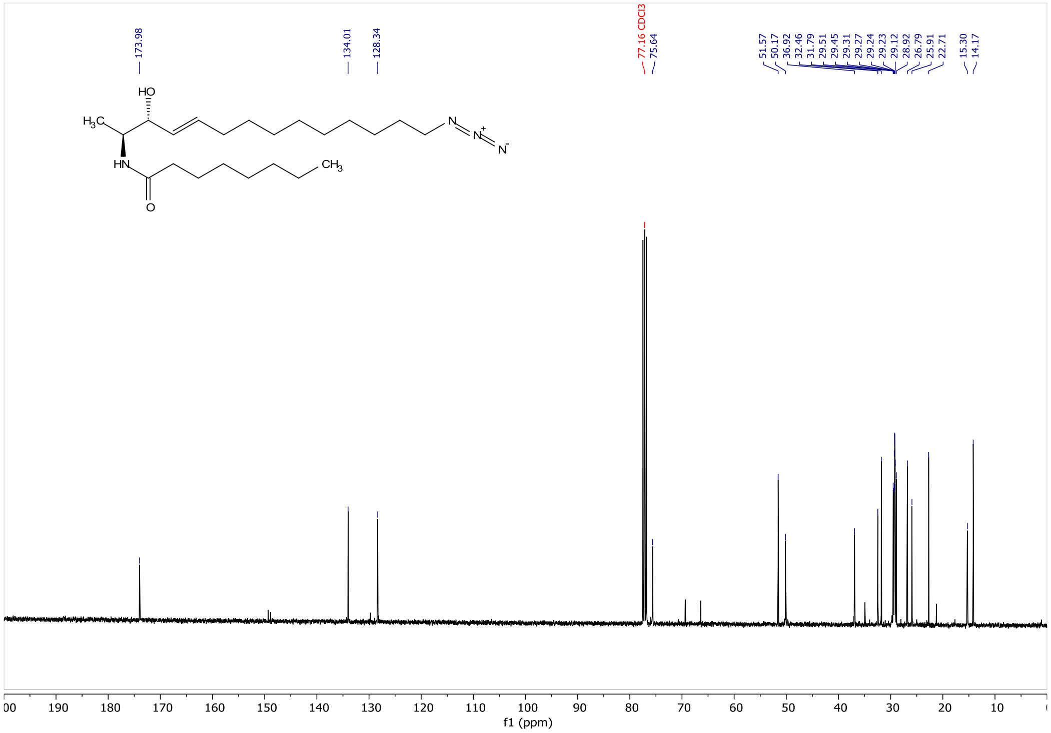

Figure S9. ^{1}H(400 MHz) and $^{13}C\{^{1}H\}$(101 MHz) NMR spectra of compound **8** in $CDCl_3$.

Compound 9

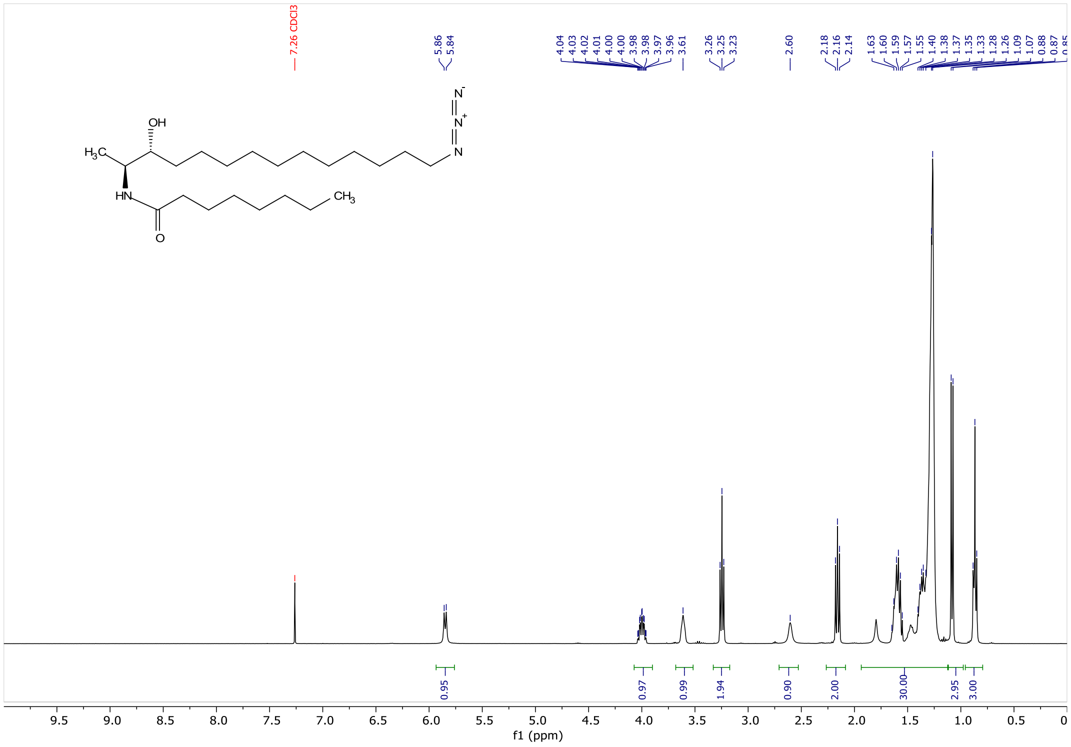

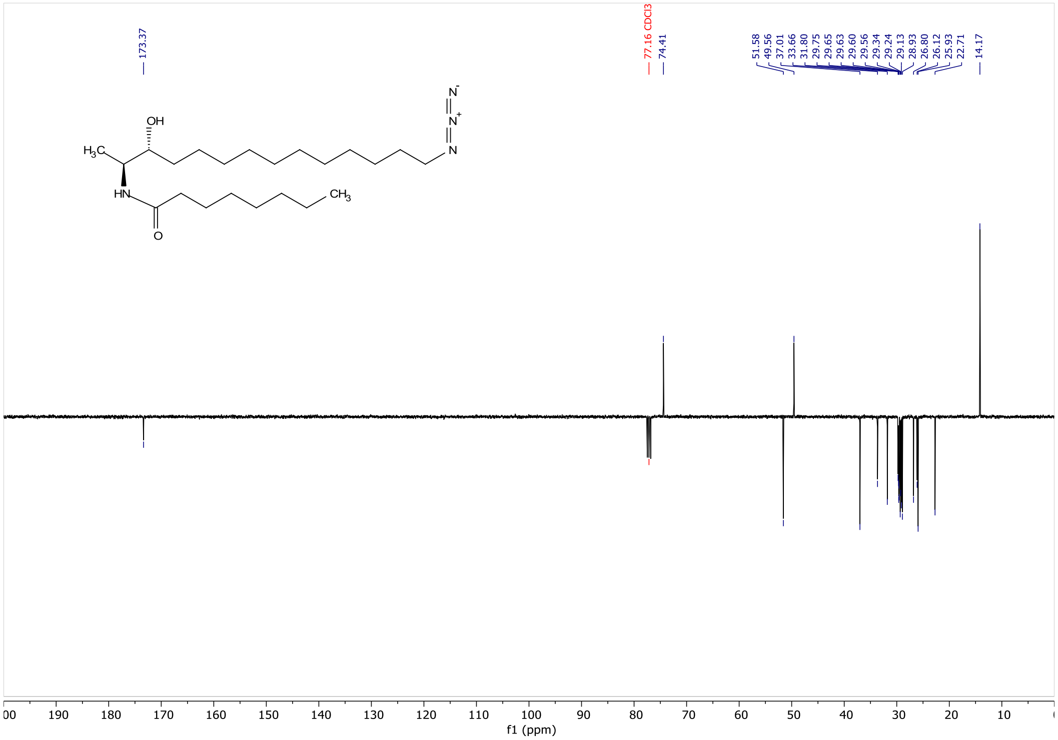

Figure S10. ^{1}H(400 MHz) and $^{13}C\{^{1}H\}$(101 MHz) APT NMR spectra of compound **9** in $CDCl_3$.

Compound 10

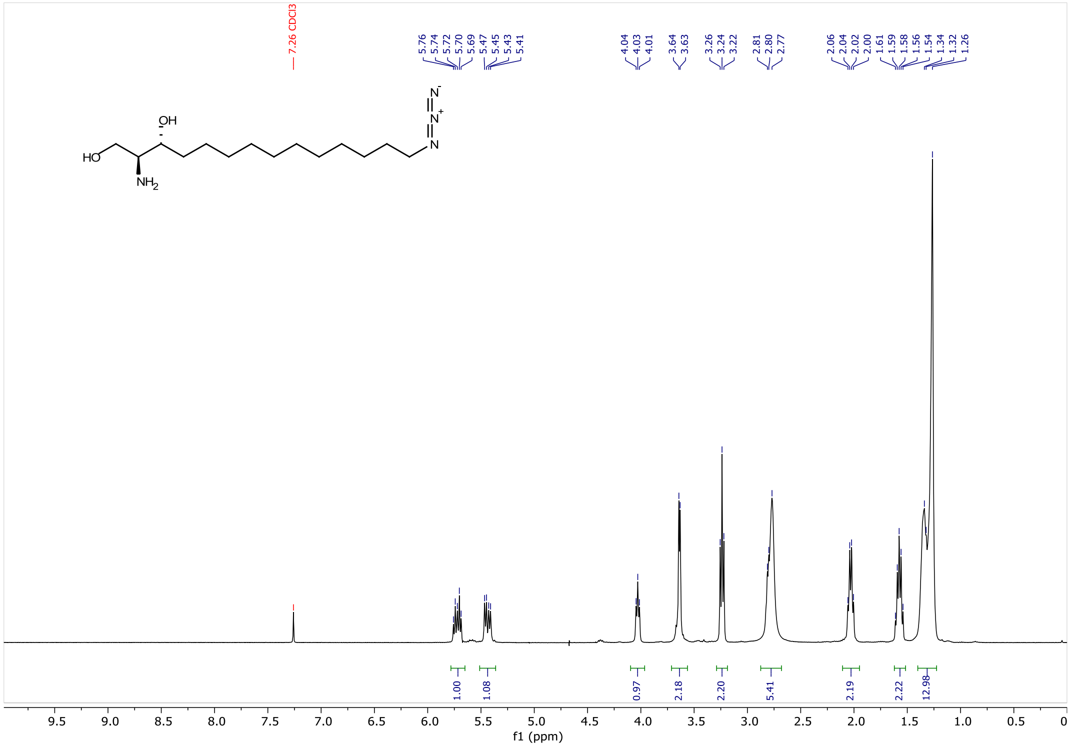

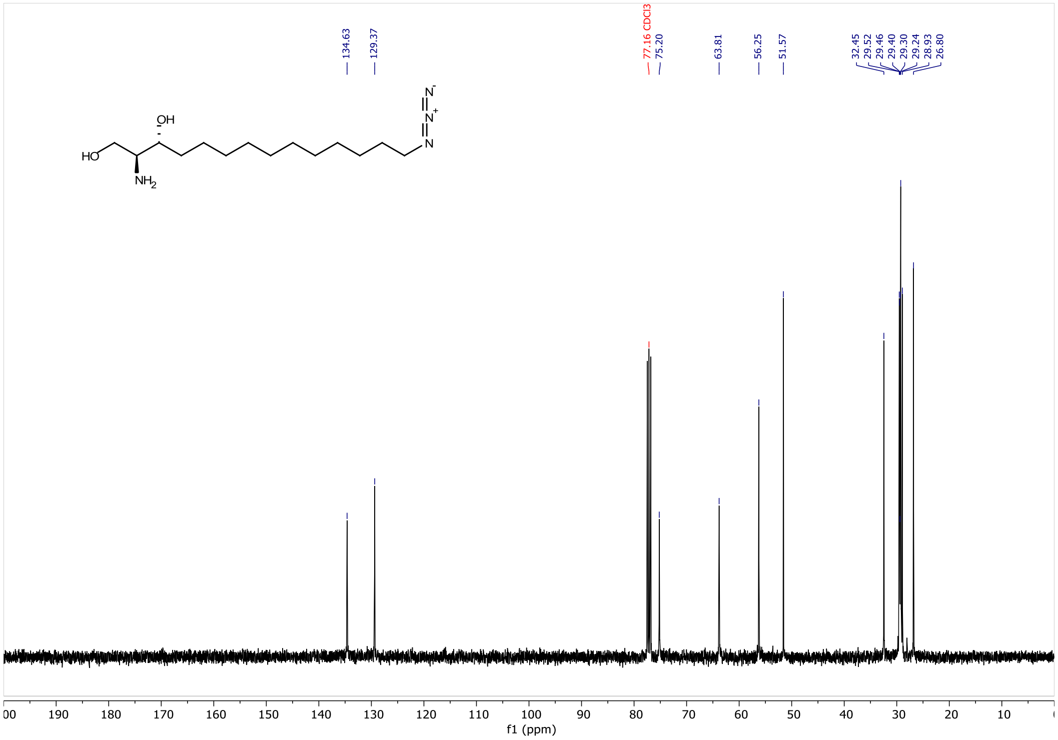

Figure S11. ^{1}H(400 MHz) and $^{13}C\{^{1}H\}$(101 MHz) NMR spectra of compound **10** in $CDCl_3$.

Compound 11

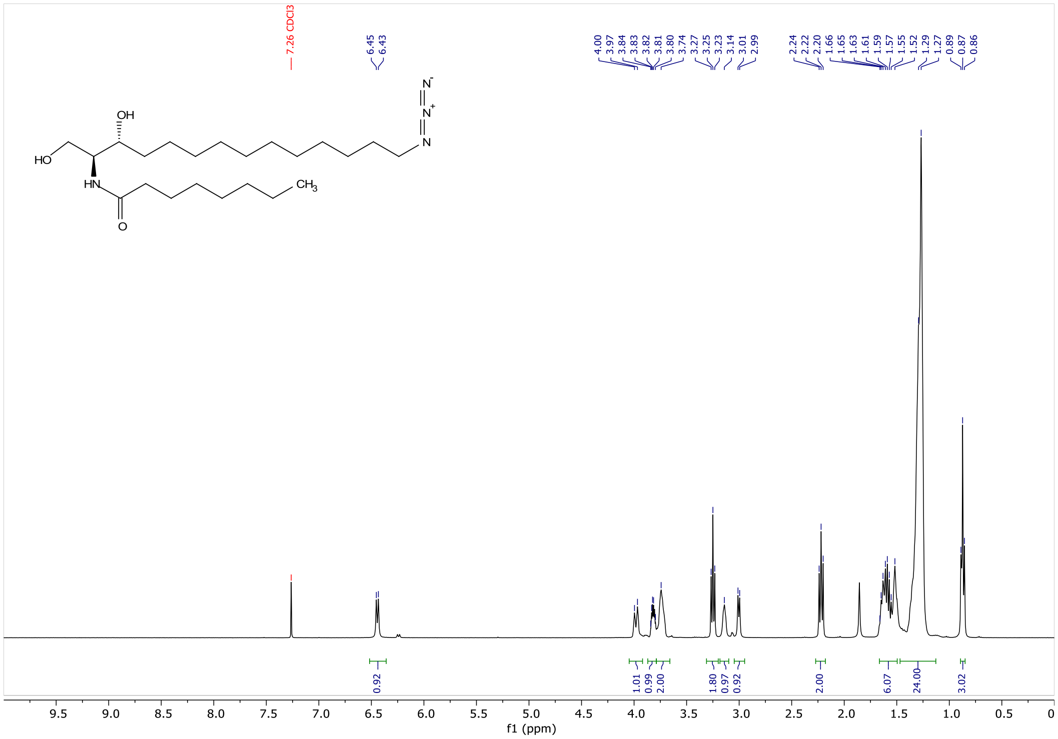

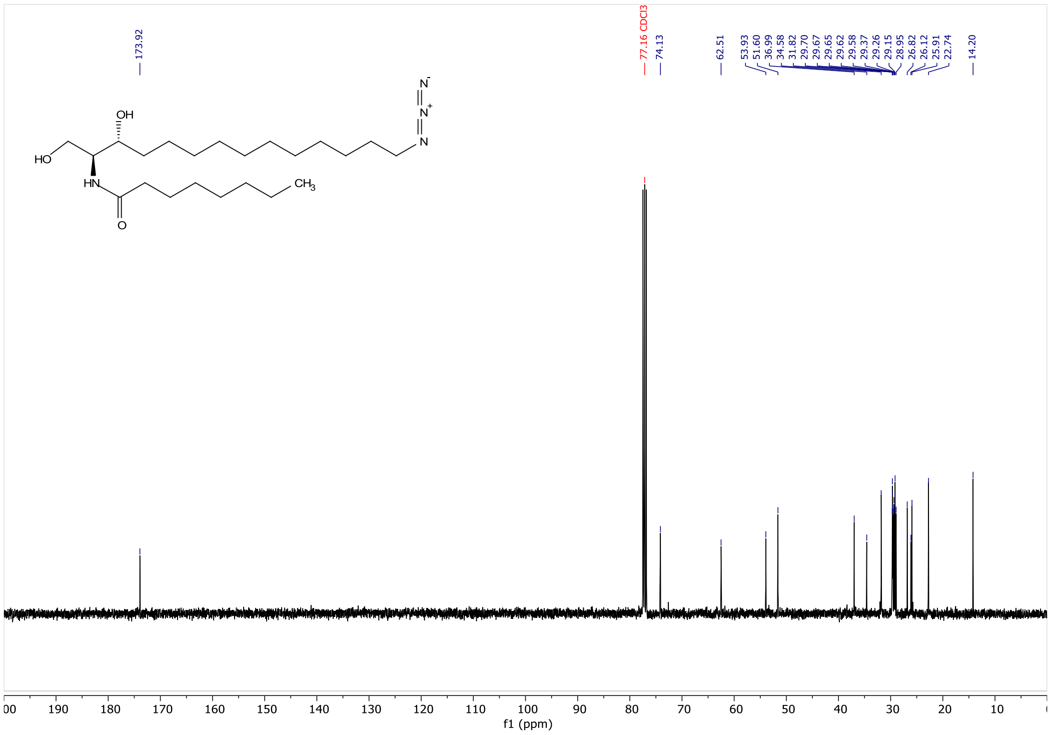

Figure S12. ^{1}H(400 MHz) and $^{13}C\{^{1}H\}$(101 MHz) spectra of compound **11** in $CDCl_3$.

Compound 12

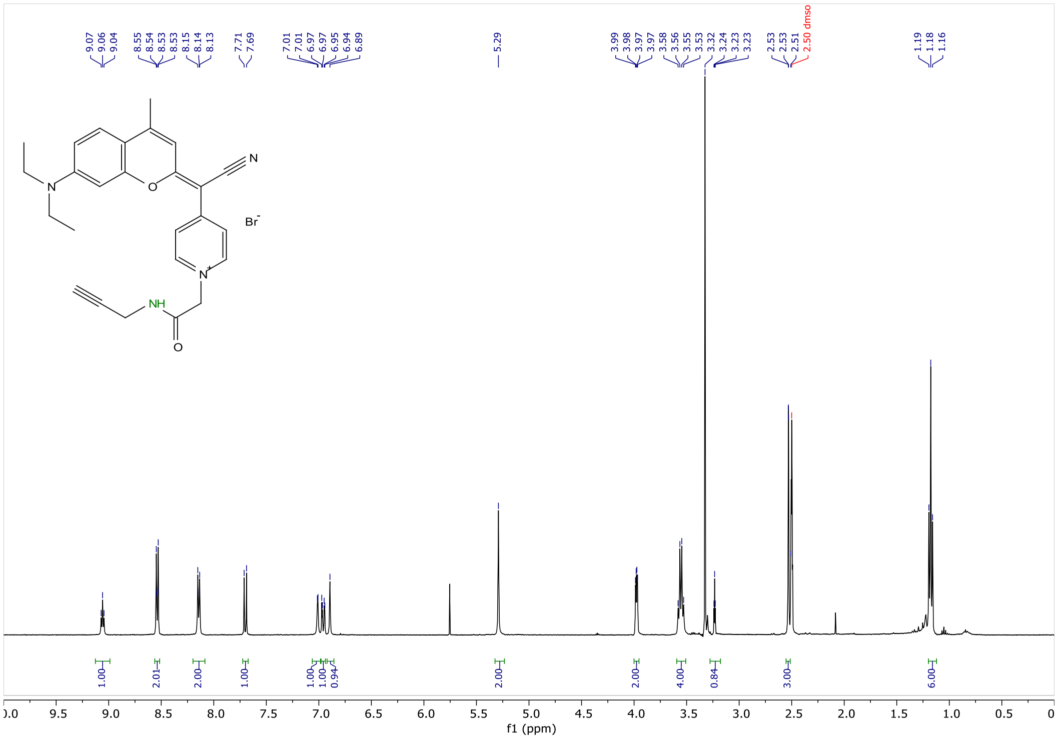

Figure S13. ^{1}H(400 MHz) spectrum of compound **12** in d_6-DMSO.

Probe COUPY-1

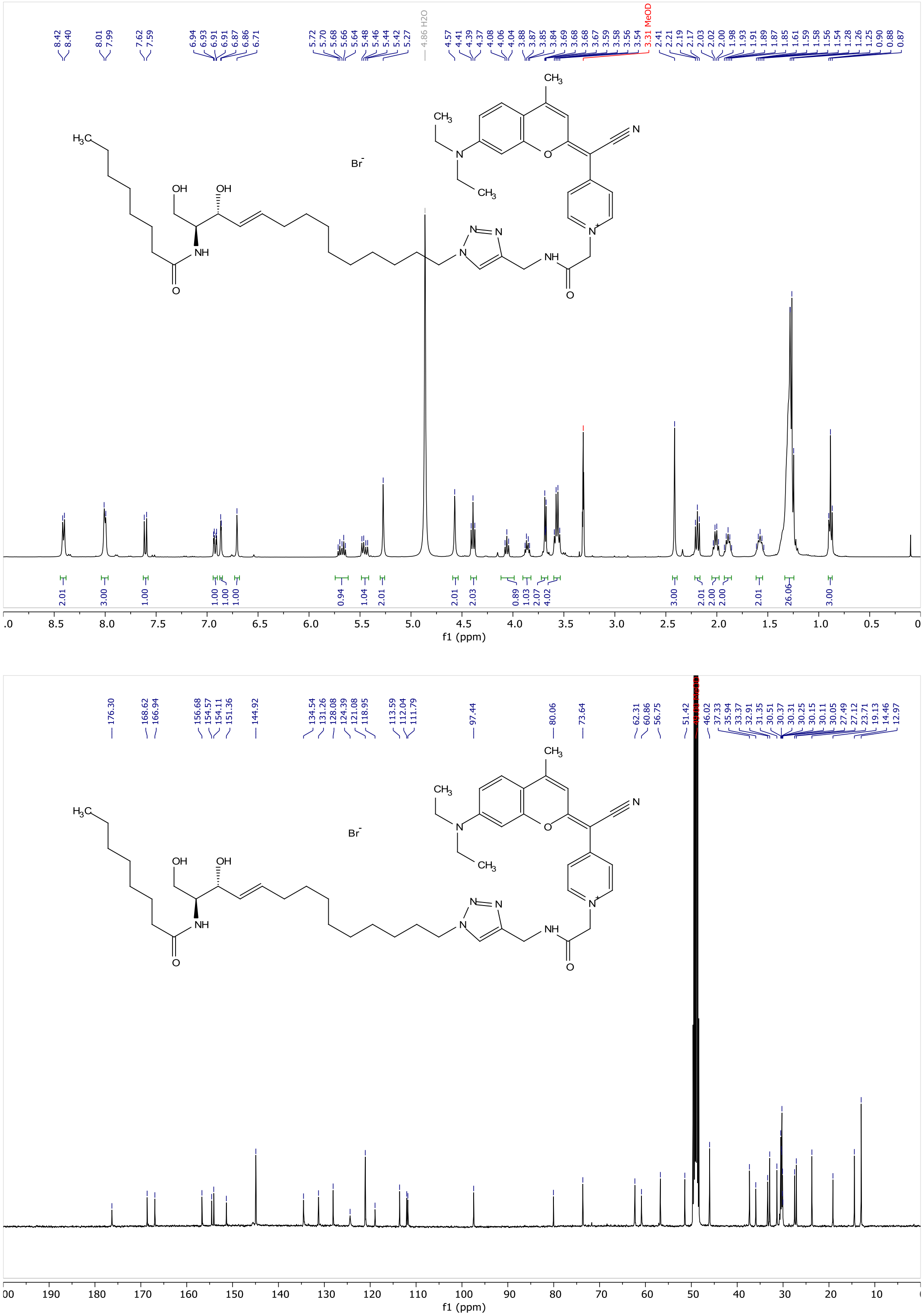

Figure S14. ^{1}H(400 MHz) and $^{13}C\{^{1}H\}$(101 MHz) NMR spectra of probe **COUPY-1** in CD_3OD.

Probe COUPY-2

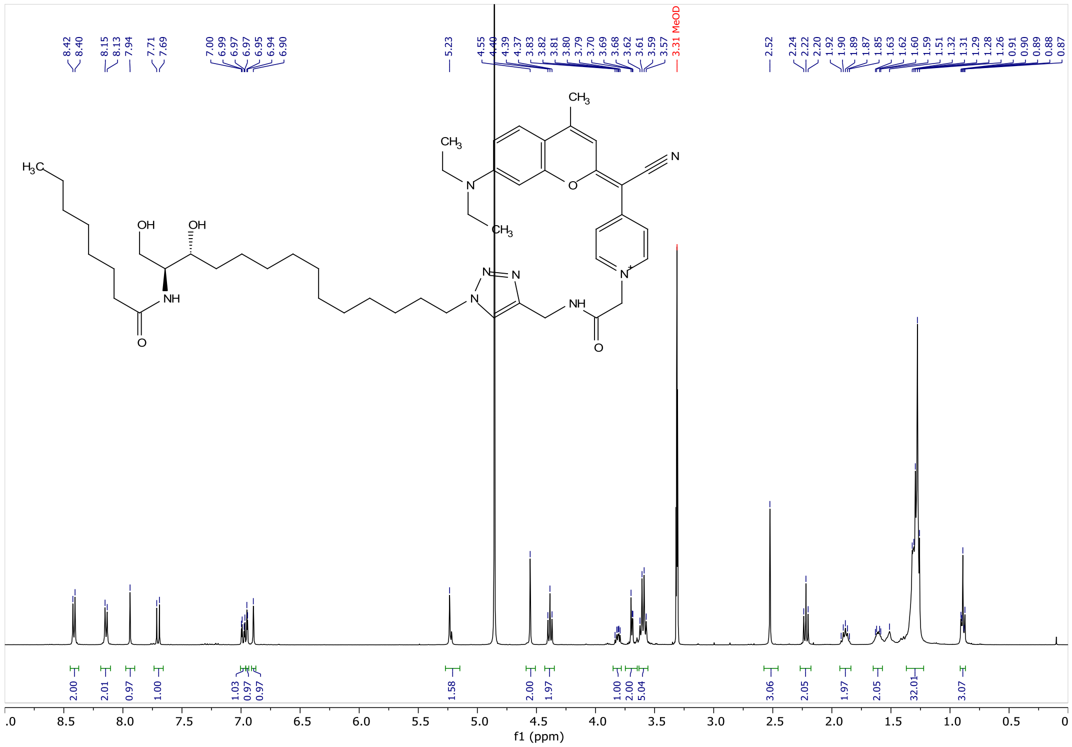

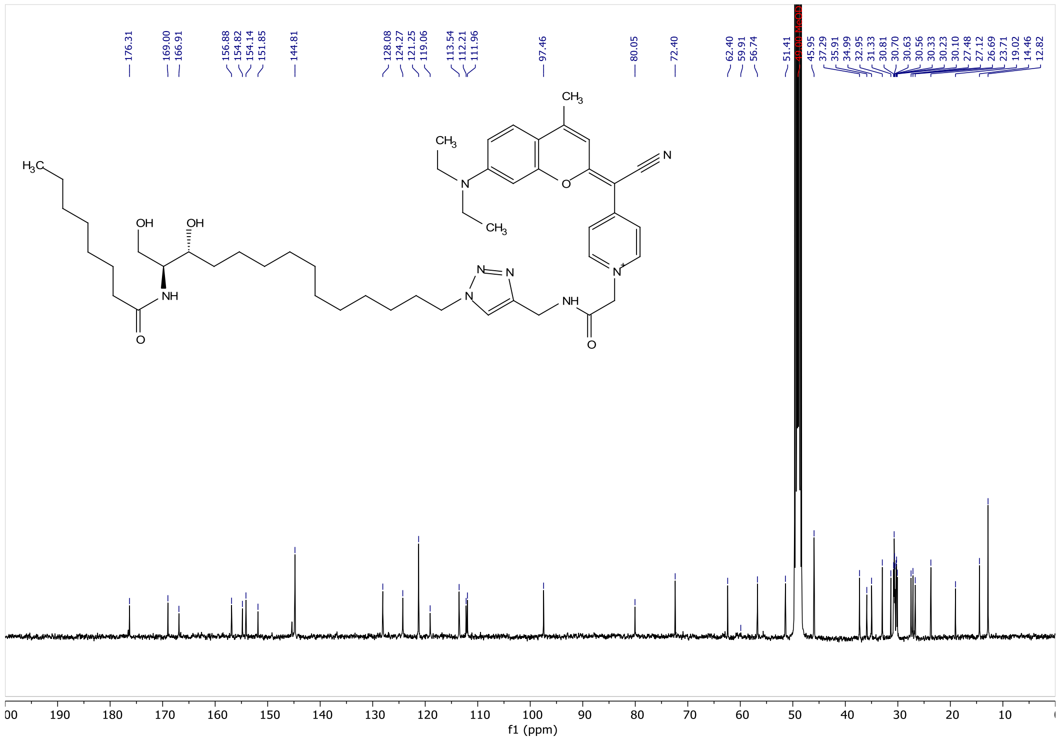

Figure S15. ^{1}H(400 MHz) and $^{13}C\{^{1}H\}$(101 MHz) NMR spectra of probe **COUPY-2** in CD_3OD.

Probe COUPY-3

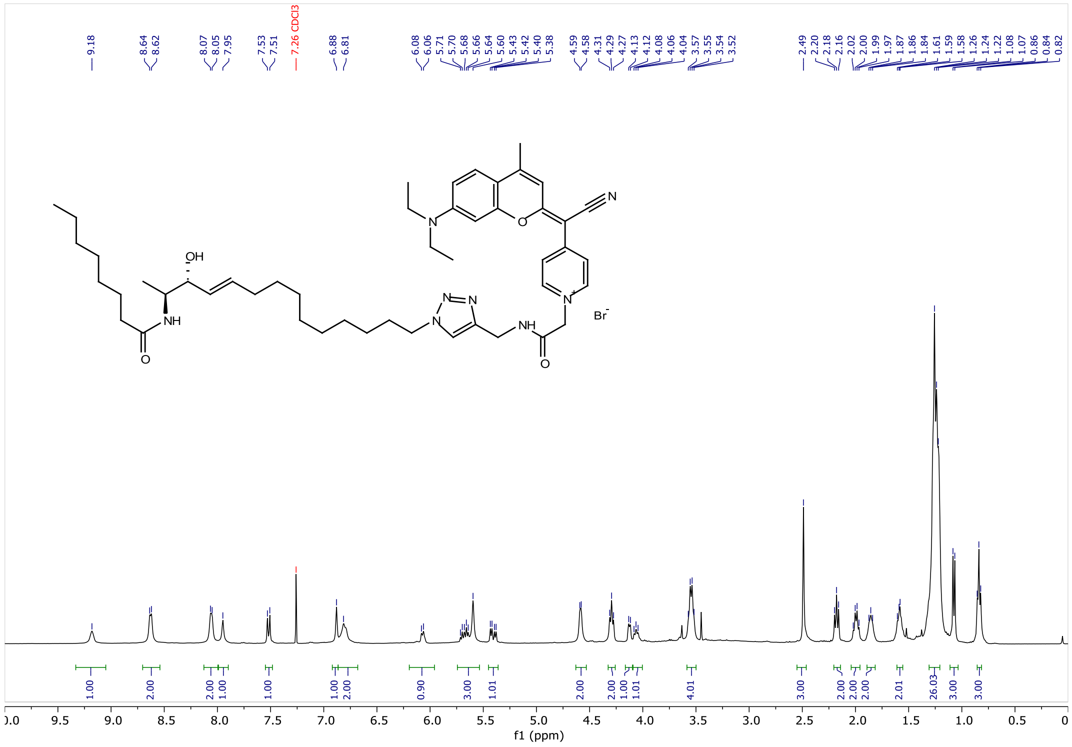

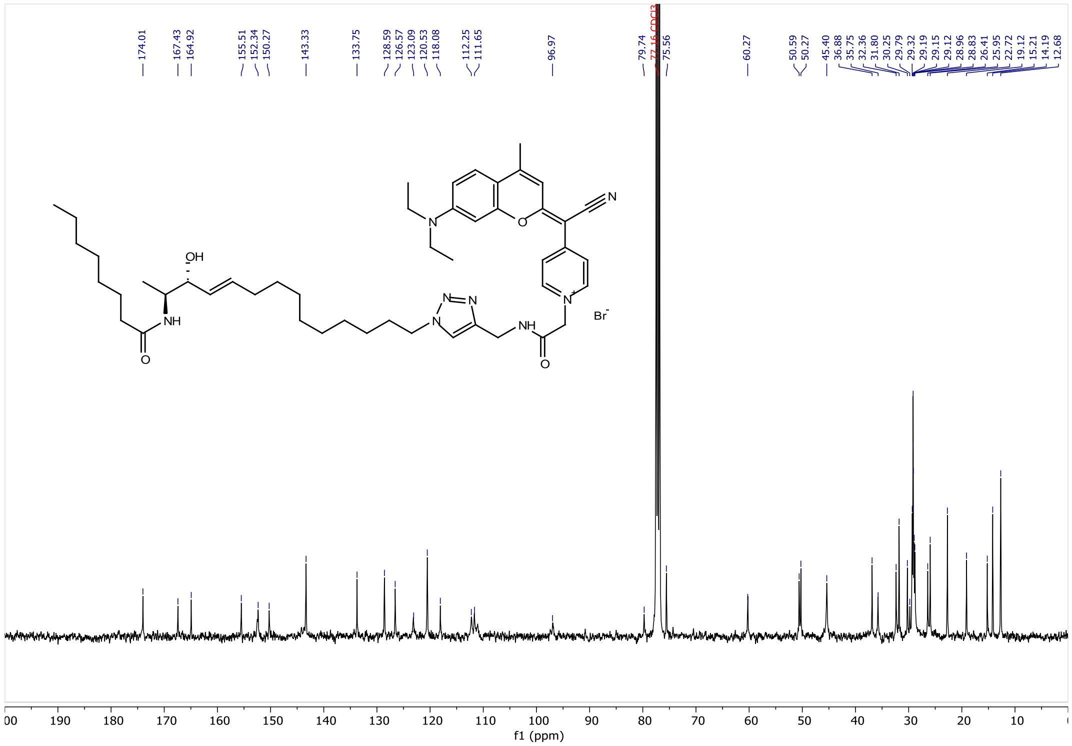

Figure S16. ^{1}H(400 MHz) and ^{13}C{^{1}H}(101 MHz) NMR spectra of probe **COUPY-3** in $CDCl_3$.

Probe COUPY-4

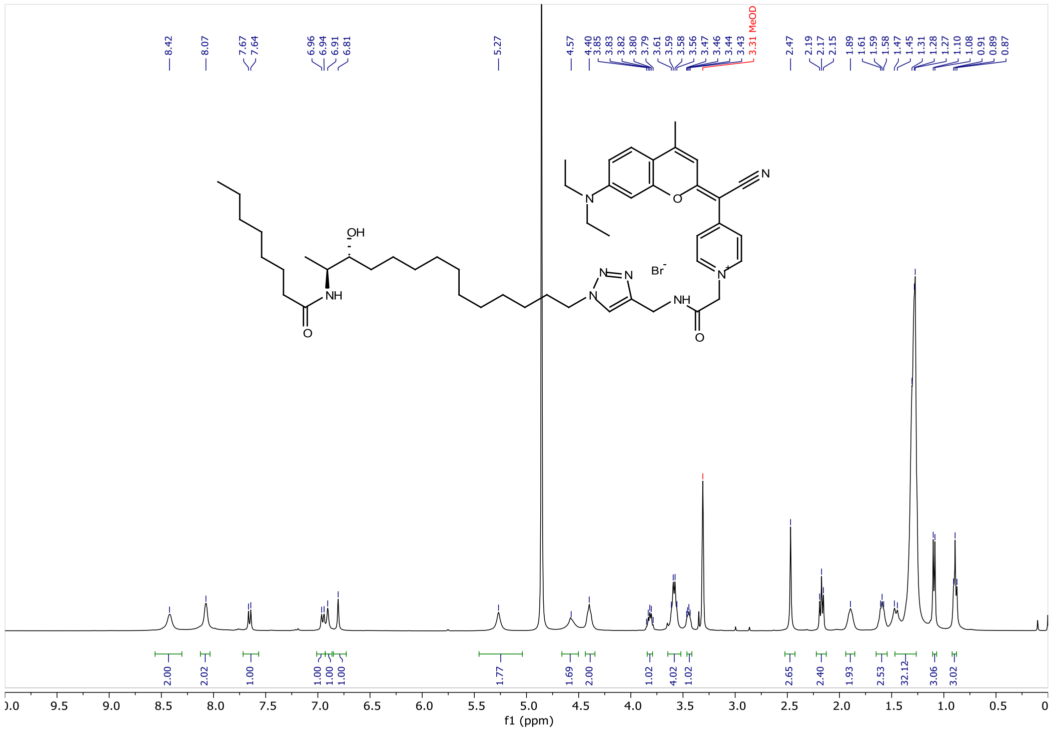

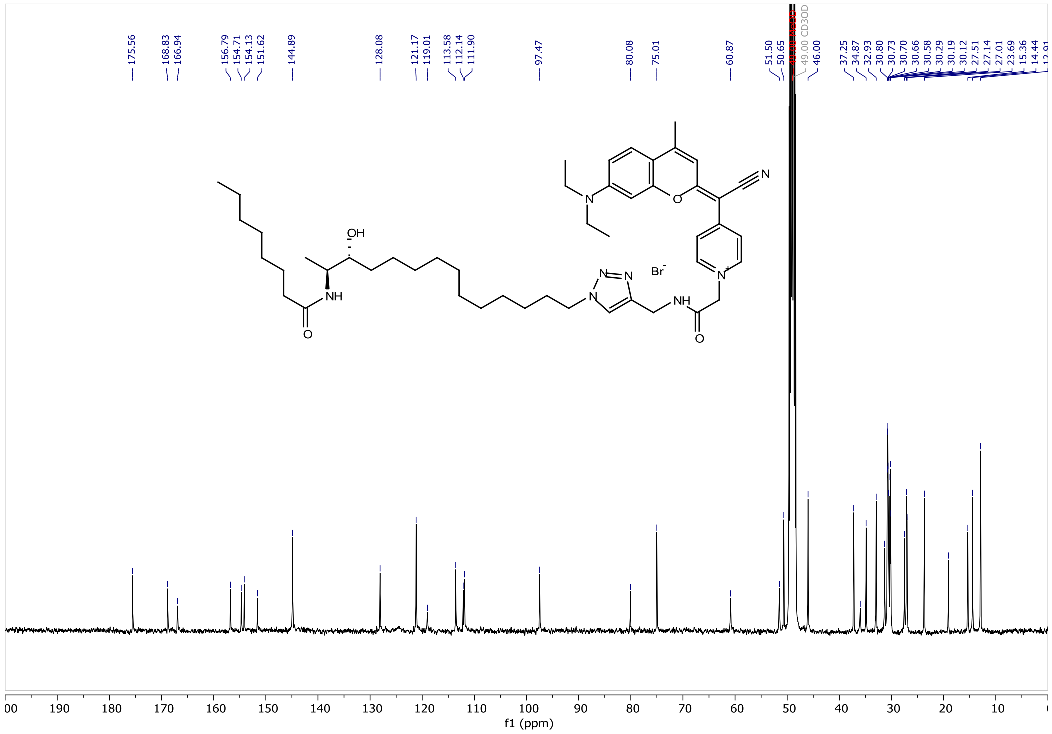

Figure S17. ^{1}H(400 MHz) and $^{13}C\{^{1}H\}$(101 MHz) NMR spectra of probe **COUPY-4** in CD_3OD.

Compound 13

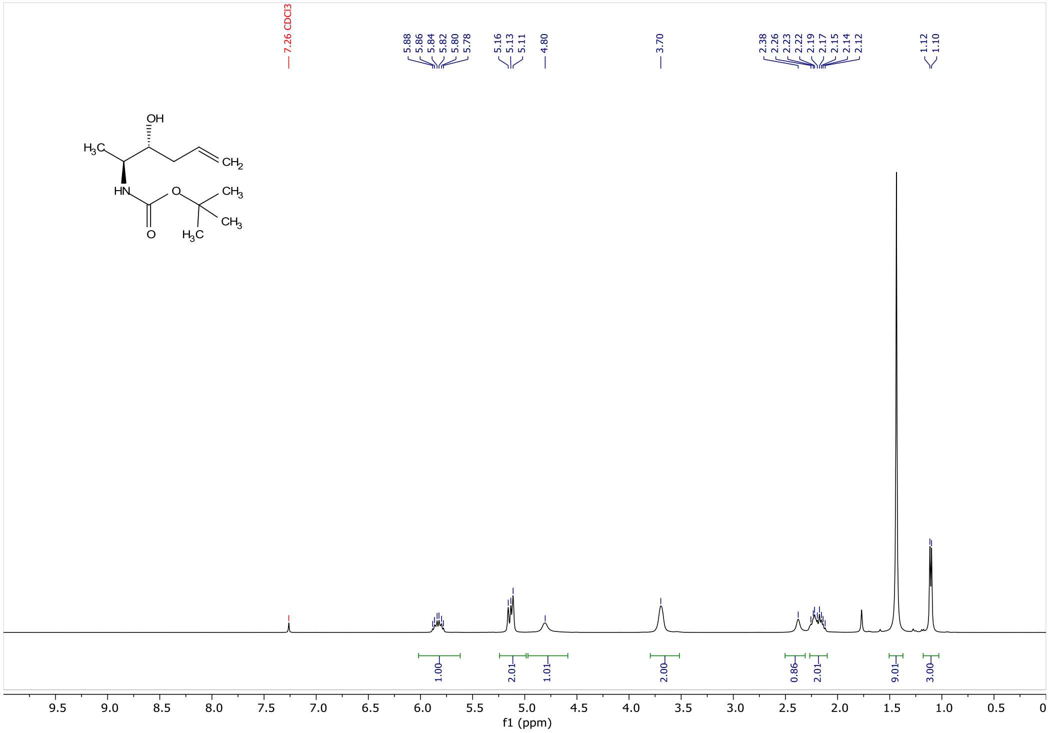

Figure S18. ^{1}H(400 MHz) NMR spectrum of compound **13** in $CDCl_3$.

Compound 14

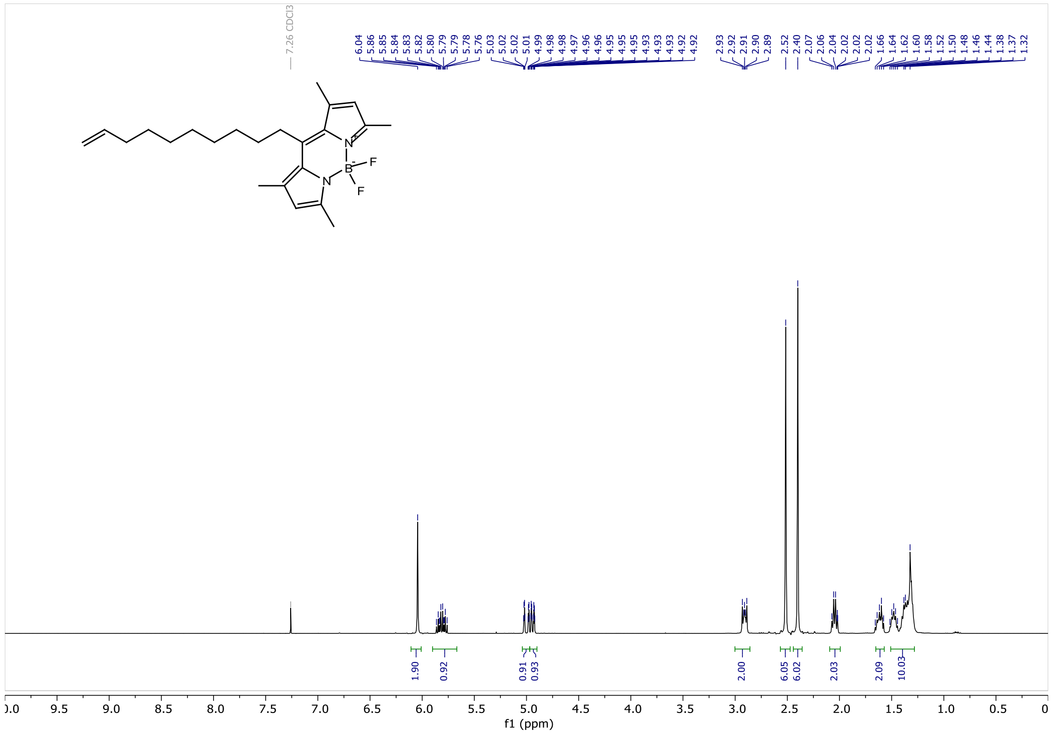

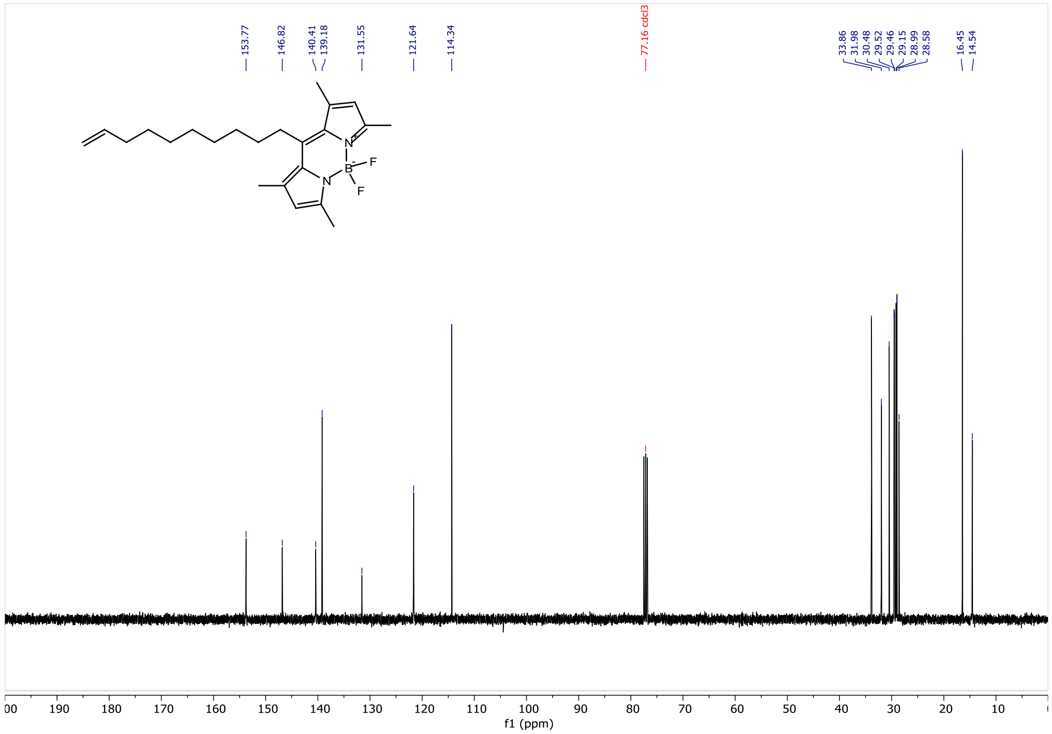

Figure S19. ^{1}H(400 MHz) and $^{13}C\{^{1}H\}$(101 MHz) NMR spectra of compound **14** in $CDCl_3$.

Compound 15

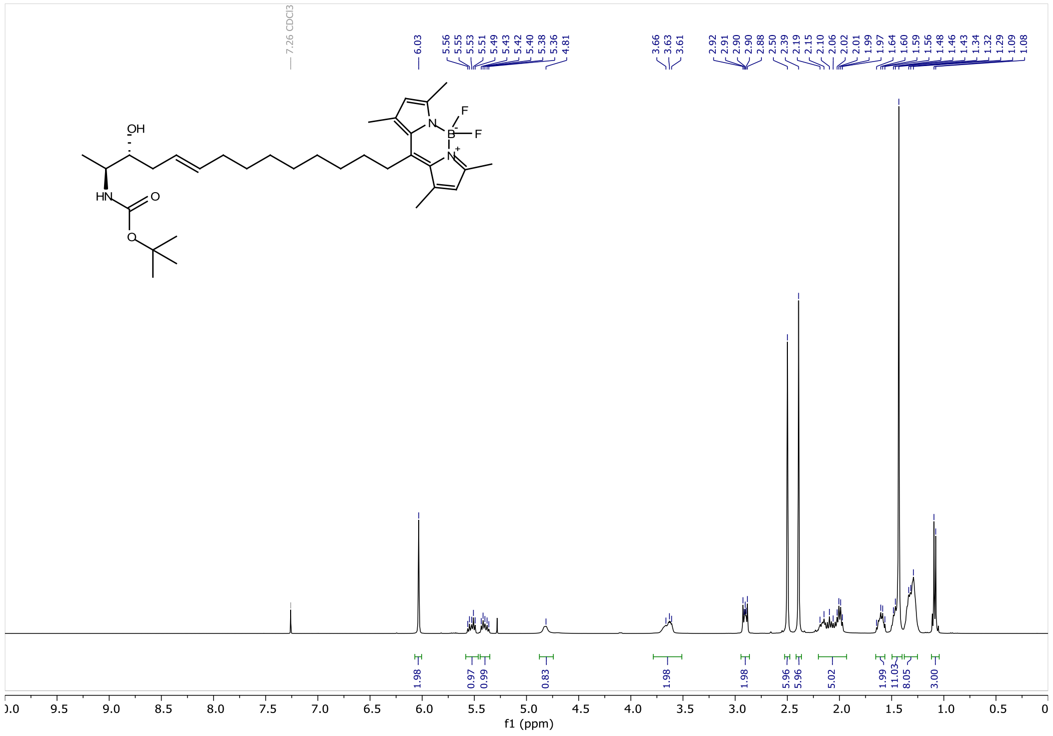

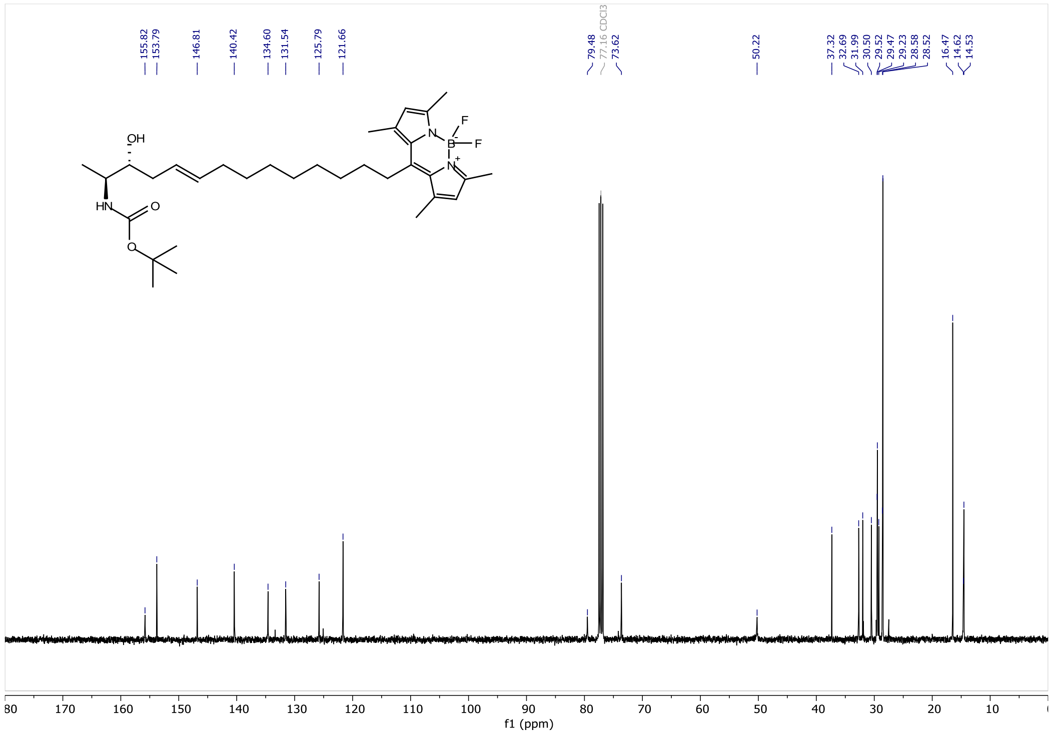

Figure S20. ^{1}H(400 MHz) and ^{13}C{^{1}H}(101 MHz) NMR spectra of compound **15** in $CDCl_3$.

Compound 16

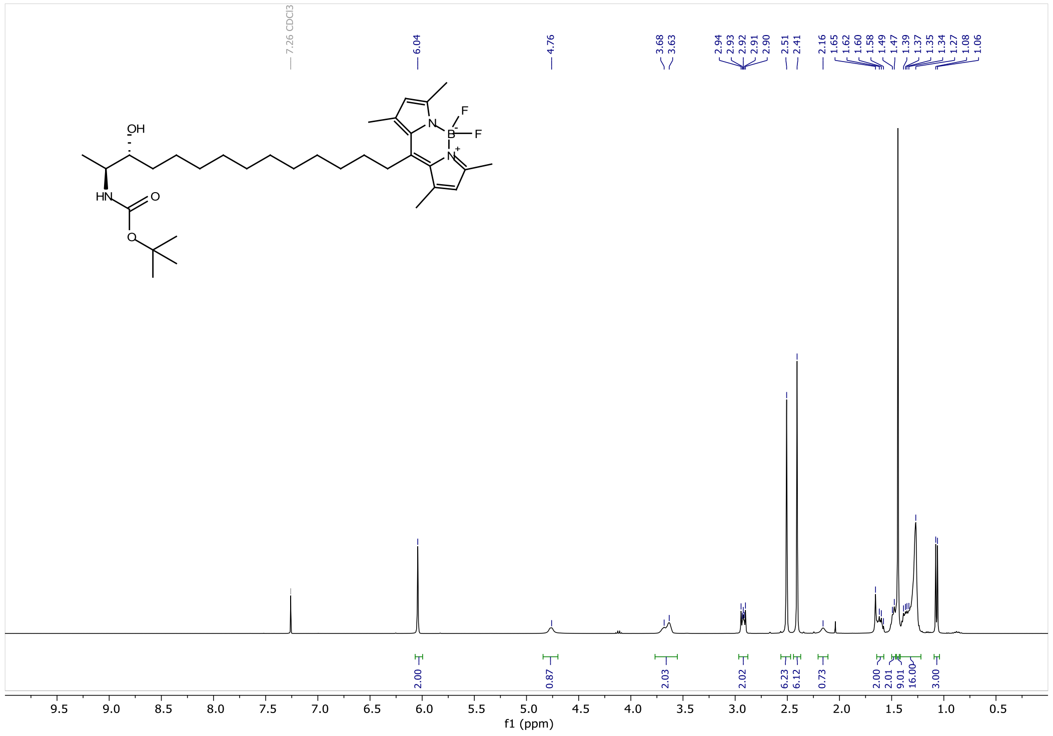

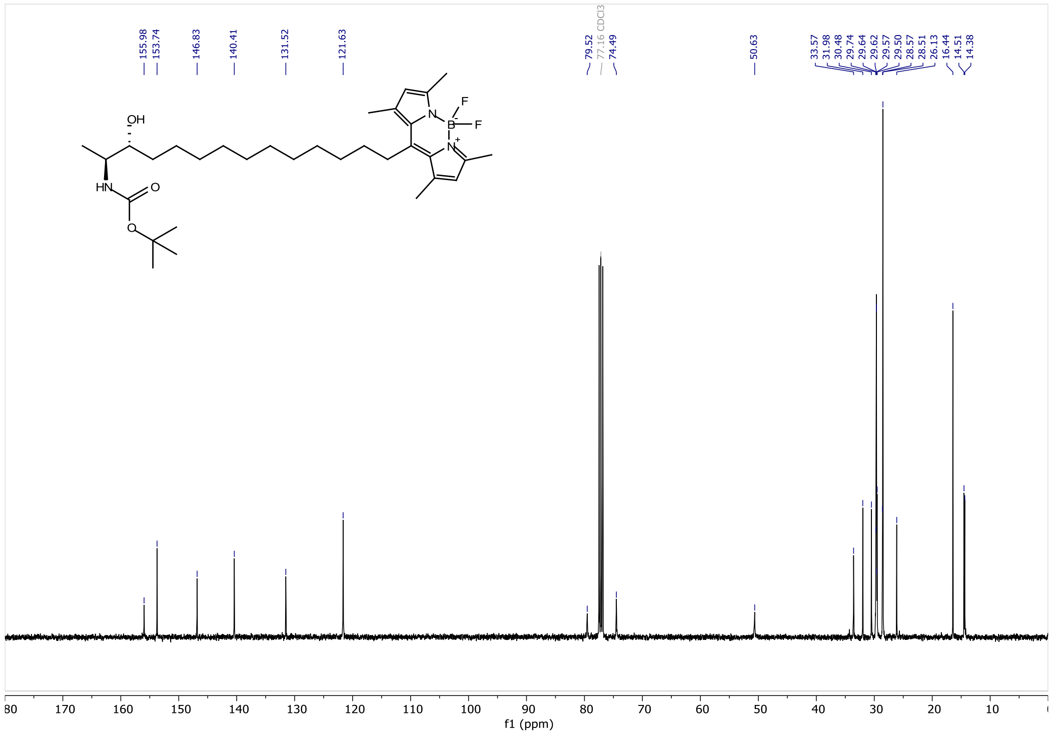

Figure S21. ^{1}H(400 MHz) and $^{13}C\{^{1}H\}$(101 MHz) NMR spectra of compound **16** in $CDCl_3$.

Compound 17

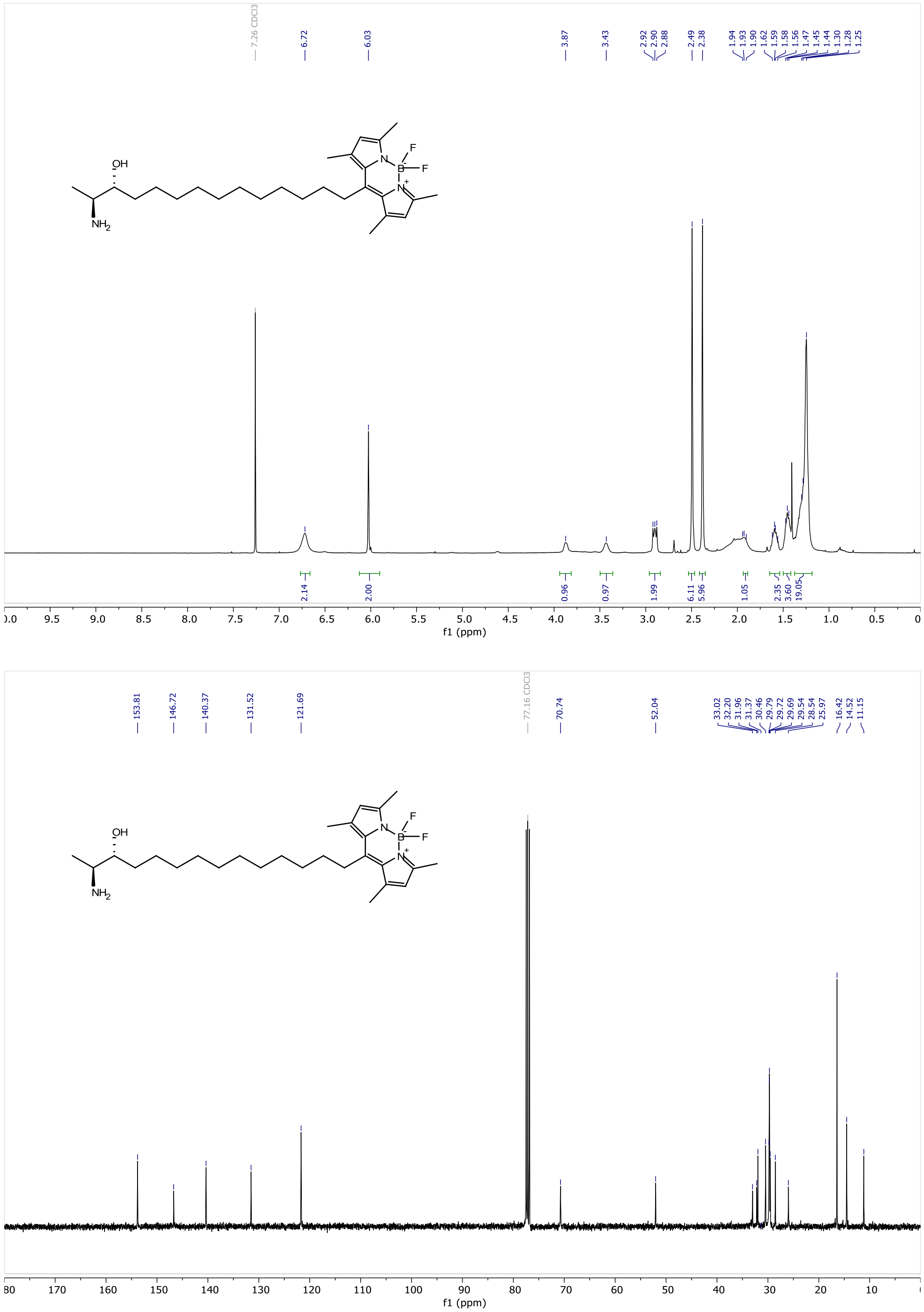

Figure S22. ^{1}H(400 MHz) and $^{13}C\{^{1}H\}$(101 MHz) NMR spectra of compound **17** in $CDCl_3$.

Compound 18

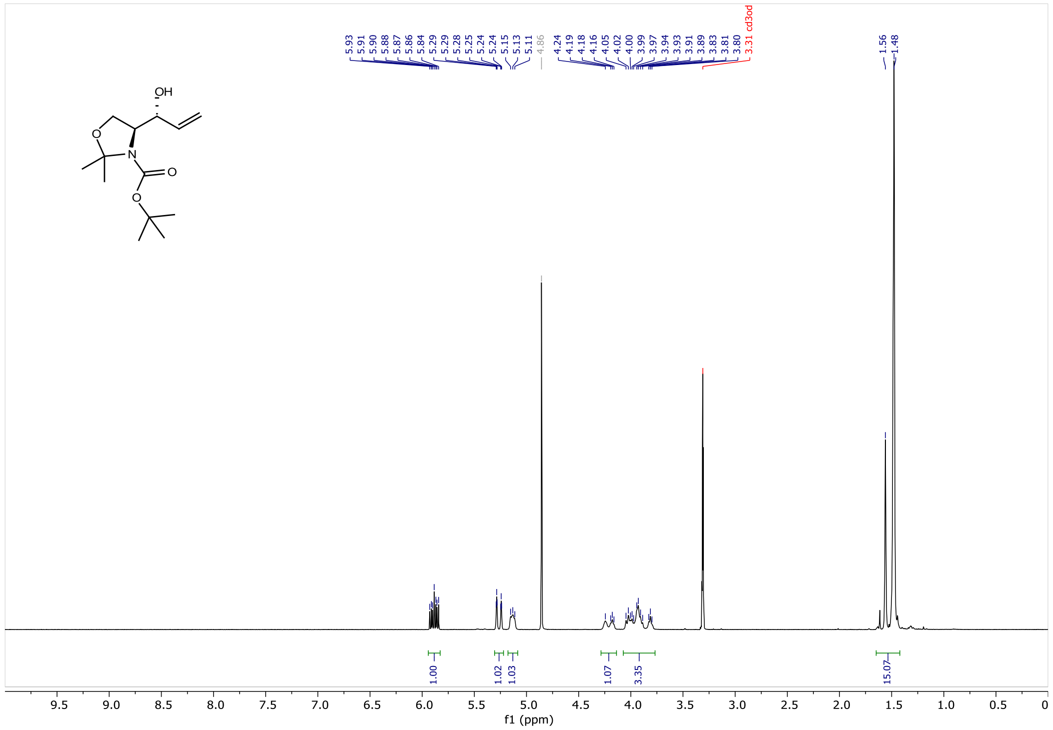

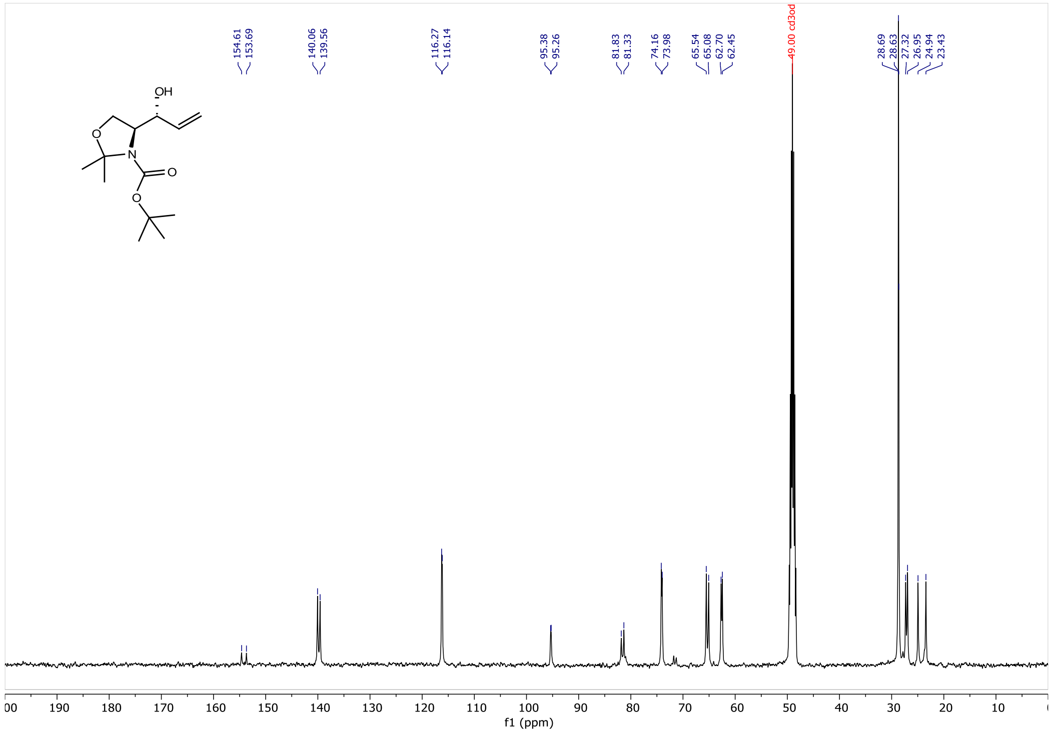

Figure S23. ^{1}H(400 MHz) and $^{13}C\{^{1}H\}$(101 MHz) NMR spectra of compound **18** in CD_3OD

Probe BODIPY-1

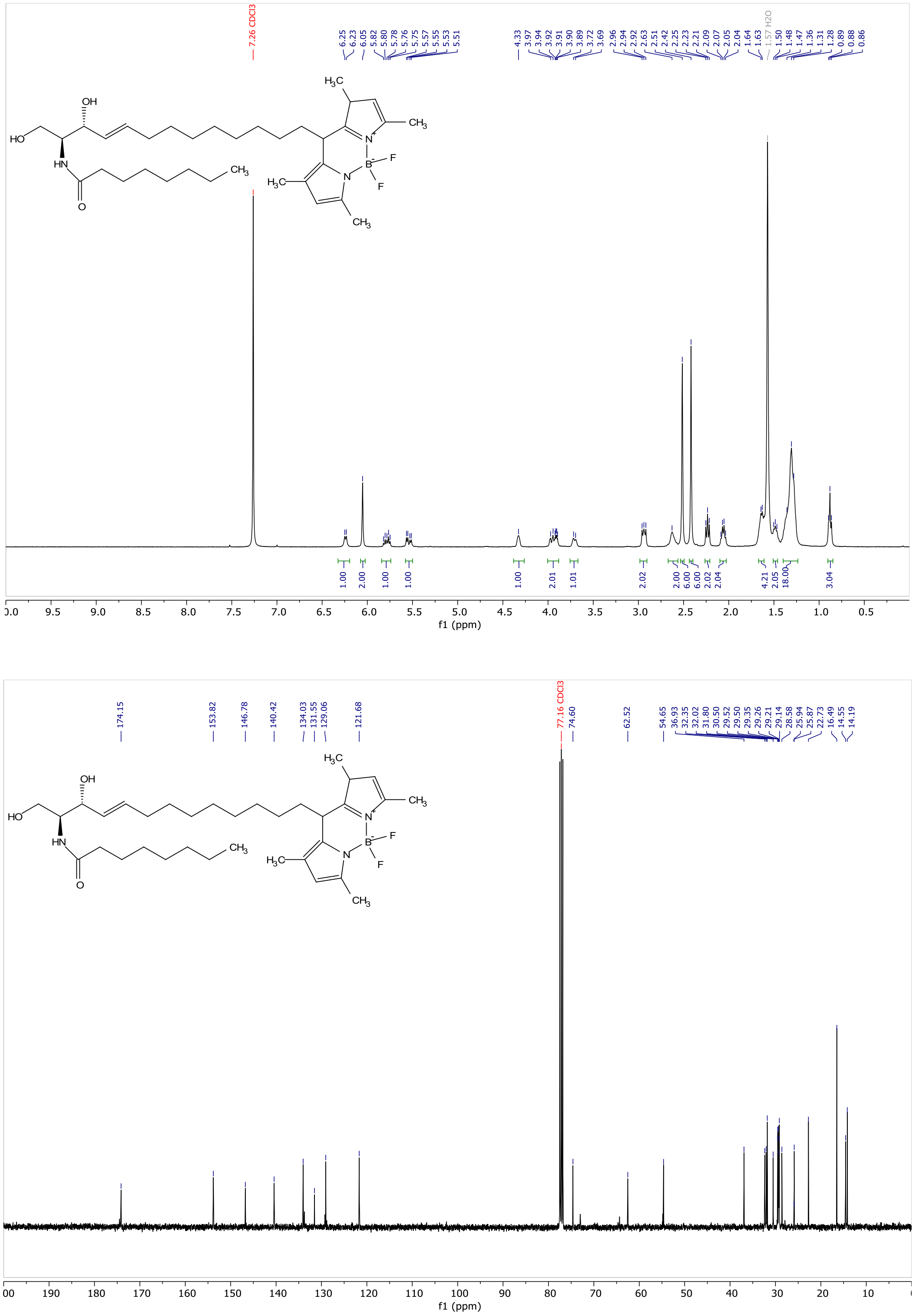

Figure S24. ^1H(400 MHz) and $^{13}C\{^1H\}$(101 MHz) NMR spectra of probe **BODIPY-1** in $CDCl_3$.

Probe BODIPY-4

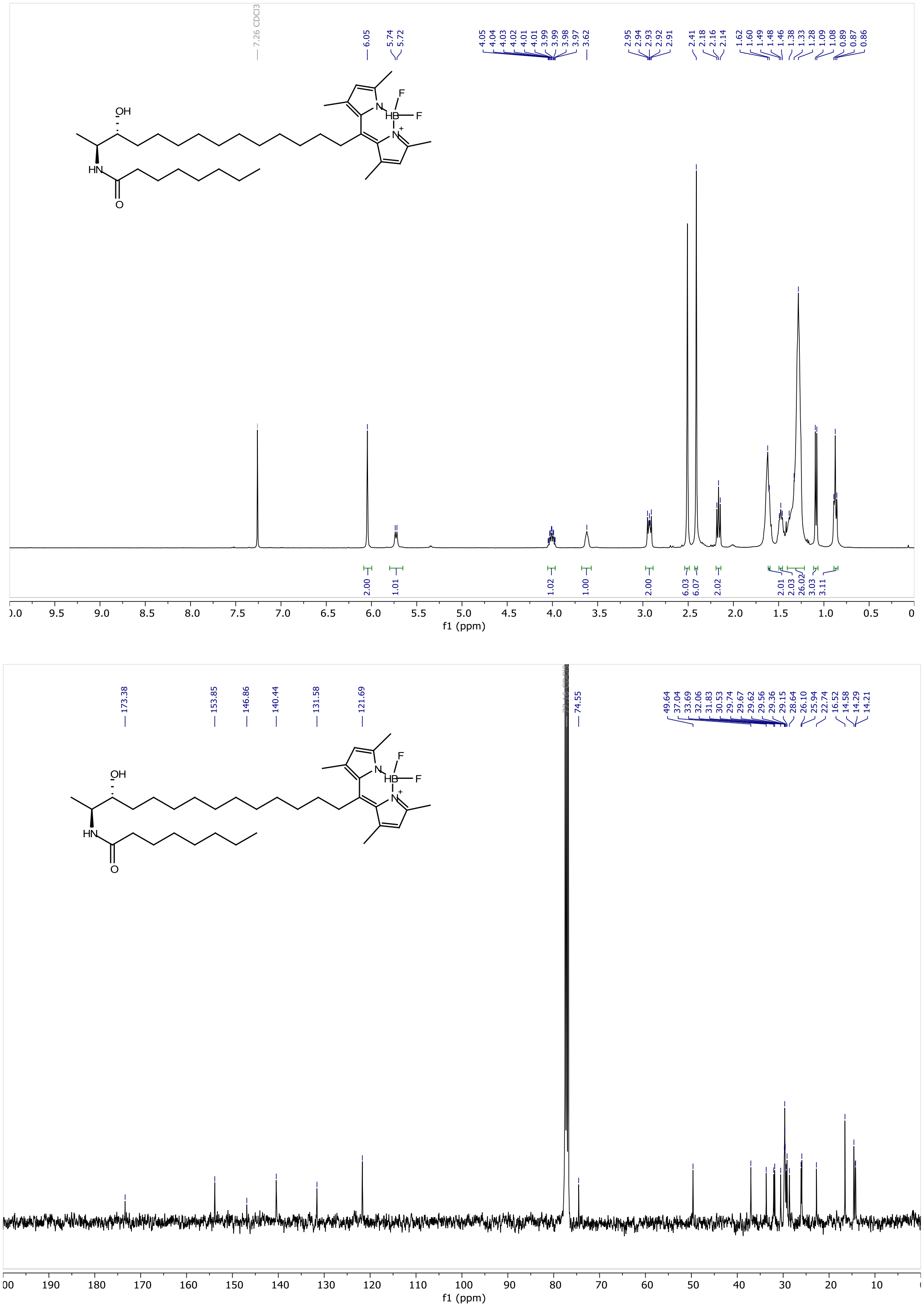

Figure S25. ^{1}H(400 MHz) and $^{13}C\{^{1}H\}$(101 MHz) NMR spectra of probe **BODIPY-4** in $CDCl_3$.
